# Nasal steroids, irrigation, oral antibiotics, and subgroup targeting for effective management of acute sinusitis (NOSES): Protocol for a comparative effectiveness randomized controlled trial

**DOI:** 10.1371/journal.pone.0348522

**Published:** 2026-05-06

**Authors:** Daniel Merenstein, Nawar Shara, David P. Rabago, Derjung Mimi Tarn, Bruce Barrett, Alex H. Krist, Sebastian T. Tong, Aleksandra E. Zgierska, Tina P. Tan, Keisha Herbin Smith, Gabriela Villalobos, Charles R. Fencil, Kavya K. Sanghavi, Syed Junaid Imam, Stephen Fernandez, Mihriye Mete

**Affiliations:** 1 Clinical Coordinating Center, Department of Family Medicine, Georgetown University Medical Center, Washington, District of Columbia, United States of America; 2 Data Coordinating Center, MedStar Health Research Institute, Columbia, Maryland, United States of America; 3 Department of Family and Community Medicine, Penn State College of Medicine, Hershey, Pennsylvania, United States of America; 4 Department of Family Medicine, David Geffen School of Medicine at UCLA, Los Angeles, California, United States of America; 5 Department of Family Medicine and Community Health, University of Wisconsin-Madison, Madison, Wisconsin, United States of America; 6 Department of Family Medicine and Population Health, Virginia Commonwealth University, Richmond, Virginia, United States of America; 7 Department of Family Medicine, University of Washington, Seattle, Washington, United States of America; PLOS: Public Library of Science, UNITED KINGDOM OF GREAT BRITAIN AND NORTHERN IRELAND

## Abstract

**Introduction:**

Acute rhinosinusitis (ARS) affects approximately 15% of adults annually. It accounts for nearly 30 million outpatient visits and one in five antibiotic prescriptions, and contributes to over $11 billion in direct healthcare costs in the United States. Because routine clinical evaluation cannot reliably distinguish viral from bacterial ARS, antibiotics are frequently prescribed for infections that are likely viral. Non-antibiotic therapies such as intranasal corticosteroids (INCS) and saline nasal irrigation may provide symptom relief, but their comparative effectiveness in ARS has not been rigorously evaluated.

**Objectives:**

This protocol describes the design and methodology of a large, multi-center, pragmatic randomized controlled trial to evaluate antibiotic and non-antibiotic treatment strategies for ARS. The trial will compare supportive care, and clinical and laboratory indicators that may identify subgroups most likely to benefit from specific therapies.

**Methods:**

This placebo-controlled trial is underway in primary care and urgent care settings in six regions across the U.S. Adults aged 18–75 years with ARS initially receive clinician-directed care. Participants who do not improve after 9 days of symptoms will be randomized into one of four groups: antibiotics alone; placebo-antibiotics plus INCS; antibiotics plus INCS; or placebo-antibiotics alone. All participants receive educational materials and kits for saline nasal irrigation. The trial will enroll 3,720 participants, with approximately 60% expected to enter the randomized phase. Primary outcomes include symptom severity prior to and 3 days post-randomization, as measured by the modified Sino-Nasal Outcome Test-16).

**Significance:**

This trial will generate high-quality evidence to inform targeted treatment of ARS, identify subgroups most likely to benefit from antibiotics or non-antibiotic therapies, and support more judicious antibiotic stewardship in routine clinical practice.

## Introduction

Acute rhinosinusitis (ARS) affects approximately 15% of adults annually and accounts for nearly 20% of all antibiotic prescriptions in outpatient care [1–8]. In the United States, one in seven adults—with approximately 30 million office visits each year—is diagnosed with ARS, resulting in one in five antibiotic prescriptions and more than $11 billion in direct annual healthcare costs [1–5,7,9]. Reducing inappropriate antibiotic prescribing for ARS is therefore critical to limiting the development and spread of antimicrobial resistance [10]. The World Health Organization has identified antibiotic overuse and resistance as a major global public health threat, and the United Nations has convened a high-level meeting to address antimicrobial resistance, making it only the fourth health issue ever considered by the General Assembly [11,12].

Multiple stakeholders have prioritized reducing unnecessary antibiotic use and promoting alternative management strategies for ARS. The *Get Smart: Know When Antibiotics Work* program at the Centers for Disease Control and Prevention identified ARS as the leading cause of inappropriate outpatient antibiotic prescribing [13]. Similarly, the Infectious Diseases Society of America (IDSA), the American Academy of Family Physicians (AAFP), and the Choosing Wisely® campaign have all recommended more judicious antibiotic use for ARS [14–16].

The consequences of inappropriate antibiotic use extend beyond societal concerns to individual patient harm. Prior work by our group and others has demonstrated that antibiotic exposure can lead to significant alterations in the microbiome and metabolic pathways, with potential long-term health implications for individuals [17–21]. Antibiotics are also among the most common causes of adverse drug events leading to emergency department visits, accounting for approximately 16% of all such reactions in the emergency room [22].

Unlike other common infections encountered in primary care—such as pharyngitis, urinary tract infections, or pneumonia—routine laboratory testing and physical examination findings cannot reliably distinguish viral from bacterial ARS [1,23–26]. In a prior meta-analysis of nine randomized controlled trials (RCTs) involving patients with clinically-diagnosed ARS, we found that commonly used signs and symptoms did not reliably identify subgroups more likely to benefit from antibiotic therapy [27–35].

Non-antibiotic treatments, including intranasal corticosteroids (INCS) and saline nasal irrigation (SNI), show promise for symptom relief in ARS. INCS has demonstrated benefit in selected patient subgroups, while SNI is widely recommended as adjunctive therapy for ARS despite limited evidence from large-scale effectiveness trials [32,36]. There is a need for large, pragmatic RCTs to evaluate the comparative effectiveness of antibiotic and non-antibiotic strategies, and to identify clinical and laboratory indicators—such as C-reactive protein (CRP) levels or symptom patterns, including “double-sickening”—that can predict treatment needs and response. This manuscript describes the design and methods of the largest RCT that aims to address these gaps and inform evidence-based, targeted clinical management of ARS.

## Methods

### Ethics statement

The study will be conducted in accordance with US Code of Federal Regulations (45 CFR Part 46) and ICH Good Clinical Practice (ICH GCP) E6(R2). All participating university Institutional Review Boards (IRB) rely on the Biomedical Research Alliance of New York (BRANY) as the single IRB for regulatory review and oversight. The protocol was approved by BRANY (IRB File # 23-02-622 and registered at ClinicalTrials.gov (NCT06076304) prior to the initiation of enrollment.

Informed consent is initiated prior to starting any research procedures and is a continuous process throughout the study; an extensive discussion of risks and possible benefits of the interventions will be provided by trained research personnel either in person or remotely, and written consent must be documented via electronic or paper signature.

### Participants

The Nasal Steroids, Irrigation, Oral Antibiotics, and Subgroup Targeting for Effective Management of Acute Sinusitis (NOSES) study is designed to enroll individuals with a higher likelihood of bacterial ARS, based on IDSA guidelines [14]. Eligibility criteria exclude patients with conditions, complications, comorbidities, or immune impairments that would preclude safe study participation, including in a placebo arm. Detailed eligibility criteria are provided in Table 1. They are based on participant self-report or clinician’s assessment completed as a part of usual clinical care.

**Table 1:** Inclusion and Exclusion Criteria.

Inclusion criteria:
1. 18–75 years old; **AND** experiencing either #2 or #3:
2. “persistent” symptoms or signs (see below) compatible with ARS or sinus infection lasting for 1–21 days without evidence of clinical improvement;
Symptoms: facial pain or pressure, facial congestion or fullness, nasal obstruction, nasal discharge, no or reduced sense of smell, headache, bad-smelling breath, fatigue, ear pain or pressure, dental pain;
**OR**
3. Worsening symptoms or signs characterized by the new-onset of fever, headache, or increase in nasal discharge following a typical viral upper respiratory infection (URI), with symptoms lasting 5–6 days and initially improving (“double-sickening”).
**Exclusion criteria:**
1. allergy or intolerance to penicillin;
2. systemic antibiotic therapy in the past 4 weeks;
3. prior sinus surgery;
4. complications of rhinosinusitis (facial edema or swelling, cellulitis), or orbital, meningeal or cerebral signs;
5. clinician determined need for antibiotics or hospital admission;
6. pregnancy or breastfeeding;
7. presence of a comorbidity or medication that may impair a patient’s immune response, as determined by a treating clinician;
8. hospitalization in past 5 days;
9. unable or unwilling to provide informed consent or comply with study protocol requirements;
10. fever >39°C or 102°F “today”;
11. taking intranasal corticosteroids regularly in the past two weeks and unwilling to stop for 3 days;
12. previously enrolled or participated in the NOSES study.

### Recruitment and screening procedures

Study participants will be recruited from clinics affiliated with six academic sites across U.S.: the University of Wisconsin–Madison (Madison, Wisconsin), Virginia Commonwealth University (Richmond, Virginia), University of California–Los Angeles (Los Angeles, California), Washington State University (Seattle, Washington), Penn State (Hershey, Pennsylvania), and Georgetown University (Washington, DC). Sites were selected based on investigator expertise, prior success conducting clinical trials, and geographic diversity. All sites participated in a feasibility study prior to the launch of the full-scale study and demonstrated readiness through successful recruitment and protocol implementation [37].

For in-clinic enrollment, study sites will collaborate with clinicians in primary care and urgent care settings who care for patients with ARS. Clinics and clinicians serving as primary recruitment sites were selected based on site-level patient volume, infrastructure, resources, and interest in participation, as documented during the feasibility assessment.

To reflect real-world care pathways for ARS, recruitment will also occur via online portals where local investigators will evaluate patients for eligibility; this will occur outside of clinic settings.

Interested individuals will be contacted by, or will themselves contact, site research personnel who will explain the study and conduct eligibility screening. Enrollment procedures—including informed consent, collection of clinical and demographic information, and distribution of saline nasal irrigation (SNI) educational materials and kits—may occur in a private location convenient for the participant, such as the clinic, residence, community, or an academic research space. In addition, participants can be screened remotely and will complete study surveys either remotely or in person; however, they will need to meet a research coordinator in-person to collect a finger stick sample for CRP analysis.

After the study enrollment and baseline assessments (surveys, CRP assay), participant activities will follow one of three pathways; 1. Participants with symptom duration of less than 9 days at enrollment will enter Phase 1 and receive supportive care recommendations, an SNI kit, and instructions on its use. 2. Participants with symptom duration of at least 9 days at enrollment or those enrolled into Phase 1 whose symptoms reached day 9 and have not improved will be randomized into one of the four study arms (Phase 2). 3. Participants experiencing “double-sickening” may bypass the symptom duration criterion and be directly enrolled into Phase 2 with randomization into one of the four study arms. The duration of the Phase 1 (supportive care) period was selected based on evidence suggesting that patients with at least 9 days of ARS symptoms are more likely to have bacterial ARS [14,38]. Participants whose symptoms persist for at least 9 days will be offered to continue to Phase 2, with the decision about this progression made by the individual.

### Study design

The protocol was developed by academic partners at six primary care research centers—Georgetown University, Pennsylvania State University, the University of California–Los Angeles, the University of Washington, the University of Wisconsin–Madison, and Virginia Commonwealth University—drawing on their collective expertise in clinical trials and the evidence base ARS. We also conducted focus groups with clinicians and staff at the pretrial stage. Additionally, we sought feedback from all the committees, including the Patient Advisory Board (PAB) about different aspects of the trial. This approach reflects the growing emphasis on engagement during the pretrial phase of randomized controlled trials, allowing investigators to identify potential recruitment challenges, gain deeper insight into real-world clinical settings and patient populations, and enhance the transferability of study findings.

This is a multicenter, pragmatic, placebo-controlled RCT. Study participants will be randomized in a 1:1:1:1 ratio to four treatment groups with allocation concealment. Based on a sample size calculation, the study is powered to detect clinically meaningful within and between group changes in outcomes of interest. The study has two phases:

**Phase 1 – Post Enrollment (E)** Participants presenting early in the course of their ARS (i.e., days 1–9 of ARS) will receive supportive care and watchful waiting. Information on symptomatic care options and SNI kits will be distributed. Phase 1 can last between 1 and 9 days (Days E1-E9) for individual participants.
**Phase 2 – Post Randomization (R)** Participants from Phase 1 or new enrollees who are symptomatic on day 9 or later, or display double-sickening (worsening symptoms after an initial improvement) will be randomized to one of four treatment groups and followed for 14 days. Phase 2 can last between 1 and 14 days (Days R1-R14) for individual participants.

All participants, regardless of phase or study group, will receive instructions for supportive care, which includes as-needed over-the-counter (OTC) acetaminophen, guaifenesin, dextromethorphan, pseudoephedrine, and short-term use of oxymetazoline spray; participants can purchase these OTC medications by themselves, if they wish. Additionally, the treating clinician can recommend any medication with the exception of antibiotics or INCS. An SNI kit will be offered to all participants. The kit will include a Soft Tip Micro-Filtered Nasal Wash System with pre-packaged salt packets.

**Fig 1:**
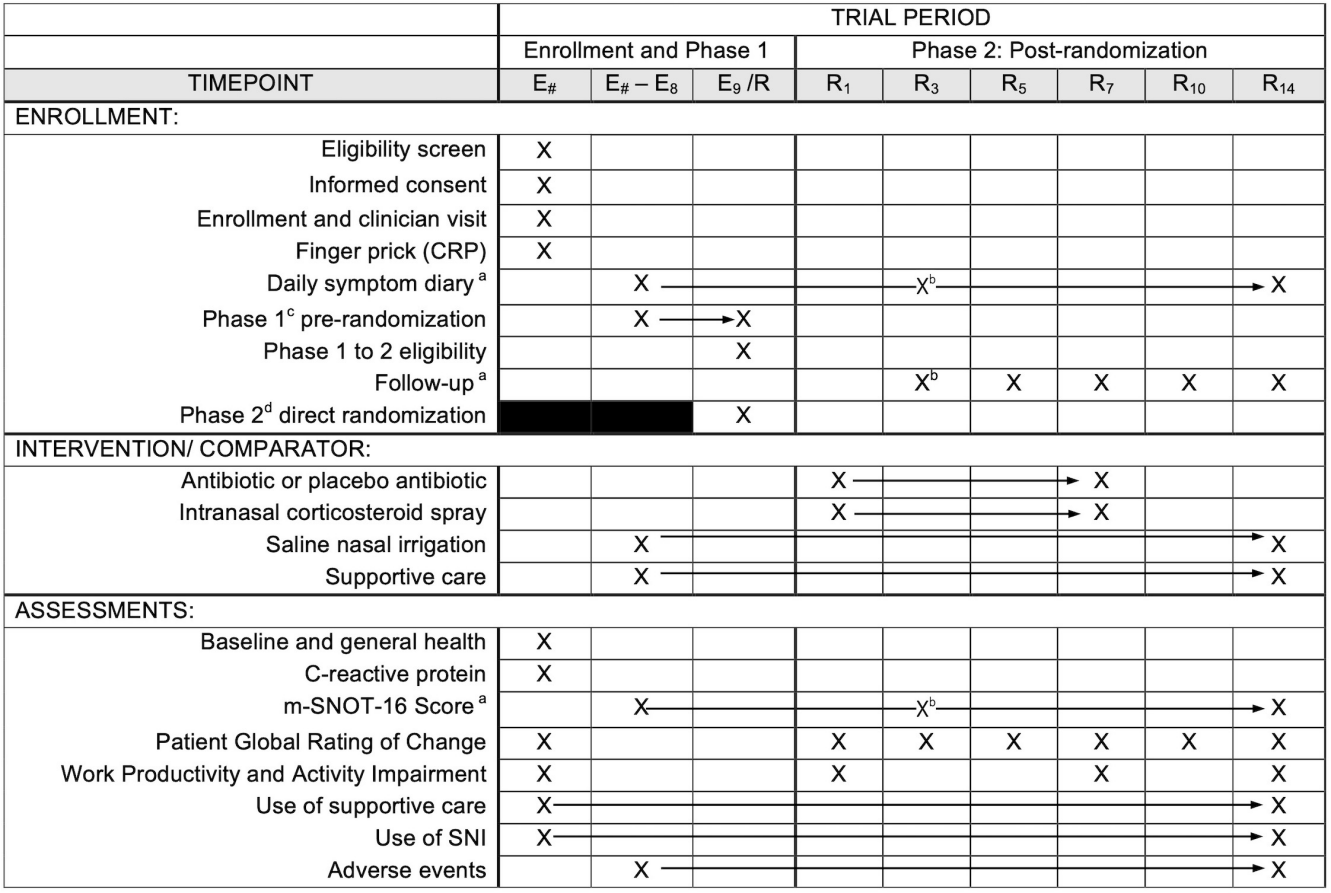
Participant timeline: Schedule of enrollment, interventions, and assessments. ^a^ Daily symptom diary and follow-up survey track the following: mSNOT-16, Patient Global Rating of Change, Work Productivity and Activity Impairment Questionnaire, use of interventions and supportive care, and adverse events. ^b^ Primary outcome: mSNOT-16 score at Day R3. ^c^ Patients with less than 10 days of symptoms at enrollment enter Phase 1 (pre-randomization period) until Day E9. Phase 1 to 2 eligibility is assessed at Day E9. ^d^ Patients with ≥ 10 days of symptoms or double sickening enter Phase 2 and randomization directly. ^e^ 2025 Standard Protocol Items: Recommendations for Interventional Trials (SPIRIT), Chan A-W, Boutron I, Hopewell S, Moher D, Schulz KF, et al. SPIRIT 2025 statement: updated guideline for protocols of randomised trials. BMJ 2025;389:e081477. https://dx.doi.org/10.1136/bmj-2024-081477.

A timeline of study events is presented in Fig 1. The study will enroll 3,720 participants into Phase 1, with 2,232 (60%) estimated to be randomized into Phase 2 to achieve the following aims:

1. Assess the comparative effectiveness of oral antibiotics (amoxicillin-clavulanate) versus INCS in ARS patients who do not improve with supportive care.
2. Identify patient subgroups that benefit most from oral antibiotics versus INCS.
3. Identify patient subgroups who improve with supportive care alone (Phase 1) and do not progress into Phase 2 of the study.

### Randomization and blinding

Randomization will use the REDCap web-based system with a permuted block scheme by site, four bin numbers per site, and random block sizes of 8. Participants will be automatically assigned to their allocated group. All research personnel and participants will be blinded to treatment assignments, except for INCS administration.

Treating clinicians and research personnel will remain blinded. If treatment failure occurs, primary care clinicians may manage patients without breaking the blind. Clinicians may modify or stop interventions as clinically indicated and may submit requests for unblinding through a Blinding Specialist unaffiliated with the research team.

### Assessments and outcome measures

Data will be collected using the REDCap platform. At the initial visit, research staff and/or participants will complete pre-screening, eligibility, baseline health, demographic, and other enrollment forms. Participants will also complete a daily symptom survey. On Day E1, a fingerstick blood sample will be collected for CRP testing. Point-of-care testing (POCT) will be performed by research personnel using a QuikRead go Instrument and CRP (Aidian, Espoo, Finland).

### Primary and secondary outcomes

Primary and secondary outcomes, along with other measures of interest, are summarized in Table 2. The primary outcome is disease-specific quality of life at Day R3, measured using the patient-oriented Modified Sino-Nasal Outcome Test (mSNOT-16) [39]. The mSNOT-16, validated for primary care ARS patients, was adapted from the Rhinosinusitis Outcome Measure-31 [40], which was developed with patient input to capture outcomes meaningful to them.

**Table 2:** Data Collection/Outcome Measures.

Primary outcome measure	Description	Measurement time frame
Symptom improvement	Improvement of symptoms will be assessed using the Modified Sino-Nasal Outcome Test (mSNOT-16), a disease-specific quality of life questionnaire. Sixteen sinus symptoms are self-assessed on a 0–3 point scale, with 0 = No Problem, 1 = Mild or Slight Problem, 2 = Moderate Problem, 3 = Severe Problem. The mean of the total score is used to assess symptom severity. Daily measures will be collected both in Phase 1 and Phase 2.	Change from Day 1 to Day 3 in Phase 2; differences in longitudinal trends across groups during Phase 2
**Secondary outcome measures**	**Description**	**Measurement time frame**
Percent improved beyond minimal clinically important difference	Percent of patients who improved more than 0.5 on mSNOT-16	Phase 1: Baseline to Day 9; Phase 2: Day 1 to Day 3
Patient non-randomization rate	Percentage of patients who were enrolled in Phase 1 but did not proceed to randomization because they reported their condition had improved. This is determined by the patient at the Day 9 assessment, by decreased mSNOT-16 scores from baseline, or no longer reports symptoms listed in the inclusion criteria.	Phase 1: baseline to Day 9
Supportive care	Types and frequency of supportive care used.	Phase 1: Baseline to Day 9; Phase 1: Days 1, 3, 5, 7, 10, 14
Work Productivity and Activity Impairment Questionnaire	Work and activity impairment due to acute sinusitis; the Work Productivity and Activity Impairment Questionnaire: Specific Health Problem 2.0 is a 6-question self-reported questionnaire on the effects of sinus symptoms on the amount of absenteeism (percent work time missed), presenteeism (percent impairment while working), overall work impairment, and daily activity impairment. Higher percentages indicate greater impairment and less productivity (scale 0–100%).	Phase 1: Baseline, Day 9 day; Phase 2: Days 1, 7, and 14
Global Rating of Improvement as Quality of Life	Self-assessment of current sinus symptoms at each follow-up interview using a 6-point categorical scale (1 = no symptoms, 2 = a lot better, 3 = a little better, 4 = the same, 5 = a little worse, or 6 = a lot worse).	Phase 1: Baseline, Day 9; Phase 2: Days 1, 7, and 14
Symptomatic care	Use patterns of over-the-counter medicines or supplements.	Phase 1: daily; Phase 2: daily
Adverse events	Adverse events reported during a follow-up or on the survey. Events are graded form 1–5 using the NCI Common Terminology Criteria for Adverse Events.	Phase 1: daily; Phase 2: daily
Adherence	Self-reported adherence to study pill and nasal spray are calculated by [number of doses taken]/[prescribed number of doses] x 100, over the 7-day intervention period.	Phase 2: Days 1–7
Prevalence of double-sickening	Worsening of symptoms after an initial improvement	Direct randomization to Phase 2
**Other measures of interest**	**Description**	**Measurement time frame**
Saline nasal irrigation	SNI use on 50% or more of study days; saline concentration, number of times used per day, timing of day	Phase 1: daily; Phase 2; daily
C-reactive protein (CRP)	Measurement of C-reactive protein (CRP) level as a predictor of bacterial infection, of not improving in Phase 1 and proceeding to Phase 2, and of poorer outcomes in non-antibiotic groups	Phase 1 and Phase 2
Clinician’s Estimation of the Likelihood of Bacterial Infection and/or Benefit From Antibiotics	Low, intermediate, or high probability that this patient has bacterial sinusitis and will benefit from antibiotics	Enrollment Day
Seasonal/Geographical Fluctuations in Symptom Severity	Difference in baseline mSNOT-16s score across sites and seasons	Enrollment Day
Change in mSNOT-16 scores from symptom start day to 14 days post-randomization	Longitudinal differences between daily mSNOT-16 scores from symptom start day, baseline and and visit days: Day 1–9 before randomization to Days 1–14 post-randomization. A decrease in mSNOT-16 score would indicate an improvement in symptoms.	Symptom start day to 14 days post-randomization

The mSNOT-16 includes 16 sinus-specific symptom items rated on a 4-point scale: 0 = no problem, 1 = mild/slight, 2 = moderate, 3 = severe. Respondents may also identify up to five symptoms most important to their health. The individual mSNOT-16 score is the mean of the 16 items, ranging from 0 to 3. The minimally important clinical difference is 0.50, representing a small change on the self-reported Patient Global Rating of Change scale (“a lot worse” to “a lot better”) [39,41].

The daily symptom survey records mSNOT-16 scores and supplemental medication use, with participants receiving daily reminders. The Work Productivity and Activity Impairment (WPAI) Questionnaire will assess absenteeism, presenteeism, and activity impairment due to ARS over the preceding 7 days [42]. Clinicians will also provide their clinical prediction of bacterial sinusitis probability (low, intermediate, high).

### Treatment groups

Participants who did not improve in Phase I or those who had more than 9 days of symptoms at enrollment or those with double-sickening will be randomized to one of the four treatment groups in Phase 2: antibiotics alone, antibiotics plus INCS, placebo antibiotics plus INCS and placebo antibiotics alone. Table 3 summarizes the interventions to be conducted in four groups.

**Table 3:** Treatment Groups and Interventions.

Participant group	Intervention
Antibiotic	· Amoxicillin/clavulanate, oral, 875 mg/125 mg twice daily for 7 days
Placebo antibiotic	· Placebo for amoxicillin/clavulanate, oral, twice daily for 7 days
Antibiotic + intranasal corticosteroid (INCS)	· Amoxicillin/clavulanate, oral, 875 mg/125 mg twice daily for 7 days · Budesonide nasal spray, 32 mcg per spray, 2 sprays per nostril, once per day
Placebo antibiotic + intranasal corticosteroid (INCS)	· Placebo for amoxicillin/clavulanate, oral, twice daily for 7 days · Budesonide nasal spray, 32 mcg per spray, 2 sprays per nostril, once per day

### Safety considerations

Safety will be overseen by the single IRB, Principal investigators (PIs), and an independent Data Safety Monitoring Board (DSMB). Adverse Events (AEs) will be graded using the NCI Common Terminology Criteria for Adverse Events (CTCAE) Version 5.0. Severe or medically significant AEs resulting in hospitalization or death will be considered Serious Adverse Events (SAEs). Expedited reporting requires serious, unanticipated, and related events to be reported to the single IRB within 5 days, and to the DSMB Chair within 48 hours. The DSMB will review safety data after 10%, 20%, 40%, and 70% data completion.

Individuals who are actively pregnant and breastfeeding are not eligible to enroll in this trial. Participants who become pregnant while on study will be recommended to withdraw from the intervention arms.

### Data management

The Data Coordinating Center (MedStar Health Research Institute) will manage data collection and quality. Data quality checks will be embedded into REDCap, preventing entry of data outside acceptable ranges and requiring answers for necessary items. Participant data will be stored on secure servers and protected by two-factor authentication. Identifiable information will be maintained separately from the research database.

### Statistical analysis plan

Descriptive statistics will summarize demographic and clinical characteristics of study participants for the overall enrolled sample (Phase 1) and the randomized sample (Phase 2). Continuous variables will be described using means, standard deviations, medians, and ranges, while categorical variables will be summarized using frequencies and percentages.

Given the four-group study design, bivariate comparisons of continuous outcomes will be conducted using analysis of variance (ANOVA), with Bonferroni-adjusted pairwise comparisons to control for multiple testing (α = 0.05/6 = 0.008). Categorical variables will be compared using Chi-square tests or Fisher’s exact tests, as appropriate. Within-group changes will be assessed using paired t-tests for continuous outcomes and symmetry tests for paired proportions.

The primary outcome analysis (Aim 1) will be conducted using both the intent-to-treat (ITT) and per-protocol approaches. The primary outcome is the change in modified SNOT-16 (mSNOT-16) score from randomization visit R1 to follow-up visit R3. This outcome will be used to evaluate the comparative effectiveness of the four intervention groups.

Although six pairwise comparisons are possible, the primary comparisons of interest are:

1. **Effect of antibiotics:** Antibiotics only vs placebo antibiotics
2. **Effect of intranasal corticosteroids:** Placebo antibiotics + INCS vs placebo antibiotics
3. **Added benefit of INCS among antibiotic users:** Antibiotics only vs antibiotics + INCS
4. **Comparison of antibiotics vs INCS:** Antibiotics only vs placebo antibiotics + INCS

An overall ANOVA will first test for differences in change in mSNOT-16 across the four groups. If the F-test is statistically significant, pairwise comparisons will be conducted using Bonferroni-adjusted Type I error. For pairwise comparisons that are not statistically different, equivalence testing will be performed using 99% confidence intervals and a predefined equivalence margin of ±0.25 (half of the clinically meaningful important difference). If equivalence is rejected, non-inferiority or superiority will be assessed; otherwise, results will be considered inconclusive.

To account for potential baseline confounding and to leverage longitudinal data, multivariable analyses will be conducted using linear mixed-effects models. These models will include fixed effects for treatment group and relevant baseline covariates (age, sex, CRP level, and double-sickening) and random effects at both the study site and participant levels.

Heterogeneity of treatment effects (Aim 2) will be examined using linear regression models of change in mSNOT-16 that include interaction terms between treatment group and the subgroup variable of interest, adjusting for baseline mSNOT-16. Subgroup variables to be evaluated include elevated CRP, double-sickening, purulence on examination, change in smell, dental pain, provider clinical impression/gestalt, and visit type (in-person vs telehealth). Due to sample size limitations, interaction terms will be tested one at a time.

For Aim 3, analyses will focus on identifying factors associated with clinical improvement during Phase 1 without progression to Phase 2. Logistic regression models will be used to examine demographic and clinical predictors hypothesized to be related to bacterial infection and reduced likelihood of improvement with supportive care alone. Additional exploratory analyses will assess symptom clusters derived using machine-learning–based clustering methods. All statistical analyses will be conducted using Stata 19 [43] and R [44].

### Sample size

This study is a parallel, four-group randomized clinical trial designed for comparative effectiveness. The primary outcome is the mSNOT-16 score, with effectiveness assessed as the mean change from baseline to Day 3.

Based on the Garbutt et al. validation study (baseline mean mSNOT-16 = 1.71, mean change = 0.58, SD = 0.52, MCID = 0.50), initial sample size calculations aimed to estimate the mean change in each group with high precision (95% CI, margin of error ±0.05) [39]. Calculations indicated that 2,232 participants need to be randomized (1:1:1:1; accounting for 20% dropout), with 3,720 participants expected to consent and enroll, and assuming 60% of enrolled participants would not improve spontaneously and would require randomization.

The sample size was further refined based on pilot data to support subgroup analyses and ensure sufficient power for equivalence and superiority testing. Observed mSNOT-16 changes across the four groups (0.30, 0.40, 0.42, 0.29) with a maximum SD of 0.60 were used to compute power for a 4 × 3 factorial design. Assuming subgroup means of 0.30, 0.40, and 0.50 (SD = 0.60), a total of 1,860 participants provides: 1) 80% power at α = 0.005 (Bonferroni adjustment for 10 pairwise comparisons) to detect at least one significant difference among the four treatment groups (effect size = 0.10); 2) 99% power to detect at least one significant pair within the subgroup variable (effect size = 0.14); 3) 100% power to detect a significant interaction (effect size = 0.17) with 155 participants per cell for 12 treatment combinations (4 × 3).

Additionally, 465 participants per group provide 100% power for equivalence testing for each of the six possible pairwise comparisons across four groups, using a Bonferroni-adjusted α = 0.05/6 = 0.008 and a margin of equivalence of 0.25 (half the MCID). If a difference between groups is not statistically significant (p > 0.008), the two one-sided test (TOST) procedure will be applied. Equivalence will be concluded if the 99% confidence interval for the difference lies within [−0.25, 0.25]. If equivalence cannot be shown or a significant difference exists, the same margin will be used to assess non-inferiority or superiority, comparing the lower or upper bound of the 99% CI to −0.25 or 0.25, respectively. Sample size calculations were conducted in PASS [45].

### Status and timeline

The full-scale study commenced with the first enrollment on January 29, 2025, following completion of an 18-month feasibility and pilot (n = 140) phase [37]. Participant recruitment is estimated to run from the first quarter of 2025 (Q1) through Q1 2028, and data collection will be completed by Q2 of 2028. Data analysis of the specific aims will be completed in Q4 2028. The total planned duration for the full-scale study is 48 months.

## Conclusion

This study should be a pivotal trial that transforms care for ARS. ARS is highly prevalent but remains understudied, with only a few small randomized trials reporting heterogeneous results. Our trial, nearly ten times larger than any prior ARS study, will allow robust identification of patient subgroups that benefit from specific therapies, evaluation of multiple supportive care strategies, and more precise guidance on appropriate antibiotic use. Beyond advancing care for a common primary care condition, we hope this study will encourage further rigorous investigation of primary care issues with significant public health impact.

## Supporting information

S1 Fig Schematic of multicenter, pragmatic, randomized controlled clinical trial with patients presenting with symptoms of acute sinusitis (ARS). Based on the number of symptomatic days, participants either: start Phase 1, a supportive care and waiting period; or directly enter Phase 2 and are randomly assigned to one of four intervention groups comparing antibiotics to placebo, with or without an intranasal corticosteroid.
(TIF)

S1 File SPIRIT 2025. Checklist of items to address in a randomized trial protocol*.
(DOCX)

S2 File Protocol. Approved NOSES protocol.
(PDF)
