# Supplementary material for: Nasal steroids, irrigation, oral antibiotics, and subgroup targeting for effective management of acute sinusitis (NOSES): Protocol for a comparative effectiveness randomized controlled trial
Source: PLoS One. 2026 May 6;21(5):e0348522. doi: 10.1371/journal.pone.0348522 (PMC13148658; doi:10.1371/journal.pone.0348522)
Supplement: S2 File — Approved NOSES protocol. (PDF) [file pone.0348522.s003.pdf]

PROTOCOL

NASAL STEROIDS, IRRIGATION, ORAL ANTIBIOTICS, AND SUBGROUP TARGETING FOR EFFECTIVE MANAGEMENT OF SINUSITIS

Project Phase:	**Full-Scale Study**	
Principal Investigators:	Clinical Coordinating Center:<br>Daniel Merenstein, MD<br>djm23@georgetown.edu<br>Georgetown University	Data Coordinating Center:<br>Nawar Shara, PhD<br>Nawar.Shara@medstar.net<br>MedStar Health Research Institute
Co-Investigators/Site Leads:	David Rabago, MD<br>drabago@pennstatehealth.psu.edu<br>Penn State College of Medicine<br><br>Derjung Mimi Tarn, MD, PhD<br>dtarn@mednet.ucla.edu<br>University of California Los Angeles<br><br>Bruce Barrett, MD, PhD<br>Bruce.Barrett@fammed.wisc.edu<br>University of Wisconsin-Madison	Alex Krist, MD<br>alexander.krist@vcuhealth.org<br>Virginia Commonwealth University<br><br>Sebastian Tong, MD<br>setong@uw.edu<br>University of Washington
Funder:	Patient-Centered Outcomes Research Institute (PCORI)	
Protocol Number:	PLACER-2021C3-24476	
IRB Number:	BRANY IRB File # 23-02-622-Master File	
ClinicalTrials.gov ID:	NCT06076304	
Version Number:	2.1	
Version Date:	October 15, 2025	
IRB Approval:	BRANY October 30, 2025	

STATEMENT OF COMPLIANCE

The study will be carried out in accordance with the protocol and by the following:

- United States (US) Code of Federal Regulations (CFR) applicable to clinical studies (45 CFR Part 46; 21 CFR Part 50, 21 CFR Part 56, and 21 CFR Part 312, and/or 21 CFR Part 812)
- International Conference on Harmonisation Good Clinical Practice (ICH GCP) E6(R2)

All key personnel and staff who are responsible for the design, conduct, management, or oversight of the study have completed Human Subjects Protection and ICH GCP Training.

SIGNATURE PAGE

The signature below constitutes the approval of this protocol and the attachments, and provides the necessary assurances that this trial will be conducted according to all stipulations of the protocol, including all statements regarding confidentiality, and according to local legal and regulatory requirements and applicable US federal regulations and International Conference on Harmonization guidelines.

Site Investigator: Daniel Merenstein, MD	
Signed:	Date:
Daniel Merenstein, MD<br>Principal Investigator	

PROTOCOL AMENDMENT HISTORY

Version	Date	Section	Summary of Changes	Rationale
0.1	7/1/23	Global		Initial draft
1.0	8/15/23	Global	Correction of typographical and administrative errors. Update of background and rationale and clarification of text to align with the approved research plan.	Revisions to draft
1.1	1/15/24	Global<br>4.2.6, 8.3.2<br>6.2<br>8.2<br>9.7.2<br>13.4<br>14.6	Correction of typos and administrative errors<br>Addition of nasal swab procedures<br>Updated product storage procedures<br>Addition of pre-screening procedure<br>Out of range CRP value<br>Addition of remote informed consent process<br>Updated protocol deviation procedures	Revisions to draft
2.0	12/5/24	Global<br><br><br><br>Summary, 4.1, 5, 5.1<br>Summary, 4.2, 5.1, 7.1<br>4.1<br><br>4.2.2<br>4.2.3<br><br>6.4<br>8.1<br><br><br>8.3.2<br>9.3<br>10.1<br><br>Appendix A<br>Appendix B<br><br>Appendix F<br>Appendix G	Approved feasibility phase protocol v1.1 with changes for the full-scale study.<br>Correction of typographical, formatting, and other administrative errors.<br>Increased inclusion age from 65 to 75 y.<br>Addition of double sickening to inclusion crit.<br>Expanded description of Phases 1 and 2. Added qualifying conditions for double sickening.<br>Changed neti pot to nasal wash system.<br>Clarified conditions for patients to progress to Phase 2.<br>Added detailed directions for supportive care.<br>Removed proposed procedures for Recruitment Outside the Clinic Setting. Forms list moved to Appendix G.<br>Removed optional nasal swab collection (tbd).<br>Refined stopping criteria and DSMB plan.<br>Refinements and additional details added for clarity to the statistical analysis plan.<br>Updated full-scale study timeline.<br>Updated handout with care instructions for nasal wash system.<br>Revised ICF to reflect full-scale study changes.<br>Updated data collection forms.	Post-approval of research plan from funder
2.1	10/15/25	5.3.2, 8.1<br>Appendix G	Added procedures for Outside of Clinic Enrollment.<br>Updated data collection forms.	

TABLE OF CONTENTS

STATEMENT OF COMPLIANCE 2
SIGNATURE PAGE 3
PROTOCOL AMENDMENT HISTORY 4
TABLE OF CONTENTS 5
LIST OF ABBREVIATIONS 9
PROTOCOL SUMMARY 11
Figure 1. Patient Flow Diagram 13
1 KEY ROLES 14
1.1 Site Coordination 15
Study Implementation and Recruiting Sites 15
1.2 Committees and Meeting Schedules 16
1.2.1 Core Research Committee (CRC) 17
1.2.2 Participant Advisory Board (PAB) 17
1.2.3 Study Advisory Committee (SAC) 18
1.2.4 Dissemination and Engagement Committee (DEC) 18
1.3 Site Governance Plan 18
1.3.1 Process for making decisions on scientific direction 18
1.3.2 Communication plan 19
1.3.3 Procedure for resolving conflicts 19
1.3.4 DCC Governance and Roles 19
1.3.5 Contingency plan 20
1.3.6 Authorship and publications 20

2 BACKGROUND INFORMATION AND SCIENTIFIC RATIONALE 22
2.1 Background Information 22
2.2 Rationale 22
2.2.1 Impact and overuse of antibiotics due to imprecise treatment 22
2.2.2 Gaps in evidence - issues in diagnosing ARS 23
2.2.3 C-reactive protein (CRP) testing 23
2.2.4 Limited treatment options and patient centeredness 23
2.2.5 Evidence for intranasal corticosteroids (INCS) 24
2.2.6 Evidence and usage of saline nasal irrigation 24
2.2.7 Impact of telehealth 24

3 OBJECTIVES 25
3.1 Study Objectives and Hypotheses 25
3.2 Study Outcome Measures 25
3.2.1 Primary outcome measure 25
3.2.2 Secondary outcome measures 26
3.2.3 Subgroups 26
3.3 Potential Risks and Benefits 27
3.3.1 Potential risks 27
3.3.2 Known potential benefits 27
3.3.3 Importance of knowledge to be gained 28

4 STUDY DESIGN 29

4.1 Full-Scale Study 29
4.1.1 Impact of feasibility activities
4.2 Interventions and Laboratory Measures 29
4.2.1 Supportive care 29
4.2.2 Saline nasal irrigation 29
4.2.3 Transition to Phase 2 or withdrawal 30
4.2.4 Randomization 31
4.2.5 CRP testing 31

5 STUDY ENROLLMENT AND WITHDRAWAL 32

5.1 Inclusion/Exclusion Criteria 32
5.2 Intervention Assignment Procedures 33
5.2.1 Randomization procedures 33
5.2.2 Masking procedures 33
5.2.3 Reasons for withdrawal 33
5.2.4 Handling of withdrawals 34
5.2.5 Termination of study 34
5.3 Recruitment and Retention Strategy 34
5.3.1 Recruitment 34
5.3.2 Outside of Clinic Enrollment 36
5.3.3 Retention 37
5.4 Payment for Participation 37

6 STUDY INTERVENTIONS 38

6.1 Study Products 38
6.1.1 Acquisition and preparation 38
6.1.2 Packaging and labeling 38
6.2 Product Storage, Administration, and Accountability Procedures 38
6.3 Assessment of Compliance with Study Products 38
6.4 Supportive Care and Concomitants 39
6.5 Saline Nasal Irrigation 40

7 STUDY SCHEDULE 41

7.1 Phase Determination 41
7.1.1 Double sickening 41

8 STUDY PROCEDURES/EVALUATIONS 43

8.1 Clinical Evaluations 43
8.2 Pre-screening 43
8.2.1 Pre-screening versus research procedures 43
8.2.2 Information collected 43
8.2.3 Consent for pre-screening activities 44
8.2.4 Verbal consent script 44
8.3 Laboratory Evaluations 45

9 ASSESSMENT OF SAFETY 46

9.1 Definition of Adverse Event 46
9.2 Assessing Adverse Events 46
9.2.1 Grading 46
9.2.2 Relationship to study participation 46
9.2.3 Expectedness 46
9.2.4 Serious adverse events 47
9.3 Study Halting Rules/Stopping Criteria 47
9.4 Withdrawal of Individuals 47
9.5 Reporting Procedures 47
9.5.1 Reportable events 47
9.5.2 Other adverse events 48
9.5.3 Reporting of pregnancy 48
9.6 Safety Oversight 48
9.6.1 DSMB plan for pilot study 48
9.6.2 DSMB plan for full-scale study 49
9.7 Safety and Blinding 49
9.7.1 Breaking blind 50
9.7.2 Out of range CRP value 50

10 STATISTICAL CONSIDERATIONS 51

10.1 Sample Size Considerations For Full-Scale Study 51
10.2 Final Analysis Plan 52
10.2.1 Secondary outcomes 53
10.2.2 Analysis of other outcomes 54
10.2.3 Sensitivity analysis to determine the impact of key assumptions 54

11 SOURCE DOCUMENTATION 56

11.1 Study Records 57

12 STUDY STARTUP AND SITE MONITORING 58

12.1 Startup Plan 58
12.1.1 Required training for engaged research personnel 58
12.1.2 Manual of procedures and forms 59
12.1.3 Institutional review board (IRB) 59
12.1.4 Registration of trial 59
12.1.5 Site initiation visit 59
12.2 Data Coordinating Center (DCC) Functions 59
12.2.1 Data quality assurance 59
12.2.2 Data collection approaches 60
12.2.3 Web-based data collection 60
12.2.4 Tracking dashboard 60
12.2.5 Data reporting 60
12.2.6 Handling of metadata 61
12.2.7 Network data security 61
12.3 Clinical site monitoring 61

12.3.1 Monitoring 61
12.3.2 Data audits 62

13 ETHICS/PROTECTION OF HUMAN SUBJECTS 63

13.1 Ethical Standard 63
13.2 Institutional Review Board 63
13.3 Informed Consent Process 63
13.4 Remote Informed Consent 63
13.5 Exclusion of Women, Minorities, and Children (Special Populations) 64
13.6 Patient Confidentiality 65
13.7 Study Discontinuation 65

14 DATA HANDLING AND RECORD KEEPING 66

14.1 Data Management Responsibilities 66
14.2 Data Capture Methods 66
14.3 Types of Data 66
14.4 Timing/Reports 66
14.4.1 Quality control (QC) reports 66
14.4.2 Performance reports 67
14.5 Study Records Retention 67
14.6 Protocol Deviations 67

15 DATA SHARING 69

15.1 Funding Acknowledgement and Disclaimer 69
15.2 Return of Research Results to Study Patients 69

16 REFERENCES 70

17 APPENDICES 75

APPENDIX A: Project Timeline 76
APPENDIX B: Saline Nasal Irrigation Instructions 77
APPENDIX C. Modified Sino-Nasal Outcome Test - 16 and Instructions for Scoring 83
APPENDIX D: Committee Charters 85
APPENDIX E: Data Safety Monitoring Board Charter 87
APPENDIX F: Informed Consent Form (Masterfile Template) 88

LIST OF ABBREVIATIONS

ACORN	Virginia Ambulatory Care Outcomes Research Network
AE	Adverse Event/Adverse Experience
ARS	Acute Rhinosinusitis
BRANY	Biomedical Research Alliance of New York
CAPRICORN	Capital Area Primary Care Research Network
CDC	Centers for Disease Control and Prevention
CFR	Code of Federal Regulations
CRC	Core Research Committee
CRF	Case Report Form
CRP	C-reactive Protein
CTCAE	Common Terminology Criteria for Adverse Events
DCC	Data Coordinating Center
DEC	Dissemination and Engagement Committee
DSMB	Data and Safety Monitoring Board
GCP	Good Clinical Practice
GU	Georgetown University
HIPAA	Health Insurance Portability and Accountability Act
ICF	Informed Consent Form
ICH	International Conference on Harmonisation
ID	Identifier
IDSA	Infectious Diseases Society of America
IRB	Institutional Review Board
MID	Minimally Important Difference
INCS	Intranasal Corticosteroids
MHRI	MedStar Health Research Institute
MOP	Manual of Procedures
mSNOT-16	Modified Sino-Nasal Outcome Test-16
N	Number (of subjects)
NCI	National Cancer Institute, NIH
NIH	National Institutes of Health
PAB	Patient Advisory Board
PCORI	Patient-Centered Outcomes Research Institute
PCRN	UCLA Primary Care Research Network
PBRN	Practice-Based Research Network
PI	Principal Investigator
PLACER	Phased Large Awards for Comparative Effectiveness Research
PSARN	Penn State Ambulatory Research Network
RCT	Randomized Controlled Trial

PSCOM	Penn State College of Medicine
REDCap	Research Electronic Data Capture
SAC	Serious Adverse Event/Serious Adverse Experience
SAE	Study Advisory Committee
SNI	Saline Nasal Irrigation
UCLA	University of California Los Angeles
US	United States
WWAMI	Washington, Wyoming, Alaska, Montana and Idaho
WPRN	WWAMI Practice and Research Network
WHO	World Health Organization
VCU	Virginia Commonwealth University

PROTOCOL SUMMARY

**Full Title:**	Nasal steroids, irrigation, oral antibiotics and subgroup targeting for effective management of acute sinusitis
**Short Title:**	NOSES
**Project Phase:**	**Full-Scale**
**Sample Size:**	Full-Scale Study: 3,720 patients<br>Pilot (Feasibility): 140 patients *Completed*
**Population:**	Between the ages of 18 and 75 who meet the clinical criteria for acute rhinosinusitis (ARS)
**Recruitment Sites:**	Georgetown University/Capital Area Primary Care Research Network (CAPRICORN)<br>PSCOM/Penn State Ambulatory Research Network (PSARN)<br>University of California Los Angeles/UCLA Primary Care Research Network (PCRN)<br>University of Washington/WWAMI region Practice and Research Network (WPRN)<br>University of Wisconsin-Madison (UWM)<br>Virginia Commonwealth University/Virginia Ambulatory Care Outcomes Research Network (ACORN)
**Study Duration:**	Full-Scale Study: 48 months<br>Feasibility: 18 months *Completed*
**Active Duration:**	Phase 1 (supportive care) from 1-9 days<br>Phase 2 (randomization) 14 days<br>Patients re-enrolled for double sickening maximum 30 days
**Interventions:**	Phase 1 (supportive care)<br>Saline nasal irrigation (SNI)<br>Recommendations for supportive care<br><br>Phase 2 (randomization)<br>Amoxicillin-clavulanate 875 mg/125 mg or placebo PO bid for 7 days<br>Intranasal corticosteroids (INCS) bid for 7 days<br>Saline nasal irrigation (SNI)<br>Recommendations for supportive care
**Design:**	Randomized, placebo-controlled trial with four treatment groups<br>Group 1: Antibiotics<br>Group 2: Placebo antibiotics + intranasal corticosteroids<br>Group 3: Antibiotics + intranasal corticosteroids<br>Group 4: Placebo antibiotics
**Specific Aims:**	AIM 1: To compare, through patient-reported outcomes, the efficacy of oral antibiotics (amoxicillin-clavulanate), and INCS, for clinical improvement of ARS among patients who do not improve with supportive care alone. We expect that approximately 60% of patients will not improve with supportive care. Hypothesis:

	Mean SNOT scores at day 3 following randomization will differ among the four groups.<br><br>AIM 2: To identify which patient subgroups benefit most from oral antibiotics (amoxicillin-clavulanate) versus INCS. Hypothesis: Potential predictors of heterogeneity of treatment effects include baseline patient and disease characteristics, and higher C-reactive protein (CRP) levels that are more consistent with bacterial infection. Also patients enrolled via telehealth will be less sick and thus not respond as well to antibiotics.<br><br>AIM 3: To identify which patient subgroups improve with supportive care, and do not require antibiotics or INCS. Consistent with pragmatic clinical practice, patients with symptom duration up to 10 days will be provided with a checklist of, and recommendations for, standard-of-care, over-the-counter supportive treatment options; their utilization will be tracked. Hypothesis: A subgroup of patients with self-directed SNI use will experience symptomatic and quality of life improvement by day 10 of their illness.

Figure 1. Patient Flow Diagram

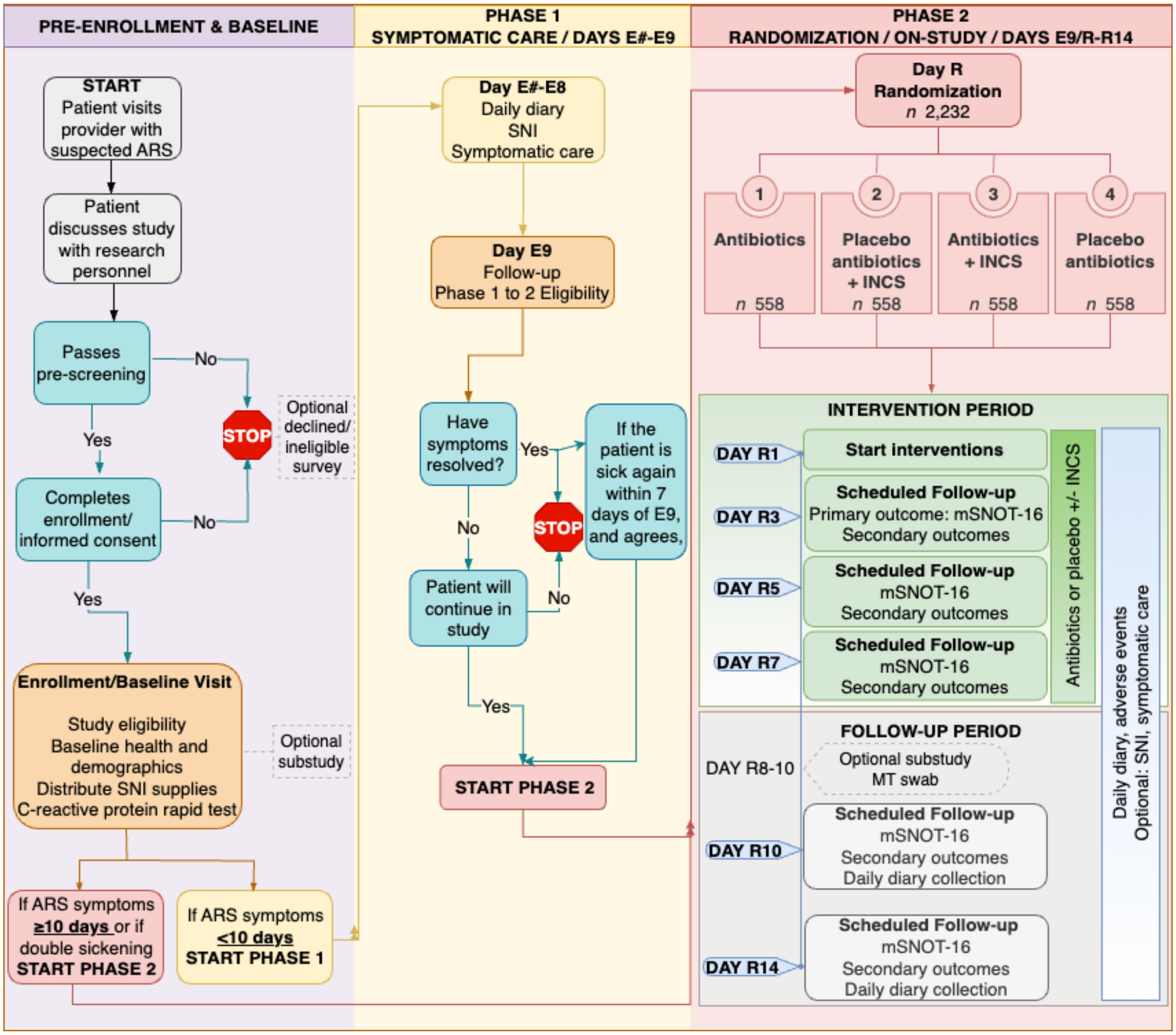

1 KEY ROLES

Clinical Coordinating Center (CCC)
Principal Investigator:
Daniel Merenstein, MD
djm23@georgetown.edu
Georgetown University
Department of Family Medicine

Data Coordinating Center (DCC)
Principal Investigator:
Nawar Shara, PhD
nawar.shara@medstar.net
MedStar Health Research Institute
Department of Biostatistics and Bioinformatics

Co-Investigators/Site Leads:

Bruce Barrett, MD, PhD
Bruce.Barrett@fammed.wisc.edu
University of Wisconsin-Madison
Department of Family Medicine & Community Health

Alex Krist, MD MPH
alexander.krist@vcuhealth.org
Virginia Commonwealth University
Department of Family Medicine and Epidemiology

David Rabago, MD
drabago@pennstatehealth.psu.edu
Penn State College of Medicine
Department of Family and Community Medicine

Derjung Mimi Tarn, MD PhD
dtarn@mednet.ucla.edu
David Geffen School of Medicine at UCLA
Department of Family Medicine

Sebastian Tong, MD, MPH
setong@uw.edu
University of Washington
Department of Family Medicine

1.1 Site Coordination

Study Implementation and Recruiting Sites

Georgetown University/Capital Area Primary Care Research Network (CAPRICORN)
Director: Adam Visconti, MD

PSCOM/Penn State Ambulatory Research Network (PSARN)
Director: Aleksandra Zgierska, MD

University of California Los Angeles/UCLA Primary Care Research Network (PCRN)
Director: Derjung Mimi Tarn, MD, PhD, Daniel T. Lee, MD

University of Washington/WWAMI region Practice and Research Network (WPRN)
Director: Sebastian Tong, MD, MPH

University of Wisconsin-Madison
Director: Bruce Barrett, MD, PhD

Virginia Commonwealth University/Virginia Ambulatory Care Outcomes Research Network (ACORN)
Director: Jacqueline Britz, MD, MSPH

1.2 Committees and Meeting Schedules

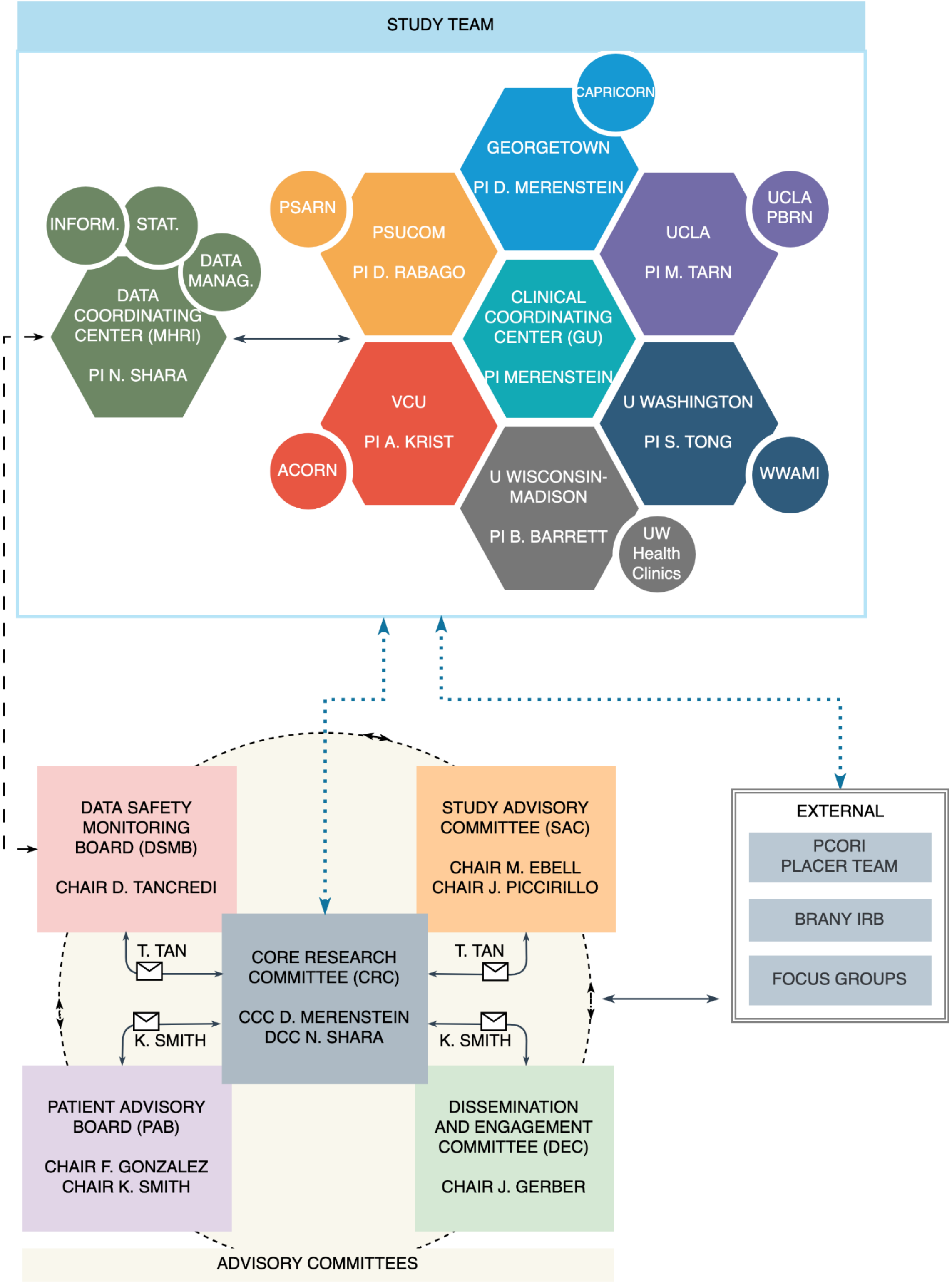

1.2.1 Core Research Committee (CRC)

The CRC consists of Dr. Shara, Dr. Merenstein, site PIs, site project coordinators, Ms. Herbin-Smith, Ms. Tan and two Patient Advisory Board (PAB) members. The PI, Dr. Merenstein, will lead the bi-monthly team meetings. The team meetings will occur via web/teleconference, except the first meeting. The team will develop and discuss strategies related to daily operations, maintenance and organizational alignment, as needed. The meetings are intended to address daily activities and functioning of the project. Any research member may request agenda items for inclusion.

Team meeting agendas vary by phase of the trial, questions being asked by research personnel and issues raised by PCORI or other committees. During years 5-6 the meetings will concentrate on preparing manuscripts and working with partners, such as the Centers for Disease Control and Prevention (CDC), Societies and Academies, in implementation of any clinical practice changes.

1.2.2 Participant Advisory Board (PAB)

The 12-member PAB-two representatives from each study site- includes patients who have had at least one episode of Acute Rhinosinusitis (ARS) in the past three years. They are a diverse group of individuals from each of the 6 sites and are representative of our patient populations in age, ethnicity, race, gender, locality, and socioeconomic status.

Ms. Gay Thomas, owner of GR Thomas Advisors LLC, will help lead the formation, training, and ongoing interactions with the PAB. Ms. Thomas is a co-founder of the Wisconsin Network for Research Support. Ms. Thomas assists in developing a tailored study orientation for our PAB members. She also provides ongoing consultation to support effective PAB meeting preparation, retention of PAB members and advise on meeting documentation. Based on the consultation of Ms. Thomas, the PAB will have two facilitators: Project Coordinator, Ms. Keisha Herbin Smith, will lead administrative duties including note keeping, follow-up planning, scheduling, etc. Ms. Florencia Gonzalez, from Howard University, will be the Lead Facilitator, a position she holds for a similar PAB for the Georgetown-Howard Universities Center for Clinical and Translational Science (supported by the National Institutes of Health (NIH) Clinical and Translational Science Awards Program). We have budgeted for her expertise and time spent on preparing for and moderating each meeting.

PAB members will be encouraged to participate in other committees and will be additionally compensated for their participation. Each PAB member will receive a $200 stipend per meeting, and if there is any travel, a $250 travel stipend per day plus reimbursement of travel expenses. It is important that PAB members remain involved throughout the 5.5 years of the study. The PAB will meet 3-6 times per year. If travel conditions allow, one in-person meeting may be planned.

The responsibilities for the PAB evolve as the study progresses. Initially, during the feasibility phase the PAB reviewed recruitment plans, patient expectations, and study materials. As the study progressed, they spent time on the website. Ms. Thomas and Ms. Gonzalez will continue to work with the PAB on timely input and provide feedback to the CRC about all aspects of the study.

1.2.3 **Study Advisory Committee (SAC)**

The SAC will provide oversight, resolve conflicts, and provide direction as needed by any committee. The SAC will meet two-four times per year during the full-scale study phase as indicated. Committee chairs will present to the SAC at every meeting and may convene ad-hoc meetings as needed. SAC Co-Chairs will be Dr. Jay Piccirillo, former Vice-Chair of the American Academy of Otolaryngology–Head and Neck Surgery and co-author of their Clinical Practice Guideline: Adult Sinusitis Update, and Dr. Mark Ebell, an internationally respected expert in critically evaluating literature and in primary care respiratory infections. SAC members are ear, nose and throat, infectious disease and primary care physicians, ARS researchers, stakeholders and government employees.

1.2.4 **Dissemination and Engagement Committee (DEC)**

The DEC is tasked with: (i) creating an ongoing dissemination and translation strategy, (ii) engaging multiple stakeholders in dissemination activities, and (iii) assessing the readiness of the findings to be disseminated. The DEC will meet 2-4 times per year during the full-scale study phase as indicated. The DEC is chaired by Dr. Jeff Gerber, Medical Director of the Antimicrobial Stewardship Program at Children's Hospital of Philadelphia, and recipient of PCORI funding for the project, Comparative Effectiveness of Broad- versus Narrow-Spectrum Antibiotics for Acute Respiratory Tract Infections in Children. The DEC includes Drs. Merenstein and Rabago, representing the GU and PSUCOM sites, two PBRN directors and two project coordinators, representing the other four sites, Dr. Nawar Shara, representing the DCC, other researchers with expertise in social media, communication, community engagement, stakeholders and government employees.

1.3 Site Governance Plan

The Clinical Coordinating Center (CCC) is run by Dr. Merenstein and the Data Coordinating Center (DCC) by Dr. Shara. Although they have a long record of close collaboration, both the CCC and DCC will be autonomous and independent of each other. Dr. Merenstein will be in charge of recruitment, retention, and all clinical aspects of the trial. Dr. Shara will oversee the development of the data management protocols, trial database, data collection, and data collection tools, applicable training, and coordinate the preparation of the trial reports.

1.3.1 **Process for making decisions on scientific direction**

The PI with the relevant expertise in clinical or statistical research will make the scientific decisions related to his/her field. Each recruitment site will have a site PI, project coordinator, and research assistants. Additionally, Dr. Merenstein will oversee a study project coordinator (Keisha Herbin-Smith) and project manager (Tina Tan), based at the Georgetown site. Ms. Herbin-Smith will be responsible for all patient issues such as recruitment, enrollment, retention, follow-up, sample collections, etc. Ms. Tan will be responsible for non-clinical and operational issues not related to data, such as regulatory requirements, shipping of supplies, budget management, etc. Dr. Shara will oversee a data manager and

head statistician who will be responsible for all study data, including analysis for committees as needed, statistical work, etc.

1.3.2 Communication plan

The PIs will communicate at least weekly. During the full-scale study phase, the two PIs, data manager, head statistician, project coordinator and project manager will meet in-person or via Zoom every other week.

Dr. Merenstein will be primarily responsible for all aspects of the grant involving communication with PCORI (as contact PI). Responsibility for proper use of grant funds, regulatory compliance, and reporting will remain the responsibility of both PIs with the assistance of the project manager.

1.3.3 Procedure for resolving conflicts

This is a large study that will take input from expert site PIs, multiple committees including the PAB, clinical office staff, research personnel, among others. However, all final significant scientific and policy decisions will be made by the PIs. The PIs have collaborated extensively and have been jointly funded on two previous trials without any significant conflicts. Drs. Shara and Merenstein are both well respected in their respective fields and have worked with many collaborators without needing conflict resolution. However, in the event that a consensus cannot be reached, formal requests will be submitted to Drs. Ebell and Piccirillo, scientific consultants on this study and co-chairs of the SAC, who will mediate scientific conflicts. The PIs agree to abide by the final recommendations and decisions made by either Drs. Ebell and Piccirillo, or the SAC, should Drs. Ebell and Piccirillo decide to raise the issue to the full committee.

1.3.4 DCC Governance and Roles

The DCC consists of multidisciplinary investigators, led by Dr. Shara who believes in a consensus-driven process that is collaborative, cooperative and egalitarian. The DCC staff is a diverse and well-trained group in statistical methods, data management and data processing that positions the DCC to successfully execute this clinical trial.

The DCC team, under the guidance of Dr. Shara, will be responsible for creating and housing datasets which will be gathered from all sites. All study and intervention protocols and materials as described will be made available to PCORI directly, or at PCORI's request, may be made publicly available.

The DCC will securely transfer the data and its associated metadata (i.e., data dictionaries, file formats), as needed, to the researcher(s) using a secure file transfer protocol methodology which will be determined with the respective IT teams. The DCC will set and maintain the proper permissions that allow only authorized users to access their specific information. A data request intake and fulfillment process will be utilized. The DCC will collect from requesters: the type of request, purpose and scope of

use, period of data, desired variables, and deadline needed. This process will allow for efficient dissemination and quality control. Comprehensive documentation will be developed, which describes the study along with codes for each data variable collected and any programming code or instruction for coding to create variables. To create the de-identified dataset, the final study database used in conducting the final analysis will be used. All protected health information and any other patient identifiers, including the study-assigned patient identifier (ID), will be removed and replaced by a unique code available only to the Biostatistics team.

Further protections for data reporting will include the following: All dates will be converted to time periods, for example baseline, day 1, day 3 and so forth. Age will be provided in wide age-groups (e.g. 5-year increments), and truncated at the top and bottom, depending on the age distribution. Race/ethnic groups will be provided only in wide-groupings. Clinics will be removed and denoted only by a number within each site. Only research data will be included in the released dataset, including data from intervention visits, patient- provided health assessments, and study-obtained medical record and laboratory data. Non-research data, including tracking forms, non-study health information, and any patient identifiers are not included. Similarly, serious adverse event (SAE) data that is collected for safety but not research purposes will not be included in the data release. Specific details of de-identification will be discussed with the study group to further ensure patient and, if required, clinic confidentiality.

1.3.5 Contingency plan

If a PI moves to a new institution, attempts will be made to transfer the relevant portion of the project to the new institution. In the event that a PI cannot carry out his/her duties or the project cannot be transferred, the participating institutions will identify a replacement PI. If a site PI moves to a new institution, the initial attempt will be made to keep the project at their original institution and appoint a replacement site PI. The study PIs Merenstein and Shara will work with the local site PIs and committee chairs to replace departing research personnel or committee members and to ensure all members are participating in committee meetings. If an agreement cannot be reached by the CRC on a new appointment, the external Data Safety and Monitoring Board (DSMB) will make the final decision on appointments.

1.3.6 Authorship and publications

A full NOSES Collaborative Authorship Guidelines has been developed and reviewed by all sites. This is a very large trial with an overarching goal of transforming care provided in primary care settings that will likely result in numerous papers. The Authorship Guidelines specifies most requirements, but the CRC will be responsible for addressing the important issues of authorship as needed.

In addition to formal publications, a one-page review of study findings, free of medical jargon and written in lay language and at an acceptable grade level for patients (generally agreed to be at fifth grade level) will be prepared and given to all patients upon study completion. Once the data are

finalized, patients will receive the one-page review. With input from the PAB and SAC, the DCC will work on a format and delivery method for sharing results. Additionally, participating clinicians and clinics will receive a summary of the results (overall and per clinic), and we will work with individual offices to produce further materials for dissemination in their office, such as pamphlets or posters, if study outcomes are applicable for such dissemination tools. The DEC, PAB and SAC will also plan dissemination events specifically for patients, clinicians, and other stakeholders, as well as joint meetings. One such event will include a study summit at the conclusion of the study. Other activities will include social media, presentations/town halls/grand rounds at community organizations and clinics, webinar or other training materials, and other print or online resources for all stakeholders.

2 BACKGROUND INFORMATION AND SCIENTIFIC RATIONALE

2.1 Background Information

Acute rhinosinusitis (ARS) is so prevalent that it affects 15% of the adult population annually, and accounts for 20% of all antibiotics prescribed to adults in the outpatient setting.[1–8] Antibiotics have historically been prescribed about 90% of the time for outpatient, physician-diagnosed, ARS. More recent data from the CDC shows some improvement but still over 70% of the time antibiotics are prescribed for ARS.[3,4,9–11] However, most patients do not benefit from antibiotics as their symptoms are caused by a viral infection. According to a review in *Rhinology*, "The usage of antibiotics in ARS is widespread and there seems to be only a slight added benefit in the usage of antibiotics over placebo in the treatment of ARS. Hence, larger scale studies should be done in the future to confirm the results of these studies."[12]

The majority of cases of ARS resolve without antibiotics; however, antibiotics do benefit some patients. Previous research suggests the following attributes will likely predict response to antibiotic treatment; individuals with: (a) an elevated CRP level, (b) double-sickening, defined as, "Have you had worsening of sinus symptoms after initial improvement," (c) evidence of purulence on clinical examination, (d) unpleasant odor different from normal (cacosmia), (e) pain in the teeth, (f) provider clinical impression/gestalt.[13–19] These subgroups are suggestive of bacterial infection; however, even the subgroups that are often regarded as good predictors of antibiotic benefit, such as double-sickening, only have a positive likelihood ratio of 1.30 (0.77-2.40).[17] Our goal is to identify which subgroups would more likely benefit from different commonly used treatments -- antibiotics, intranasal corticosteroids, saline nasal irrigation, supportive care, and/or watchful waiting – and improve management options and outcomes for patients with ARS.

2.2 Rationale

2.2.1 Impact and overuse of antibiotics due to imprecise treatment

Global overuse of antibiotics increases antibiotic resistance and unnecessary medication-related adverse events.[20] The COVID-19 pandemic has underscored the criticality of proper diagnosis and treatment of respiratory infections in primary care; unfortunately, ARS is one of the most poorly treated outpatient respiratory infections. In the U.S., one in seven adults (30 million office visits) are diagnosed with ARS every year, resulting in one in five antibiotic prescriptions, and accounting for over $11 billion in direct annual costs.[1–5,7,21] Because ARS is so prevalent and the impact of inappropriate antibiotic use is widespread, the populations and health decisions affected by this research impacts almost all individuals. Reducing inappropriate prescribing for ARS is crucial to limiting the development and spread of antibiotic resistance.[22] The World Health Organization has identified the overuse of antibiotics and subsequent resistance as a top public health concern, while the United Nations convened a high-level meeting to coordinate approaches to address the root causes of antimicrobial resistance[23] - the fourth health issue to ever be addressed by the General Assembly.[24] Many stakeholders have prioritized reduced antibiotic use and alternative treatment options for ARS, including the Centers for Disease Control and Prevention (CDC) Get Smart: Know When Antibiotics Work program, which identifies ARS as

the number one reason for inappropriate outpatient antibiotic use.[25] Additionally, the Infectious Disease Society of America (IDSA), the American Academy of Family Physicians (AAFP) and Choosing Wisely® have all called for decreased usage of antibiotics for ARS.

Inappropriate use of antibiotics is not only a societal issue, but an individual issue as well. Our research group and other researchers have shown significant microbiome and metabolic changes that may have long term individual health impact.[26–30] Per the CDC, antibiotics are one of the most common reasons for emergency department visits for adverse drug events, with antibiotics being responsible for 16% of all adverse drug reactions.[31]

2.2.2 Gaps in evidence - issues in diagnosing ARS

Unlike other infections in primary care (e.g. pharyngitis, COVID-19, urinary tract infections, or pneumonia - for which clinical and/or laboratory tools are available), routine lab tests and physical exam findings cannot distinguish viral from bacterial ARS.[1,13,32–34] We conducted a meta-analysis of nine randomized trials in patients with clinically diagnosed ARS and could not determine whether using common signs and symptoms could help identify patient subgroups that benefit from antibiotic treatment.[35–43] We did confirm a moderate, albeit not statistically significant, effect of antibiotics on cure (number needed to treat (NNT)=15).[13] A Cochrane review co-authored by PI Merenstein found a NNT with antibiotics of 19.[1] The review concluded that there is no place for antibiotics for people with uncomplicated acute rhinosinusitis. It also recommended further research to help differentiate patients who may benefit from antibiotics versus those who may not, based on symptoms and point-of-care testing. The review relied on a heterogeneity of small studies; the sample size of this current proposal is nearly as large as all previous studies combined (the review had 15 trials involving 3,057 patients).

2.2.3 C-reactive protein (CRP) testing

The European Position Paper on Rhinosinusitis and Nasal Polyps group also recommends such research, with a focus on assessment of biomarkers such as C-reactive protein (CRP) to help predict who may respond to an antibiotic and clinical markers that can predict bacterial ARS or a clinically important response to antibiotics.[44] While some studies have shown some benefit from antibiotics and others have shown no success, it remains difficult to determine characteristics of patients who derive benefit.[14,40,45–48] Thus, a large, pragmatic RCT is needed to identify clinical and laboratory indicators that predict which subgroups may benefit from antibiotic versus non-antibiotic interventions. Importantly, we will use patient-developed and oriented outcomes to ascertain effectiveness. Existing literature is also limited by a focus on confirmation of ARS by diagnostic tests that are unavailable in primary care settings (such as, computed tomography scan, ultrasound, and sinus puncture).[15,38,49–53] Besides being limited by availability, some are also limited by test characteristics; for example ultrasound is good for ruling out ARS but has very poor specificity.[16]

2.2.4 Limited treatment options and patient centeredness

Antibiotics are widely prescribed for ARS, despite previous primary care studies which demonstrated a limited role for antibiotics on a per-patient basis.[39,54] A comprehensive review from 2020 concluded,

"Despite being commonly prescribed, the evidence supporting the routine use of oral antibiotics for the majority of ARS is lacking."[55] However, previous research also demonstrated that a subgroup of patients with ARS benefit from antibiotics.

2.2.5 Evidence for intranasal corticosteroids (INCS)

Intranasal corticosteroids (INCS) are the cornerstone of treatment for chronic rhinosinusitis and have thus generated interest and usage for ARS, where they have been shown to provide relief in a subset of patients.[40] A Cochrane review and meta-analysis[56] by Thompson et al. of 1,943 patients found that patients receiving INCS were more likely to experience symptom resolution or improvement than those receiving placebo.[57] A double blinded RCT performed in Europe demonstrated that a subset of patients responded to INCS, but due to the small sample size the authors were unable to characterize these patients; our large sample size will allow us to clarify INCS responsiveness.[40] INCS is an excellent comparator due to both wide usage and evidence showing symptomatic relief in a subset of patients; the European Position Paper on Rhinosinusitis and Nasal Polyps group also recommends INCS.[40,58]

2.2.6 Evidence and usage of saline nasal irrigation

Saline nasal irrigation (SNI) is a promising adjunctive treatment modality that is recommended for a variety of sinonasal conditions by several national groups. SNI is recommended as a treatment for ARS by AAFP, AAO-HNS guidelines, and IDSA.[45,59–61] Robust evidence supports the use of SNI, using concentrations of 0.9% to 2% saline, for chronic sinus symptoms but less so in ARS. In four clinical chronic rhinosinusitis studies, three of which Co-Investigator Rabago led or was involved with, patients (N=1,104) using SNI reported improved sinus symptoms and decreased medicalization of sinus conditions, including decreased use of sinus-related medication.[62–65] SNI appears in the IDSA treatment guidelines as adjunctive treatment for ARS[61] and clinicians recommend SNI for ARS.[66] While SNI has become nearly standard of care for ARS and holds promise as a means to improve care in ARS, it has not been formally tested in a rigorous effectiveness trial.

2.2.7 Impact of telehealth

Telehealth is a relatively new practice modality that has become widely used in primary care practices to evaluate respiratory infections but little is known about the details of use,[67,68] including core aspects such as rationale (for example, convenience), and how telehealth consultations impact primary care provider prescription of antibiotics for ARS symptoms.[69,70]

3 OBJECTIVES

3.1 Study Objectives and Hypotheses

<u>AIM 1</u>**: To compare, through patient-reported outcomes, the efficacy of oral antibiotics (amoxicillin-clavulanate), and INCS, for clinical improvement of ARS among patients who do not improve with supportive care alone.** We expect that approximately 60% of patients will not improve with supportive care. *<u>Hypothesis</u>*: Mean SNOT scores at day 3 following randomization will differ among the four groups.
<u>AIM 2</u>**: To identify which patient subgroups benefit most from oral antibiotics (amoxicillin-clavulanate) versus INCS.** *<u>Hypothesis</u>**:*** Potential predictors of heterogeneity of treatment effects include baseline patient and disease characteristics, and higher C-reactive protein (CRP) levels that are more consistent with bacterial infection. Also, patients enrolled via telehealth will be less sick and thus not respond as well to antibiotics.
<u>AIM 3</u>**: To identify which patient subgroups improve with supportive care, and do not require antibiotics or INCS.** Consistent with pragmatic clinical practice, patients with symptom duration of less than 10 days will be provided with a checklist of, and recommendations for, standard-of-care, over-the-counter supportive treatment options; their utilization will be tracked. *<u>Hypothesis</u>:* A subgroup of patients with self-directed SNI use will experience symptomatic and quality of life improvement by day 10 of their illness.

3.2 Study Outcome Measures

3.2.1 Primary outcome measure

The primary outcome, patient improvement, will be the patient's disease-specific quality of life score at Day R3, as measured by the patient-oriented mSNOT-16 (***Appendix C***)[71] which has been validated at Day R3 for primary care patients with ARS. The mSNOT-16 was one of the sinonasal outcome tests developed from the Rhinosinusitis Outcome Measure-31 and validated for primary care patients with ARS.[71] The Rhinosinusitis Outcome Measure-31 was developed with patient focus groups to reflect what they feel are meaningful outcomes.[72] In addition to the 16 items with which patients rank the severity of their sinus-related symptoms, mSNOT-16 gives patients the option to identify up to five of the most important items affecting their health.[73] Each of the 16 sinus-specific quality of life symptoms is recorded on a four-point scale; responses are either 0=no problem; 1=mild or slight problem; 2=moderate problem; and 3=severe problem. The recorded patient's individual mSNOT-16 score is the mean of the 16 responses, and this value ranges from 1 to 3. As stated in the Garbutt et al. paper, "to aid in the interpretation of study results that show a change in the SNOT-16 score, we calculated the "minimally important difference" (MID), the smallest difference in score that is clinically significant. We defined this value as the change in SNOT-16 score for those who reported a small change in their symptoms on the global rating scale. Using the patient's global assessment of change in symptoms from baseline, we categorized "a lot worse" and "a lot better" and "no symptoms" as a large change, "a little

worse" and "a little better" as a small change, and "the same" as no change."

3.2.2 Secondary outcome measures

As per **Table 1**, secondary outcomes include: illness duration measured using a symptom diary, treatment failure, re-consultation for the same symptoms, missed work, etc. We will also examine if provider clinical impression/gestalt helps predict clinical improvement. Baseline characteristics will be assessed on the day of enrollment, referred to in this proposal as day E. After enrollment, the days will be referred to as days E#-E9 depending on the length of time the patient has had symptoms. On the ninth day of symptoms patients will be randomized, referred to in this proposal as day E9. For example, a patient who initially presents on their 6th day of symptoms (day E6) will be in phase 1 of the study for 4 days. The day they start the intervention in phase 2 is referred to as day R1. Patients that present on or after their ninth day of symptoms will be enrolled and randomized on the same day. These patients will skip phase 1 of the study and proceed to day R1 of the phase 2 interventions. A patient's overall health improvement will be assessed by the Patient Global Rating of Change scale[74] and their missed work by the Work Productivity and Activity Impairment Questionnaire.

Table 1. Primary and Secondary Outcomes

Primary or Secondary	Name of outcome	Specific measure to be used	Timepoints	Estimated power
Primary	Resolution of symptoms	mSNOT-16	Day R3	90%
Secondary	Resolution of symptoms	mSNOT-16	Days E-E9, R(1,5,7,10,14)	90%
Secondary	Complete daily symptom diary	Daily diary	Days E--E9, R(1-14)	N/A
Secondary	Supportive care before Day 1	What supportive care was used and associated costs	Days E,*E#-E9	N/A
Secondary	Activity assessment	Work Productivity and Activity Impairment Questionnaire	Days E, R(1,7,14)	N/A
Secondary	Adverse events	Follow-up calls and daily diary	Varied	N/A
Secondary	Clinically relevant outcomes	Clinical characteristics	Days E, R(1,3,5,7,10,14)	N/A
Secondary	Supportive care after randomization	What supportive care was used and associated costs	Days R(1,3,5,7,10,14)	N/A
Secondary	Quality of life	Patient Global Rating of Change	Days E, R(1,3,5,7,10,14)	N/A
Exploratory	Watchful waiting	Never reached R1 because improved	Days E(1-9)*	N/A
Exploratory	SNI usage	Concentration, days, times per day	Days E(1-9)*	N/A

*Patients will have varying amounts of symptomatic days during Phase 1 depending on how many days of symptoms they had prior to enrollment.

3.2.3 Subgroups

Previous research suggests the following attributes will likely predict response to antibiotic treatment; individuals with: (a) an elevated CRP level, (b) double-sickening, defined as, "Have you had worsening of sinus symptoms after initial improvement," (c) evidence of purulence on clinical examination, (d) unpleasant odor different from normal (cacosmia), (e) pain in the teeth, (f) provider clinical impression/gestalt. [13–19] These subgroups are suggestive of bacterial infection; however, even the

subgroups that are often regarded as good predictors of antibiotic benefit, such as double-sickening, only have a positive likelihood ratio of 1.30 (0.77-2.40).[17] We will also examine the subgroup of telehealth enrollees.

3.3 Potential Risks and Benefits

3.3.1 Potential risks

Initially patients will use supportive care, including SNI. This is consistent with standard of care and guidelines. There is a risk their ARS symptoms progress and they feel sicker. After Phase 1 patients will be randomized to one of four study groups: Group 1: Antibiotics; Group 2: Placebo antibiotics + Intranasal corticosteroids; Group 3: Antibiotics + Intranasal corticosteroids; Group 4: Placebo antibiotics.

It will be explained to patients that they may receive a placebo (no active medicine). Two of the four groups will receive an active antibiotic (amoxicillin-clavulanate) and the other two groups will receive a placebo antibiotic. Additionally, two of the four groups will receive INCS. The risks involved with taking a placebo without an active are that the symptoms may not improve or get worse. We will inform the patients that at any time during the study, they can follow-up with their health care clinician if their symptoms are not improving.

We will be using an approved medication that has known potential risks. The most common risks and side effects related to the treatments we are studying include:

- For amoxicillin-clavulanate: nausea and/or vomiting, diarrhea, upset stomach and mild skin rash.
- INCS: nasal irritation, nosebleeds, headaches.
- For saline nasal irrigation: nasal irritation, nasal stinging and nosebleeds.

Risks and side effects related to the capillary blood draw (fingerstick) are considered to be minimal. The risks associated with having capillary blood sampling are exceedingly rare but may include:

- Excessive bleeding
- Fainting or feeling light-headed
- Infection (a slight risk any time the skin is broken)

It will also be explained to the patients that there may also be side effects other than those listed that we cannot predict. Many side effects go away shortly after the treatments are stopped, but in some cases side effects can be serious, long lasting or permanent. Patients will be informed to discuss any questions about potential risks with the researchers and their health care clinician.

3.3.2 Known potential benefits

The potential benefits to the patients of this study may include: symptomatic resolution due to nasal irrigation, intranasal corticosteroids, and/or antibiotic treatments; avoidance of potential side effects in patients who did not receive antibiotic treatment; and close monitoring of symptoms with the follow-up interviews in all treatment groups. Patients may or may not directly benefit from their participation, but have an opportunity to benefit others by increasing knowledge about sinusitis and sinusitis treatments.

The risks of participation in this study are reasonable and are the same risks as could occur in the usual care of patients with acute sinusitis. The benefits of this study, both for the individual patient and society, outweigh the potential risks.

3.3.3 Importance of knowledge to be gained

The results of this study can lead to innovative and practical ways to evaluate and treat sinusitis. The study may also lead to a reduction in the inappropriate use of antibiotics. This could result in reduced costs, reduced exposure to the risks of treatment, and reduction in selection pressures for resistant bacteria in the community. The outcome of the reduction in the unnecessary use of antibiotics will therefore be the improved care of patients in the primary care setting.

4 STUDY DESIGN

The project consists of two distinct stages: a feasibility phase (including pilot trial) and the full-scale study. The methods and procedures of the pilot were almost identical to the planned full-scale trial, with a smaller sample size (3,720 for full study, 144 for pilot). Pilot recruitment finished on March 3, 2024 with 140 patients enrolled.

4.1 Full-Scale Study

The goal of this project is to improve outcomes for patients with acute rhinosinusitis (ARS) by understanding for whom antibiotics, supportive care, watchful waiting or intranasal corticosteroids (INCS) are most likely to provide benefit. Patients between 18 and 75 years of age who meet the clinical criteria for ARS will be recruited from primary care and urgent care centers in six regions across the U.S. Once enrolled, patients report on their ARS symptoms via a daily diary which include disease-specific quality-of-life assessments and patient-reported outcomes (see Section 3.2).

Patients start the study in one of two phases according to the number of days they are experiencing ARS symptoms:
Phase 1: Patients with ARS symptoms for 9 or less days at the time of enrollment will start a pre-randomization period of supportive care which lasts until the patient reaches their 9th day of symptoms. At the 9th day:
If their symptoms have not improved, the patient will enter Phase 2.
If their symptoms improve or they no longer wish to continue, the patient does not enter Phase 2 and their study participation concludes*.
Phase 2: Patients with symptoms for ≥10 days at the time of enrollment visit bypass Phase 1 and enter directly into Phase 2, randomize, and start the interventions.
*Phase 1 patients who improved and concluded the study at day 9 may re-enter the study, if they qualify for double sickening within 7 days (see Section 7.1).

4.2 Interventions and Laboratory Measures

4.2.1 Supportive care

A standardized approach to symptomatic care and instructions will be provided to all patients. All patients will be given detailed instructions (see **Section 6.4**) and inform research personnel about their usage.

4.2.2 Saline nasal irrigation

During the enrollment visit, SNI use will be discussed with all patients. All research personnel will be educated on how to instruct on usage of SNI. Patients who are interested in using SNI will be instructed to begin with a 0.9% solution (one pre-packaged salt packet). Patients who experience no discomfort will be advised to adjust upward to 2.0% (2 pre-packaged salt packets) per 8 ounces; volume indicators are marked on the SNI bottle). Patients will be instructed to begin with two irrigations per day, and

adjust to 1 or 3 daily if desired, at whatever times of the day are appropriate for them. All patients will be provided an over-the-counter product called a "Soft Tip Micro-Filtered Nasal Wash System" (SinuCleanse®, provided by Ascent Consumer Products). This product incorporates attributes of the traditional "neti pot" (easy to hold, gravity based pressure) with elements of a squeeze bottle device (adjustable water pressure, familiar bottle appearance). We have selected this SNI system because it has a self-contained filter that allows patients with municipal water systems to use tap water rather than boiled or distilled water. It meets CDC recommendations for nasal irrigation with municipal tap water. Patients who have water delivered to their tap via a well will be provided bottled distilled water by research personnel. Both neti pot and squeeze bottle are equally satisfactory to patients. The flexibility of SNI use parameters is patient-oriented (timing, saline concentration, frequency). It allows patients to control the therapy within evidence-based, safe parameters to accommodate the personal characteristics, conditions, and preferences of patients. Prior studies suggest that the ability to choose concentration, frequency, and timing of irrigations are the most important correlates of successful SNI.[62,75,76] Patients will mix saline solution on a per-use basis using tap or distilled water and pre-packaged over-the-counter salt packets (SinuCleanse®, provided by Ascent Consumer Products). The study team will provide patients with the necessary SNI materials: instructions, Soft Tip Micro-Filtered Nasal Wash System, salt packets, and distilled water for patients with well water systems.

4.2.3 Transition to Phase 2 or withdrawal

Primary care often uses delay prescribing, in order to reduce antibiotic usage. A meta-analysis of 55,682 patients, from nine randomized controlled trials and four observational studies found that with exception of young children, delayed antibiotic prescribing is safe and effective even for those in higher risk subgroups.[79] Every clinician has their own threshold and rules for when patients should either start antibiotics or contact the office. Additionally, clinicians change these recommendations based on the patient's history and symptoms. Since this is a pragmatic trial, we will not provide instructions to the clinicians. However, we will make it clear to the patients during informed consent that they should be comfortable with the plan and follow-up if they believe it is indicated.

Phase 2 refers to the period of randomized treatment allocation and follow-up; 14 days in total. Interested patients diagnosed with ARS who meet the inclusion criteria per IDSA guidelines and their clinician recommendation will be randomized.

There are three ways that patients could progress to Phase 2.
1 - They could directly enter into Phase 2 if they have had ≥10 days of ARS symptoms at their initial enrollment visit.

Via Double-Sickening:
2 - At enrollment: The participant may meet the conditions for double sickening at the initial enrollment visit and be directly entered into Phase 2. To qualify for double sickening per the IDSA guideline, the patient must have symptoms for 5 or more days, improve, and then have an onset of new or worsening symptoms. If a patient has had symptoms for 9 days or less, additional follow up questions will be added to REDCap to identify double-sickening. The questions will be, "Were you getting better then started to feel worse?" If Yes, the patient will be asked "How many days was your initial sickness?" If the patient

was ill for 5 or more days they will be asked, “Have you had worsening of sinus symptoms after initial improvement.” If Yes, they will immediately enter Phase 2.

3 - At day E9: The final way to enter into Phase 2 via double sickening. A participant may be enrolled into the study in Phase 1, improve and leave the study on E9 and thus did not proceed to Phase 2. At this point their participation in the study is complete. However, we will monitor patients who feel better and follow up in a staggered approach on Day 2, Day 4 and Day 7 after completion with a text message “Do you still feel better today? (Y/N)”. If they answer Yes, they will receive a text message saying “Thank you, we will check in again in 2 days time,” and send similar messages on Days 4 and 7. If on Day 7 they answer Yes, they will receive a text message saying “Thank you, we will not be checking in again but please feel free to reach out to us, we would love to get any feedback.” If they should become sick again and answer No on any of the three post-study questionnaires, this will be considered double sickening and they will be allowed to return to the study and enter Phase 2.

4.2.4 **Randomization**

Patients who enroll at ≥10 days of symptoms or qualify via double sickening will start Phase 2 and be randomized. Patients who start in Phase 1 (days E1-E8) and have not improved by day E9 will transition to Phase 2 and start interventions on day 10. Patients who improve by E9, but become sick again within 7 days and qualify via double sickening, may reenter the study and randomize into Phase 2.

Patients are randomized to receive one of four different treatment groups (see **Table 2**).

Table 2. Treatment Groups

Group 1: Antibiotics	Group 2: Placebo antibiotics + Intranasal corticosteroids
Group 3: Antibiotics + Intranasal corticosteroids	Group 4: Placebo antibiotics

4.2.5 **CRP testing**

Patients will have a fingerstick blood sample collected for CRP testing at enrollment. The rapid test will be performed by the research personnel using a QuikRead go Instrument (Aidian, Espoo, Finland).

5 STUDY ENROLLMENT AND WITHDRAWAL

Our goal is to study those who are more likely to have acute bacterial rhinosinusitis. The eligibility criteria exclude patients with known conditions, complications, comorbidities, or impairments to immune response which would preclude them from participating in a randomized trial with a possible placebo arm. In our pilot study, approximately 38% of people were excluded due to age (over 65) and thus with permission from PCORI we will increase the age of enrollment to 75. We believe the true exclusion rate by age was higher because most of the clinicians knew of the exclusion criteria and these older patients were not approached or pre-screened. Additionally, the NIH now states in their Inclusion Across the Lifespan, "Any age-related exclusions must include a rationale and justification based on a scientific or ethical basis."[78] Guidance from the FDA and ICH-E7 also acknowledge that efforts must be made to improve the inclusion of patients over age.[79]

Please see Section 7 for procedures on the inclusion of patients with symptoms of double sickening and proceeding to Phase 2.

5.1 Inclusion/Exclusion Criteria

**Table 3. Inclusion and Exclusion Criteria**[1]
<u>Inclusion</u>: *eligible* to participate if they are:<br>1. 18-75 years old; **AND** are experiencing either #2 or #3:<br>2. "persistent" symptoms or signs compatible with ARS or sinus infection lasting for 1-21 days without any evidence of clinical improvement;<br>Symptoms include:<br>○ facial pain or pressure<br>○ facial congestion or fullness<br>○ nasal obstruction<br>○ nasal discharge<br>○ no or reduced sense of smell<br>○ fever ≤39°C or 102°F<br>○ headache<br>○ bad smelling breath<br>○ fatigue<br>○ ear pain or pressure<br>○ dental pain; <u>**OR**</u><br>3. Onset with worsening symptoms or signs characterized by the new onset of fever, headache, or increase in nasal discharge following a typical viral upper respiratory infection (URI) that lasted 5–6 days and were initially improving (''double-sickening'').
<u>Exclusion</u>: *ineligible* to participate for any of the following:<br>1. allergy or intolerance to penicillin<br>2. received systemic antibiotic therapy in the past 4 weeks<br>3. prior sinus surgery[2]<br>4. complications of rhinosinusitis (facial edema (swelling), cellulitis), or orbital, meningeal or cerebral signs)<br>5. health care clinician determined IV (intravenous) antibiotics or hospital admission are required<br>6. pregnancy or breastfeeding<br>7. presence of a comorbidity or medication that may impair a patient's immune response as determined by a health care clinician<br>8. hospitalization in past 5 days<br>9. unable or unwilling to provide informed consent or comply with study protocol requirements<br>10. fever ≥39°C or 102°F **today**<br>11. taking intranasal corticosteroids (INCS) regularly in the past two weeks[3]<br>12. previously enrolled or participated in the feasibility phase or this stage of study

[1] If the patient had symptoms for <9 days at enrollment, the patient enters Phase 1 on day E(1 through

8), corresponding to the number of days they had the above ARS/sinus infection symptoms. If at day E9, the patient decides they have not improved and wish to proceed, they will enter Phase 2 and will be randomized. Patients with symptoms for ≥9 days at enrollment will directly enter into Phase 2 and are randomized at enrollment. Lack of improvement (see MOP, *How do I know if I should proceed from Phase 1 to Phase 2*). Patients will make this decision based on their symptoms and what they have discussed with their clinician. Research personnel should not make this decision. Following the nine-day waiting period with supportive care is the inclusion criterion prior to Phase 2, these patients are more likely to have bacterial sinusitis.
[2] Cosmetic surgery, such as rhinoplasty, septal deviation, etc. are not exclusionary.
[3] And are unwilling to stop its use while in the study

5.2 Intervention Assignment Procedures

5.2.1 Randomization procedures

We will follow guidelines for randomized controlled trials as per the ICH E6 Good Clinical Practice and ICH E9 Statistical Principles for Clinical Trials, in order to ensure proper randomization, blinding, allocation and implementation of these research procedures. We will use permuted block randomization by site with random block sizes (4, 8 or 12) to enroll patients into this study. The Research Electronic Data Capture (REDCap) web-based data entry system will integrate the randomization scheme and automatically assign enrolled patients to the corresponding allocation. All participating research personnel will be naive to antibiotic treatment assignments. However, personnel will not discuss data or which group patients are randomized to during the study or be able to predict treatment arms, assuring proper allocation concealment.

5.2.2 Masking procedures

To enhance the integrity of the study, we will utilize a masking procedure using different unique numbers. This will prevent research personnel from adjusting the randomization process or deducing what group patients are assigned to. The DCC will create this list of masking numbers which will correspond to the randomization scheme described above. The DCC will coordinate with the pharmacy to provide them with the list of masking numbers and their correspondingly coded group assignments. For purposes of emergency unblinding, non-research personnel within the DCC will have the key denoting which treatment group each masking number represents. We will use a distinct set of numbers to mask patient groups at each site. Furthermore, each site will be assigned an additional distinguishing numerical marker. If applicable, additional indicators will be included to distinguish sites with additional storage areas due to covering a large geographical area. The appearance and packaging of the antibiotics and placebo will match, obscuring the identity of the drug for patients and the research team. All of these measures will lead to successful true allocation concealment and proper blinding.

5.2.3 Reasons for withdrawal

A patient may opt to discontinue their participation in the study at any time or be withdrawn by research personnel.

Individuals may be withdrawn from the study if:

- A temporally associated SAE that is related to study participation, or for which the relationship with study participation cannot be ruled out.
- The PI and/or patient (in consultation with the patient's health care clinician, if necessary) determines the patient should not continue with study participation.

5.2.4 Handling of withdrawals

Withdrawals by the patient or by study personnel will be tracked via the Study Completion/ Discontinuation Form.

5.2.5 Termination of study

Termination of the study should occur at the end of patient accrual and completion of study-related procedures. The study may be terminated early at any time by the IRB, or other regulatory government agencies as part of their duties to ensure the protection of research patients. The Data Safety Monitoring Board (DSMB) may also recommend study termination due to efficacy or safety issues. See Section 9.0.

5.3 Recruitment and Retention Strategy

5.3.1 Recruitment

The identification, selection, recruitment, and retention of patients must be conducted in a systematic manner and should be representative. In order to address these points, we will be implementing in-clinic recruitment in six PBRNs/sites located in different geographic regions in the U.S. PBRNs have been described as "new clinical laboratories for primary care research and dissemination."[80] PBRN research has a long track record of widely disseminating findings and providing the impetus to changes in practice.[80–82] As the Agency for Healthcare Research and Quality states, "PBRNs provide a natural laboratory for a wide variety of physician and patient studies and are a rich source of medical data. PBRNs may be the best setting for studying the process of care and the manner in which diseases are diagnosed, treatments initiated, and chronic conditions managed in a "real world" setting. Furthermore, PBRNs provide a resource where effectiveness can be measured and an interface between patients and their community primary care physicians can be explored."[83] The Wisconsin site and its related university health clinics will function like a PBRN.

The six sites were chosen based on the expertise of the site investigators, history of success in trials, and geographic diversity. All six sites participated in the feasibility phase and demonstrated their readiness, and successful recruitment and implementation of the study protocol.
Dr. Barrett will be the site lead for University of Wisconsin-Madison, along with a team of researchers who have extensive experience in respiratory disease research, and have successfully worked together on numerous randomized controlled trials and observational studies, all with excellent recruitment and retention rates.

For in-clinic enrollment, study sites will engage with physicians, nurse practitioners and physician assistants in primary care and urgent care offices who will initially identify and evaluate potential

patients. Based on data from the feasibility phase, each director or coordinator determined which clinics and clinicians have the interest, capacity, patient population, and resources to help with recruitment for the full-scale study.

We anticipate each site/PBRN to recruit 18-20 patients per month for enrollment, of which 10-12 will result in randomization; this is consistent with the 60% of patients who progressed to randomization during the feasibility phase. We did not witness any hesitation from clinicians or patients into enrolling into a placebo controlled trial.

In order to change how ARS is treated, it is important to recruit patients from the places they seek care for ARS, specifically primary and urgent care offices, and via telehealth services. To reflect the growing number of patients with ARS that are seen outside of the clinic, we will implement a community-based approach to recruit these populations (see details in Section 5.3.2). Interested patients will be contacted by site research personnel who will discuss the study and screen the patient for eligibility. Similar to the feasibility phase, research personnel will complete the enrollment visit (informed consent, clinical information, demographics, distribution of SNI supplies, etc.) either in the clinical office, the patient's residence, or academic office. If the patient was initially seen via a telehealth visit, we will first screen these patients via telephone or Zoom, and if they agree to participate, research personnel will arrange an in-person meeting to collect CRP and dispense study and SNI materials. There will be no differences in any information collected, intervention timing, inclusion/exclusion criteria, or any aspects of the study, between clinic and telehealth patients.

**Table 4.**	**ACORN**	**CAPRICORN**	**PCRN**	**PSARN**	**WPRN**	**UW Health Clinics**
	VCU	GU	UCLA	PSCOM	Washington	Wisconsin
**a. Patients diagnosed with ARS over a 12 month period (network)**						
**Age**						
Adult (18-65)	10,515	10,522	11,900	4,065	5,489	13,840
**Gender**						
Female	7,345	7,419	6,112	2,784	3,652	9,116
Male	3,170	3,095	2,993	1,281	1,831	4,724
**Race**						
White	2,323	4,670	4,974	3,047	3,977	12,006
Black/African American	519	2,045	561	312	278	899
Asian	130	163	900	143	639	548
Other	7,542	3,644		563		387
**b. Number of Clinics**						
Total	526	50	64	15	120	80
Pilot Study	5-6	6-10	5	3	2-3	3-4
Full-Scale Study	20-40	20-30	≥10	12	15-20	≥10

ARS is widely prevalent, but enrolling patients into primary care clinical trials is time and resource intensive. As our research has highlighted, the majority of clinical trials are conducted in sub-specialty

practices, including interventions used in primary care settings.[88] The PBRNs/study sites have expressed their full support and interest to participate in this potentially transformative study. We anticipate each PBRN/site recruiting about 207 patients per year during the full-scale study. Each site will determine how best to use their personnel budget, but all budgets have enough resources for success at each site for recruitment. We recommend that patients have access to research personnel cell phones to enable close contact with patients and clinic sites. This is consistent with our prior studies.[89] Recruiting and enrollment for respiratory studies post-pandemic has been challenging, often requiring extra time and resources due to personal protective equipment requirements, clinic flows, and general patient apprehension. Recruitment estimates were based on what we learned from the feasibility phase and the estimated number of patients likely to meet inclusion and exclusion criteria within each the PBRN/site. Table 4a shows the candidate pool across all clinical sites with diagnoses of acute sinusitis (ICD-10 codes J01.0, J01.1, J01.2, J01.3, J01.4, J01.8, J01.9), extracted from electronic health record reviews of patients between 18-65 years of age who were diagnosed with acute sinusitis over a 12 month period (March 2024). The number of member clinics (primary care offices e.g., family medicine, internal medicine, urgent care) that will participate in the full-scale study phase has also been updated (Table 4b). Each PBRN/site recalculated their estimates and changed clinics they anticipate using based on feedback from clinicians, stakeholders and data provided by the DCC about enrollment during the feasibility phase.

5.3.2 **Outside of Clinic Enrollment**

The location and manner in which patients seek care for health issues, such as ARS, have shifted in the past few years as a result of the: (a) lack of/limited access to primary care, (b) increased access to virtual health care, and (c) emergence of telehealth-only services. To address concerns that we are missing outside of clinic visits, which have become increasingly common among patients with ARS, we will expand our recruitment methods. This community-based approach allows potential participants to learn about the study through advertisements and initiate contact directly with the research team by phone, email, or a brief REDCap questionnaire. After the initial enrollment visit and procedures, participants enrolled outside of the clinic follow the same study procedures as those enrolled in-clinic.

1) Initial contact:
Individuals may learn about the study through advertisements placed in community locations (e.g., pharmacies, health departments, community centers, libraries, university listervs, etc.), in clinics not currently partnered with, or through referrals by current study participants or others engaged by the research team. Interested patients will contact the study team via phone, email, or will complete a brief REDCap questionnaire indicating their interest. Research personnel will then contact patients to conduct a prescreening.

2) Clinical review:
If the individual is interested in the study and meets the prescreen eligibility, a study clinician will conduct a medical history review via a secure telehealth visit to confirm eligibility, ensure they are an appropriate candidate for this placebo-controlled trial, and to answer the gestalt question. This procedure is similar to how a visit is conducted in an urgent care setting. The clinician performing the visit will hold a medical license in the state where the patient is located.

3) Consent and enrollment:
If the study clinician determines that the patient is medically eligible, research personnel will contact the individual to review study details, obtain informed consent, review full eligibility criteria, and complete

the baseline questionnaires. Randomization rules will not change and will follow the same process for remote consent as currently approved.
As part of the enrollment process, we will continue to obtain CRP testing and ensure study medication delivery. The site team will determine whether the patient resides within a reasonable distance to allow for sample collection, SNI, and medication delivery before enrollment proceeds.

4) Ongoing participation:
The participant will otherwise engage in the study as per the usual protocol.

5.3.3 Retention

The goals and milestones proposed for this project are achievable by building upon the past successes and expertise of the PBRNs/sites and investigators. For the full-scale study, we have allotted 36 months to patient recruitment. Of the 87 patients who were randomized in the pilot study, 84 patients completed the study, 1 declined/withdrew and 2 were lost-to-follow-up (exceeding the goal of ≥68 retained). Overall diary completion rates by patients enrolled and randomized were also high, with 93% of daily diaries submitted (1,606/1,727). Sites will use similar approaches for communication, data collection, and engagement with patients; should significant issues arise in the full-scale study phase, the teams will seek input from the relevant committee(s) to help address these challenges.

5.4 Payment for Participation

Patients will receive payment of up to $160 total. Payments will be prorated for partial participation, early discontinuation and/or missed visits, according to the following schedule:

- $20 upon enrollment
- $20; upon randomization (day R) for patients starting Phase 2, or day E9 follow-up for patients starting Phase 1
- $40 at day R3 follow-up
- $40 at day R7 follow-up
- $40 at day R14 follow-up

6 STUDY INTERVENTIONS

6.1 Study Products

The interventions to be evaluated are:

- Amoxicillin-clavulanate 875 mg/125 mg taken orally, twice daily for 7 days
- Matching placebo amoxicillin-clavulanate taken orally, twice daily for 7 days
- Budesonide 32 mcg nasal spray, two sprays per nostril once daily
- Saline nasal irrigation
- Supportive care

6.1.1 Acquisition and preparation

Amoxicillin-clavulanate and budesonide will be procured and packaged by a central pharmacy (Doyle's Pharmacy, Houston, TX) and distributed to sites. A matching placebo will be prepared using similar excipient ingredients as in the amoxicillin-clavulanate tablets.

The SNI supplies will be provided from SinuCleanse® (Ascent Consumer Products). The study team will provide patients with well water (or are unsure) with a two-gallon supply of distilled water. Over-the-counter supportive care will be self-purchased by the patient, as is standard of care.

6.1.2 Packaging and labeling

The study products are currently approved for marketing by the U.S. Food and Drug Administration. Amoxicillin-clavulanate (AUGMENTIN®) is available in 875mg/125mg tablets.

Budesonide is a corticosteroid that is administered by nasal spray and is available over-the-counter. One bottle contains 120 metered sprays delivering 32 mcg per spray.

Products will be packaged and labeled with batch numbers, as per the masking scheme. The label will include instructions regarding dose and administration.

Instructions on how to use the saline irrigation device (Soft Tip Micro-Filtered Nasal Wash System), saline packets and distilled water will be provided to the patients.

6.2 Product Storage, Administration, and Accountability Procedures

The central pharmacy, Doyle's Pharmacy, will ship the packaged and labeled drugs, where they will be securely stored at each site's research pharmacy, research office, or at the recruiting site's clinics. Study personnel or a designee will collect the medications from the local site pharmacy, office, or clinic and distribute them to the patient. Each site will maintain a Product Dispensation and Accountability Log as part of the site's regulatory binder and record all products received and distributed to the patients.

6.3 Assessment of Compliance with Study Products

We will assess adherence to the products as reported in the daily diary and follow-up interviews. Given the high rates of data completion seen in the pilot, we will continue to provide access to daily surveys through email or via text message as per their preference. If a patient opts for paper, they are asked to mail the diaries after E9, R7, and R14. We will text patient reminders and ask for a response once interventions are completed on a daily basis. Patients are instructed to save their leftover, unused study medicine or empty medicine bottles for review. Patients are asked to count how many capsules are still left, if any and, if possible, take and send a photo for the file.

6.4 Supportive Care and Concomitants

Patients will be provided with following instructions:

“Thanks for participating in the NOSES study. In addition to any medications that your clinician provided, the following are commonly used to help relieve some of the symptoms you have. You may want to discuss with your clinician what they recommend.

1. For pain and/or fever:
   Take Acetaminophen (Tylenol) 500–1,000 mg every 6 hours as needed.
   - Do not take more than 3,000 mg in 24 hours.
   - Example: If using 500 mg tablets, you can take 1–2 tablets every 6 hours, but no more than 6 tablets in 24 hours.
2. For congestion or mucus:
   Take Guaifenesin (Mucinex) 600–1,200 mg every 12 hours as needed.
   - Do not take more than 2,400 mg in 24 hours.
   - Example: If using 1,200 mg extended-release tablets, take 1 tablet every 12 hours.
   - Tip: Drink plenty of water to help loosen mucus and improve the medication's effectiveness.
3. For cough:
   Take Dextromethorphan hydrobromide (Robitussin or Delsym) as directed:
   - 10–20 mg every 4 hours OR 30 mg every 6–8 hours as needed.
   - If using a liquid (e.g., 10 mg/5 mL), take 5–10 mL every 4–6 hours as needed.
   - Do not take more than 120 mg in 24 hours.
4. For nasal congestion:
   Take Pseudoephedrine (Sudafed) 120 mg every 12 hours as needed.
   - Do not take more than 240 mg in 24 hours.
   - May cause increased heart rate, jitteriness, or difficulty sleeping. Avoid taking it close to bedtime.
5. For nasal congestion (short-term use):
   - Use Oxymetazoline nasal spray (Afrin) as directed:
     - Spray 2–3 sprays into each nostril every 12 hours as needed.
     - Do not use for more than 3 consecutive days to avoid rebound congestion (worsening congestion after stopping the medication).
     - How to use Afrin:
       1. Clear your nasal passages before use.
       2. Tilt your head slightly forward while spraying into each nostril.

3. Avoid blowing your nose immediately after use to allow the medication to work effectively.

Patients will be asked to refrain from all non-prescription intranasal products outside of SNI (e.g. spray decongestants) and other non-prescription medications not on the provided list. As per the exclusion criteria, concurrent use of intranasal steroids is not allowed.

6.5 Saline Nasal Irrigation

During the enrollment visit, SNI use will be discussed with all patients. Instructions on how to use the SNI system, saline packets and distilled water will be provided. Additionally, instructions will list the use parameters for concentration (2% vs. 0.9%), frequency (1-3 times daily), and timing of irrigation, and use/cleaning of the filter-tipped SNI bottle that patients may adjust to accommodate their personal preferences. The SNI parameters are described in Section 4.2.2.

7 STUDY SCHEDULE

Patients with symptoms of ARS with interest in the study will complete a short pre-screening. We will assess patients by asking symptom-related questions to determine their eligibility for participation in the study. If the patient does not pass the pre-screening, and are willing, additional, demographic-related questions will be asked. Eligible patients will have an enrollment visit with a member of the research team to complete the informed consent process, collect baseline data, sample collections, and to receive SNI materials and instructions on supportive care. **Table 5** shows the schedule of events and data collection time points.

Based on the number of symptom-days, the enrolled patient will start Phase 1 or Phase 2. If the patient had ARS symptoms for less than 10 days, they will enter Phase 1, receive instructions on supportive care and complete a daily diary. At day E9, research staff will follow-up with the patient to determine if their symptoms have resolved and should be withdrawn from the study. Patients with ongoing symptoms will continue to Phase 2. If the patient had ARS symptoms for 10 or more days at the time of their enrollment visit, they will bypass Phase 1 and enter directly into Phase 2. On day R1 of Phase 2, the patient is randomized and will follow the same schedule for days R1-R14 (**Table 5**).

**Table 5. SCHEDULE OF EVENTS**	PHASE 1			PHASE 2					
STUDY DAY	E#	E# - E8	E9/R	R1	R3	R5	R7	R10	R14
Pre-screening	X								
Enrollment & Baseline	X								
C-reactive protein test (CRP)	X								
♦Randomization for <10 days - Phase 1	WAIT		X						
✤Randomization for ≥10 days - Phase 2	——➢		X						
Daily Symptom Diary	|→	→	→	→	⇴	→	→	→	→|
SNI and/or supportive care	|→	→	→	→	→	→	→	→	→|
Follow-up			X		X	X	X	X	X
Interventions				|→	→	→	→|		
Adverse event reporting	|→	→	→	→	→	→	→	→	→|
*Participation stipend	X		X		X		X		X

E# Day number corresponds to day of symptoms (e.g. symptoms started yesterday→E2)
E9/R Day of randomization
⇴ Primary outcome: mSNOT-16 score
♦Phase 1 (E#-E9; pre-randomization): patients with less than 10 days of symptoms
✤Phase 2 (R1-R14; post-randomization): patients with ≥ 10 days of symptoms or double sickening
*Participation Stipend: $20 at enrollment, $20 on day E9/R, $40 days R(3, 7, 14); up to $160 total
X: complete/collect on this day; |→: start day until →|: final day

7.1 Phase Determination

Prospective patients will initially be alerted of the study by a provider or clinic staff. Interested individuals will be pre-screened by research staff prior to completing enrollment and informed consent. Each PBRN/site has developed their own recruitment plan and workflow, and reviewed it with Lead

Project Coordinator Ms. Keisha Herbin-Smith (see Section 8.2 Pre-screening).

Based on the number of symptom-days, the enrolled patient will start Phase 1 or Phase 2. If the patient had ARS symptoms for less than 10 days, they will enter Phase 1, receive instructions on supportive care and complete a daily diary. At day E9, research staff will follow-up with the patient to determine if their symptoms have resolved and should be withdrawn from the study. Patients with ongoing symptoms will continue to Phase 2. If the patient had ARS symptoms for 10 or more days at the time of their enrollment visit, they will bypass Phase 1 and enter directly into Phase 2. On day R1 of Phase 2, the patient is randomized and will follow the same schedule for days R1-R14 (**Table 5**).

7.1.1 Double sickening

Our goal is to study those who are more likely to have acute bacterial rhinosinusitis. The inclusion and exclusion criteria are based on IDSA guidelines that aim to identify patients with a greater likelihood of having a bacterial infection and respond to first line antibiotic treatment. The IDSA guidelines state,

> "Which Clinical Presentations Best Identify Patients With Acute Bacterial Versus Viral Rhinosinusitis? Recommendations. The following clinical presentations (any of 3) are recommended for identifying patients with acute bacterial versus viral rhinosinusitis:
>
> i. Onset with persistent symptoms or signs compatible with acute rhinosinusitis, lasting for >10 days without any evidence of clinical improvement (strong, low moderate); or
> ii. Onset with severe symptoms or signs of high fever (>39°C [102°F]) and purulent nasal discharge or facial pain lasting for at least 3–4 consecutive days at the beginning of illness
> (strong, low-moderate); or
> iii. Onset with worsening symptoms or signs characterized by the new onset of fever, headache, or increase in nasal discharge following a typical viral upper respiratory infection (URI) that lasted 5–6 days and were initially improving (''double sickening'') (strong, low-moderate)."
>
> For the pilot study, the only inclusion criterion to enter into Phase 2 was the presence of symptoms for >10 days. However, at the suggestions of multiple committees and with PCORI's approval we have added the second inclusion criteria of double sickening into the full-scale study.

8 STUDY PROCEDURES/EVALUATIONS

8.1 Clinical Evaluations

During the enrollment visit and subsequent follow-up visits and calls, clinical information will be collected on the patient's ARS symptoms. In the pilot, all patients were seen in a clinical setting and evaluated by a clinician.

8.2 Pre-screening

Pre-screening refers to the activities conducted before obtaining written informed consent to determine initial eligibility and interest in a study. Pre-screening may be performed remotely or in-person, and may not include any research procedures.

8.2.1 Pre-screening versus research procedures

Research procedures are performed after enrollment; the patient has been deemed eligible during pre-screening, agreed to participate in the study, and signed the informed consent form. Research procedures are activities or interactions that would not have otherwise been performed if not for the study, such as the collection of identifiable data and fingerstick for CRP.

8.2.2 Information collected

Information collected during pre-screening should be limited to specific inclusion/exclusion criteria and other questions of suitability (see Pre-Screening Form). Pre-screening activities may start after the screening process has been explained by research personnel and verbal consent is provided by the individual.

Individuals who do not pass pre-screening: In order to protect privacy and confidentiality, only non-identifiable information should be recorded for individuals who are not eligible or decline further participation. Research personnel will retain the Pre-Screening Form and, once it is clear that the individual will not enroll, destroy any contact and identifying information.

Individuals who pass pre-screening: Research personnel will retain information from the Pre-Screening Form, which will be added to the study file when the individual has been enrolled. For individuals who pass the pre-screening and are interested in study, research personnel must obtain informed consent and authorization from the individual; contact and identifiable information may then be collected and retained in the study file.

8.2.3 Consent for pre-screening activities

Prior to starting the pre-screening activities, research personnel will use a script to alert about the process, and the potential patient must verbally consent. The script should include statements that address the following points:

- Introduction and purpose of pre-screening
- Appropriate time/setting to ask and answer questions
- Estimated time it will take
- Freedom to withdrawal from pre-screening at any time
- Individuals may choose not to respond to questions
- Privacy and confidentiality
- Information that will be collected, recorded, retained, or destroyed
- Allow individual to ask questions
- Verbal consent from the individual to proceed with pre-screening questions

8.2.4 Verbal consent script

Example script used to obtain verbal consent for the pre-screening process.

" Hello, my name is [NAME] from the [SITE]. Thank you for your interest in the NOSES study. Our goal is to understand how patients respond to different treatments for sinus infections. I would like to ask you a few screening questions about you, your symptoms, and health to see if you qualify for this research study. This should take about 5 minutes.

Is this a good time to talk?

I will go through a list of questions. You may choose not to answer these questions, or choose to stop this pre-screening at any time by telling me you want to stop. No matter what you decide, it will not affect your care. However, that may mean that you will not be eligible for this study.

Your privacy is important and we will make every effort to protect any information you provide to us. We ask your permission to record and keep only the information that cannot link or identify you.

After this pre-screening, if you are eligible and agree to participate in this research project, we would collect your contact information and begin the process of enrolling you in this study.

If you are not eligible, we will remove or destroy any identifying information collected during this conversation (e.g. phone number, email). With your permission, we would like to ask a few demographic questions which would help us learn more about our clinics -- this is optional and the information will not identify you or link you to the study.

Do you have any questions about this pre-screening?

Do I have your permission to ask these eligibility questions and document your answers? "

Continue to Pre-Screening Form.

8.3 Laboratory Evaluations

8.3.1 C-reactive protein

C-reactive protein testing will be performed via capillary blood sampling (fingerstick), using the QuikRead Go CRP (Aidian, Finland) instrument. Standardized instructions on how to collect and perform the test will be developed and included as part of the site training materials and visits.

9 ASSESSMENT OF SAFETY

9.1 Definition of Adverse Event

An AE refers to an untoward or unfavorable medical occurrence, including abnormal signs, symptom, or disease, temporally associated with the patient's involvement in the research, whether or not considered related to participation in the research. This includes the onset of a new event not present at baseline, or worsening during the course of the study even if present at the study start.

After discussion with PCORI staff and research team, we have decided to ask the following questions at all follow-up days, "Since we last spoke, have you had any trouble or side effects (such as, discomfort, symptoms) that you think are associated with the: study pill (antibiotic or placebo), nasal spray, or any other over-the-counter medicines you are taking?" Patients will also be able to contact research personnel at any time and report AEs.

9.2 Assessing Adverse Events

9.2.1 **Grading**

Adverse events will be graded using NCI Common Terminology Criteria for Adverse Events (CTCAE) Version 5.0. The CTCAE displays Grades 1 through 5 with unique clinical descriptions of severity for each event based on this general guideline:

- Grade 1 Mild; asymptomatic or mild symptoms; clinical or diagnostic observations only; intervention not indicated.
- Grade 2 Moderate; minimal, local or noninvasive intervention indicated; limiting age-appropriate instrumental activities of daily living (ADL).
- Grade 3 Severe or medically significant but not immediately life-threatening; hospitalization or prolongation of hospitalization indicated; disabling; limiting self-care ADL.
- Grade 4 Life-threatening consequences; urgent intervention indicated.
- Grade 5 Death related to AE.

9.2.2 **Relationship to study participation**

The relationship of the adverse events to study participation will be classified as:

- Not related
- Possibly related
- Definitely related

9.2.3 **Expectedness**

- Expected - An expected AE event is one that is known to be associated with the interventions or conditions under study. Expected AEs are identified in nature, severity or frequency in the study documentation (protocol, consent, package insert, etc.)

- Unexpected - An AE that is not known to be associated or consistent with the interventions or conditions under study. Unexpected AEs are not identified in nature, severity or frequency in the study documentation (protocol, consent, Investigator Brochure, package insert, etc.)

9.2.4 Serious adverse events

An event is considered serious if the AE:

- Results in death
- Is life-threatening or places the patient at immediate risk of death at the time of the AE
- Results in hospital admission or prolongation of existing hospitalization
- Causes or is likely to result in persistent or significant disability or incapacity
- Results in congenital anomalies or birth defects
- May, based on medical judgment, jeopardize the patient and may require medical intervention to prevent one of the outcomes listed.

9.3 Study Halting Rules/Stopping Criteria

Study procedures and/or interventions be suspended (temporarily or terminally) if:

- There is a patient death that the PI or IRB believes may be related to the study. The DSMB should give their opinion on such matters.
- A statistically or clinically significant number of hospitalizations occur that may related to the study.
- New information from outside studies reveal that it would be unethical to continue enrolling patients in the study.
- The IRB or PCORI decides that the study should be stopped.

9.4 Withdrawal of Individuals

Individuals may be withdrawn from the study or the intervention(s) may be discontinued if:

- A temporally associated SAE that is related to study participation, or for which the relationship with study participation cannot be ruled out.
- The PI and/or patient (in consultation with the patient's health care clinician, if necessary) determines the patient should not continue with study participation.

9.5 Reporting Procedures

9.5.1 Reportable events

As the single IRB reviewing this trial, Biomedical Research Alliance of New York (BRANY) has provided the following guidance and timelines on reportable events: https://www.brany.com/forms-and-downloads/.

**Table 6. Reporting Timelines**	
Unanticipated Problems Involving Risks to	Report per occurrence within 5 days

Patients or Others	
Serious Adverse Events*	Report per occurrence within 5 days
Complaints	Report per occurrence within 5 days
Major Deviations (see section 14.6)	Report per occurrence within 10 days
Minor Deviations (see section 14.6)	Report in aggregate with continuing review or study closure

*Expedited reporting: events that are serious, unanticipated, and related to the intervention, and deaths for which the relationship with study participation cannot be ruled out, should be reported to the DSMB Chair within 48 hours upon knowledge of the event. The DSMB Chair will determine if stopping criteria have been met and/or if the full board needs to convene for an ad-hoc review.

9.5.2 Other adverse events

Non-serious adverse events will be evaluated by the DSMB during scheduled meetings and reported to the sIRB during the annual continuing review.

9.5.3 Reporting of pregnancy

Individuals who are actively pregnant and breastfeeding are not eligible to enroll in this trial. Should the research team be informed of an individual becoming pregnant while enrolled in the study, the site PI will recommend withdrawing the individual from the interventions (amoxicillin-clavulanate, placebo, and budesonide spray).

SNI is considered safe during pregnancy and commonly used for pregnancy rhinitis and would not need to be discontinued. The interventions and recommended supportive care (over-the-counter medicines) used in this protocol are often used in standard care and should not add significant risk to pregnancy; however, in an abundance of caution, we recommend the individual speak with their clinician.

We generally ask individuals who withdraw from the intervention if they would agree to continue with the follow-ups, diaries and other data collection activities per protocol.

9.6 Safety Oversight

The PIs and Co-Investigators, along with the Committees and independent DSMB, will monitor the study for adverse events, adherence to protocol, and patient accrual and withdrawal.

9.6.1 DSMB

The DSMB is an independent panel of experts which will periodically review and advise on the safety and rights of human research patients enrolled in this multi-center clinical trial. The DSMB will assess and provide unbiased recommendations of the ongoing scientific validity of the study, exposure to undue risk, and adverse event reports. A DSMB Charter is attached in the **Appendix**.

This DSMB is composed of a group of seven (7) experts or patient representatives with experience in the conduct of clinical research, biostatistics, and/or treatment of acute rhinosinusitis. The DSMB is wholly independent of the research team and has no direct involvement in the conduct of the study. The board will be chaired by Dr. Dan Tancredi, a statistician with expertise in clinical trials and infectious disease.

DSMB meetings will be held remotely via Zoom. The DSMB met for an initiation meeting during the feasibility phase where the PI provided an overview of the protocol, and the board reviewed the charter and format and content of future reports. The DSMB will provided with the full-scale study protocol, analysis plan, informed consent form, and data collection forms.

9.6.2 DSMB plan for full-scale study

The DSMB will determine the schedule for safety data review. The current planned reviews of safety data will occur after 10%, 20%, 40% and 70% data completion. Meetings will include a review of AE and SAE, including their occurrence, grading, expectedness, and relationship to study participation.

Safety reports prepared by the DCC will be presented to the DSMB as blinded reports. The format and information to be included on the DSMB reports will be agreed on between the DSMB and DCC during the pilot. DSMB members may determine that unmasking may be required due to a safety concern or other emergency. A report may be unblinded at their request by an unmasked member and reviewed in a closed session meeting. Standard procedures for unmasking and unblinding will be added to the final DSMB plan.

In addition to safety data, the DSMB may provide recommendations during the study relating to the selection, recruitment and retention of patients, improving adherence to the protocol and interventions, and procedures for data management and quality control.

9.7 Safety and Blinding

We will thoroughly monitor for treatment failure, or new symptoms and treating clinicians will be allowed to add medications as clinically indicated. Additionally, if symptoms worsen after 72 hours, all groups will be advised to alert both local research personnel and their primary care provider for further investigation and treatment, as clinically indicated. While we will not change any group assignments, treating clinicians may stop the interventions or make changes they believe are clinically indicated.

9.7.1 Breaking blind

As deemed clinically necessary, treating clinicians may request to learn which intervention the patient received during the study through 24-hour access to a Blinding Specialist. This will be correlated with patient reported AEs. A designated research personnel, likely the site PI, will have to determine if there is an indication to break the blind. Indications will be most SAEs if a patient is being treated emergently, etc.

Pre-assigned personnel in the DCC or clinical sites, (not on or affiliated with the research team), will have access to the patient Study ID numbers, medication assignment number and the corresponding code break, which they can then provide to the treating clinician. They can be reached at the following phone number: (+1) (540)-824-5734. We have used this technique with past studies to help preserve proper blinding of the research team throughout the study. All changes to an intervention, such as withdrawal or discontinuation (temporary or permanent), or addition of new treatment or medications, will be documented in the patient study file. All primary analyses will be by the intention to treat principle.

9.7.2 Out of range CRP value

The point of care CRP test is for research purposes only and will not be used to diagnose or guide treatment. CRP results are NOT to be shared with the patient, clinician or research staff during the study. However, if a CRP test value is >50, the CRP results should be shared with the patient by the site PI after the patient is out of the study. If the patient would prefer the site PI also shares with a clinician the site PI will do so at the direction of the patient.

10 STATISTICAL CONSIDERATIONS

10.1 Sample Size Considerations For Full-Scale Study

This study is designed as a parallel four-group randomized clinical trial for comparative effectiveness. The primary outcome measure is the mSNOT-16 score and the groups will be compared for effectiveness by computing the mean change score from baseline to Day 3. The mSNOT-16 is a validated, easy to use instrument; it takes less than 5 minutes to complete, and has a Cronbach's alpha ranging from 0.82 to 0.91. It is the gold standard as a sinus symptom-specific quality of life measure. To inform our sample size calculations and primary outcome assessment, we relied on the Garbutt et al. validation study, which reported a mean baseline SNOT-16 score of 1.71 with a standard deviation of sd=0.54 and a change score at Day 3 of 0.58 (sd=0.52) units of improvement. Thee minimal important difference (MID) for patients with ARS seen in primary care was also estimated to be 0.5 units, which corresponds to a small change when compared to patient-reported global scale.[71]

The sample size calculations for this application were conservatively done to estimate the mean change in each group with high precision (95% confidence interval with a margin of error of +/- 0.05) rather than to detect significant differences between the groups. Based on the sd=0.54 from the Garbutt et al. study, we previously proposed to randomize 2,232 patients to 4 groups with 1:1:1:1 ratio (1.2*1,860 with 20% dropout rate) and to consent 3,720 patients assuming that 40% of the enrolled patients would improve on their own and not need to be randomized.

We reassessed the sample size based on the data we obtained from the pilot phase. Using the observed changes on mSNOT-16 for 4 groups (0.30, 0.40, 0.42, 0.29) with the maximum sd=0.60, we computed the sample size again to power for a 4*3 factorial design to ensure sufficient power (80%) for subgroup effects with a subgroup variable up to 3 levels (for example: the clinician impression/gestalt variable). The means for the 3 levels were assumed to be 0.30, 0.40 and 0.50 with sd=0.60. We confirmed that the originally proposed sample size of 1,860 provides 80% power at alpha(α)=0.005 (allowing 10 pairwise comparisons of interest with Bonferroni adjustment) to detect at least one significantly different pair among 4 treatment groups (effect size=0.10), 99% power to detect at least one significant pair in the subgroup variable (effect size=0.14) and 100% power to detect at least one significant interaction (effect size=0.17) with 155 per cell for 12 treatment combinations. Therefore, our sample size will allow us to detect small effects when we evaluate heterogeneity of treatment effects, one subgroup at a time.

While the proposed sample size will provide sufficient power to compare the effect of four interventions across pre-defined subgroups of up to 3 levels, 465 patients per group will also provide 100% power to do an equivalence test for each one of the six possible comparisons across 4 groups with a Bonferroni-adjusted alpha (α)=0.05/6=0.008 and a margin of equivalence of 0.25, which was selected as half of the MID=0.50 (per Garbutt et al.) following published guidelines about the selection of such margins. If the difference in the change between any two groups is statistically not significant (p>0.008), we will use the two one-sided test (TOST) procedure to conduct equivalence testing with a confidence interval [(1–2α) × 100% (~99%)] where α=0.008 and we will conclude equivalence if the 99% confidence interval for the difference between groups falls within the [-.025, 0.25] interval. If equivalence cannot be shown or the

difference is significantly different but not clinically meaningful, we will use the same margin to assess non-inferiority or superiority comparing the lower or upper bound of the 95% confidence interval to -0.25 or 0.25 respectively.

10.2 Final Analysis Plan

Basic Statistical Analyses: Descriptive statistics for the overall enrolled sample (Phase 1) and for the overall randomized sample (Phase 2) by treatment groups will be presented using means, standard deviations, median and range for continuous scores and frequencies and percentages for categorical variables. Due to the 4-group design, bivariate statistics for continuous scores will be conducted using Analysis of Variance (ANOVA) with Bonferroni adjusted pairwise comparisons (p-val=0.05/6=0.008). Categorical variables will be tested using Chi-Square or Fisher's exact test. Within group changes will be tested using paired t-tests for means and Symmetry test for paired proportions. All the unadjusted/bivariate comparisons will be based on patients with available data at each timepoint. Primary outcome assessment will be done according to both intent-to-treat (ITT) principle and per-protocol.44 Trends in the mSNOT-16 scores will be illustrated with longitudinal data for both phases. Analyses will be conducted in Stata 18 or R.

Aim 1: Primary Outcome Assessment: Symptom responses will be assessed using the patient validated modified Sino-Nasal Outcome Test (mSNOT-16), a disease-specific quality of life instrument validated for use in primary care.65 For this study, clinical improvement is defined as a 0.5-point decrease in the patient-reported mSNOT-16 scores from day 1 to day 3. The primary outcome (change in mSNOT-16 from day 1 to day 3) will be used to assess comparative effectiveness of the interventions tested with four groups

Group 1: Antibiotics (AB)	Group 2: Placebo antibiotics + INCS
Group 3: Antibiotics + INCS	Group 4: Placebo antibiotics

While there are six possible pairwise comparisons, we will focus primarily on the following:

1. To test if antibiotics are effective, test group 1 (AB only) vs group 4 (placebo AB)
2. To test if INCS are effective, test group 2 (placebo AB + INCS) vs group 4 (placebo AB)
3. To test if INCS makes any difference with AB, test group 1 (AB only) vs group 3 (AB + INCS)
4. To compare AB to INCS, test group 1 (AB only) vs group 2 (placebo AB +INCS)

We will use a Bonferroni adjusted Type I error (alpha)=0.05/6=0.008 for each pairwise comparison. Upon a significant F-test from Analysis of Variance (ANOVA) of the change in mSNOT-16 score by 4 groups indicating that there is at least one significant pair, we will move on to conduct pairwise comparisons. If there is a significant difference between any two groups, we will use the confidence interval approach to evaluate where the 99% confidence interval around the mean difference falls within the equivalence margin of [-0.025, 0.25] to assess if the statistically significant difference is also clinically meaningful (i.e., less than 0.25) and whether there is evidence to show non-inferiority or superiority of a treatment. For statistically-not different pairs or if the overall F test is not significant, we will conduct equivalence testing for each pair using a 99% confidence interval, which should be within

the equivalence margins. If equivalence is rejected, depending on the location of the confidence interval in relation to zero difference line and to the margin, we will examine if there is evidence for non-inferiority or superiority of a treatment group vs another or the results are inconclusive.

Longitudinal trends in the mSNOT-16 scores from Day 1 to Day 14 during Phase 2 will be analyzed using linear mixed models with random effects at study site and patient level. The models will include a treatment group indicator, a linear time indicator (1 through 14) and their interactions to examine the trends over time vary by treatment group. They will also be adjusted for potential baseline confounders such as age, gender, CRP, double-sickening indicator. Predicted scores will be computed for Day 3 and contrasts will be performed. Group differences will be evaluated with 99% confidence intervals to further test if the conclusions of the bivariate analyses described above hold when the differences are adjusted for potential confounders.

10.2.1 Secondary outcomes

Secondary outcome measures such as global assessment of change in symptoms ("a lot worse", "a lot better", "no symptoms", a little worse" and "a little better" and "the same") or changes in symptoms since participating the study (on a continuous scale from 1 to 10, 1 being much worse and 10 being much better) will be examined at Day 3 first to compare treatment groups. The trends in these outcome measures will also be examined using longitudinal analysis models appropriate for the measurement scale of the outcome (categorical vs continuous). The associations between the secondary outcomes and the primary outcome measure mSNOT-16 will also be examined for available follow-up days for the overall sample as well as by treatment group. Another secondary outcome measured at multiple follow-up points, the Work Productivity and Impairment (WPAI) day 1 to day 3 questionnaire, will be analyzed using linear mixed models (four subscale scores ranging 0-100) that will include a treatment group indicator, a time indicator and their interactions with random effects at study site and patient level to examine whether there are differential trends in patients productivity that can be attributed to the treatments.

Aim 2: To identify which patient subgroups benefit most from oral antibiotics (amoxicillin-clavulanate) versus INCS.

The analyses in this aim will examine whether the differences between treatment groups are moderated/modified by certain characteristics of the patients at baseline. We determined that the following list of variables might be considered to examine heterogeneity of treatment effects: an elevated CRP level, double-sickening,) evidence of purulence on clinical examination, change of smell, pain in the teeth, provider clinical impression/gestalt, and type of visit (in-person vs telehealth). These clinical factors are likely to indicate bacterial infection and hence, treatment groups with antibiotics may be more effective. Our study is powered for a 4*3 factorial design and allows multiple comparisons with a subgroup variable up to three levels. Therefore, we will be able to compare treatments for each subgroup using a paired t-test for subgroups with a sample size of 155 within a treatment group. Heterogeneity of effects can also be tested with interaction terms in regression models. To avoid three-way interactions in linear mixed models (treatment*time*subgroup variable), which would require a much larger sample size than the proposed N, we will conduct linear regression analyses of the change

in mSNOT-16 in Day 3, adjusting for baseline mSNOT-16, and including a subgroup variable indicator, a treatment group indicator and their interaction. Other subgroup variables will be included in this model as main effects for control variables. The sample size will be a limiting factor for the inclusion of multiple interaction terms at once. Therefore, we will do subgroup analyses with one interaction at a time.

Aim 3: To identify which patient subgroups improve with supportive care and do not require antibiotics or INCS.

The goal of the analyses for this aim will be to better understand and predict why some patients will improve on their own during enrollment phase (Phase 1). The outcome measure will be an indicator of which patients will get better on their own and which patients will move to the next phase of the study to receive treatment. The associations between the outcome variable (binary) and demographic and clinical characteristics will be examined first using bivariate analyses such as two-sample t-tests and chi-square test. To determine the factors that may be causally related to the outcome, we will conduct logistic regression analyses including relevant demographic variables and subgroup variables listed in Aim 2 as potential predictors of bacterial infection that may decrease the likelihood of improvement during Phase 1. As an exploratory analysis to predict the outcome, we will also do a cluster analysis of the symptoms and include the clusters in a logistic regression model along with elevated CRP and double-sickening indicator.

10.2.2 Analysis of other outcomes

Other outcomes include illness duration measured using a symptom diary, treatment failure, re-consultation for the same symptoms, and adverse events which will be compared across four treatment groups using regression models or mixed models appropriate for the measurement scale.

10.2.3 Sensitivity analysis to determine the impact of key assumptions

The robustness of the findings from the linear mixed models could impact any conclusions, therefore we propose various sensitivity analyses to consider issues that could change findings. The sensitivity analysis to be performed include:

(i) Examining outliers, influential observations, and studentized residuals for outlier effects on results.

(ii) Performing per protocol analysis on the primary outcome and including adherence as a covariate in the mixed effects models.

(iii) Handling missing data using multiple imputation methods and assessing the degree to which results are affected by the missing data assumptions. There is vast literature on how to deal with missing data, but most of these methods assume that the data are missing at random (MAR). However, MAR may not be a valid assumption. Therefore, we will first explore if there are significant differences between patients with and without missing data by baseline characteristics and/or if any group has more missing data than any other to evaluate the nature of missingness. If we conclude that the missingness may not be at-random, then we will we will perform analyses using appropriate imputation methods and sensitivity analyses (to examine sensitivity of the results to various

assumptions about patterns of missingness) such as pattern-mixture models (PMM) or Full-Information Maximum Likelihood models.

Since this is a multi-center trial, further sensitivity analyses will include:

(iv) Comparing baseline results for each site separately, with and without provider as a random effect (patients within provider).

(v) We will perform sequential monitoring analyses and quality control methods for the mSNOT-16 scores “over time” to determine if the directions of changes are different (beneficial or harmful) between sites, treatment combination groups and groups within sites. The examination of patterns in mSNOT-16 scores over time is extremely valuable since the recruitment period is for 3 years.

11 SOURCE DOCUMENTATION

Data for this study are entered by the research staff directly into the electronic database, or self-reported by the patient in REDCap or on paper. The case report/data collection forms listed below will serve as source documentation. Based on the feasibility study we expect over 95% of patients will enter data directly. For those that enter on paper, research personnel will enter in the follow-ups via phone calls and the patients will do the daily diary via paper. The plan will be to collect the paper diaries within 7 days of their final diary.

Each site will maintain appropriate study records for this study. Hardcopy records with identifiable and/or protected health information will be secured at each site (e.g. locked filing cabinet or office) with access limited to the relevant research personnel, the DSMB, and parties with authorization. The Pre-Screening, Study Eligibility, Baseline Health, Demographics and General Health, Informed Consent forms will be completed by research staff and/or patient at the first visit, either in-person or remotely. Patients will complete their daily diaries in REDCap or on paper. If patients complete their entries on paper, the research coordinator will enter it into REDCap. At enrollment, patients have the option to complete follow-up forms in REDCap or over the phone with a member of the research.

Regarding data collection: Repeating forms should be collected via the same method each time; e.g. if a patient chooses to directly enter follow-up data in REDCap on Day R3, they should also enter the Days 5, 7, 10, and 14 follow-ups in REDCap. If for some reason this becomes not possible and the research staff has to collect a follow-up, or vice versa, a note explaining the change will be added to the record.

**Table 7. Data Collection Form**	Method	Timepoint	Completed by
Pre-screening	REDCap	Enrollment	Research staff
Study Eligibility	REDCap	Enrollment	Research staff
Baseline Health	REDCap	Enrollment	Research staff or patient self-report
Demographics and General Health	REDCap	Enrollment	Research staff or patient self-report
Research Clinician	REDCap	Enrollment	Research staff
Phase 1 to 2 Eligibility	REDCap	Day E9	Research staff
Randomization	REDCap	Enrollment or Day E9	Research staff
Daily Symptom Diary	REDCap survey link, paper	Days E#-E9, R1-R14	Patient self-report
Follow-up	REDCap (call or survey link)	Days R3, R5, R7, R10, R14	Research staff or patient self-report
Study Completion/Discontinuation	REDCap	Day R14	Research staff
Post-Study Questionnaire	REDCap (call or survey link)	2, 4, and 7 days post-E9	Research staff or patient self-report
Event-Driven			
Adverse Event	REDCap	As needed	Research staff*
Serious Adverse Event	REDCap	As needed	Research staff

*If an AE is reported during a follow-up call, research staff will complete the AE Form. If the patient completes their follow-up electronically and reports an AE, the research staff will be alerted by REDCap. The research staff will phone the patient to complete the AE Form.

11.1 Study Records

Patient study records will be maintained at each site by research staff. These will be managed separately from the study database to minimize sharing of personally identifiable Information beyond what is necessary to conduct the study. These records should be made available for monitoring during site visits.

- Informed Consent and HIPAA authorization
- Informed Consent Process Documentation
- Contact Information

12 STUDY STARTUP AND SITE MONITORING

12.1 Startup Plan

Below is an abbreviated version of the startup plan. Site operations will be detailed in the Manual of Procedures.

12.1.1 Required training for engaged research personnel

An individual is “engaged” in human subjects research when the individual, (i) intervenes or interacts with living individuals for research purposes; or (ii) obtains individually identifiable private information for research purposes [45 CFR 46.102(d),(f)].

All personnel who are engaged in research are expected to meet their institutional training requirements:

- Human Subject Protection, through the Collaborative Institutional Training Initiative or equivalent
- Health Insurance Portability and Accountability Act (HIPAA) and Human Subjects, through the Collaborative Institutional Training Initiative or equivalent
  Note: Human Subject Protection Training and HIPAA and Human Subjects Training are two separate courses. Both must be renewed every three years.

The following may be required per the individual’s institution:

- Good Clinical Practice (GCP) training for NIH funded investigators and staff involved in clinical trials
- Financial Conflict of Interest Training for investigators funded by a Public Health Service agency or cooperative group

Site-specific training: There may be specific training as per the site of employment. Staff members who are new to the study or institution must coordinate their onboarding and training procedures with the relevant offices at their respective institution (e.g. human resources, research oversight). For example, GU research staff using vehicles to conduct study activities in the community must have vehicle use and driver authorization from the GU Office of Risk Management.

Study-specific training: Prior to site initiation, the site Project Coordinators will conduct study-specific training with the research staff. This includes but is not limited to training on the protocol, recruitment activities, informed consent process, engaging with patients, CRF handling, general day-to-day study tasks, etc. The Data Manager will also conduct training specific to the data handling, REDCap data capture application, quality control, etc.

Stakeholder engagement training: Team members working with stakeholders will complete training through PCORI’s Building Effective Multi-Stakeholder Research Teams, Engaging Stakeholders (Inclusion: The Starting Point for Effective Teams, Best Practices in Engaging Stakeholders, Addressing Contextual Challenges to Engagement) and Working As A Team (Best Practices in Multi-Stakeholder Team Science, Leading and Contributing to Team Decisions, Solving Challenges and Barriers to Teamwork).

The Lead Research Program Manager, Ms. Tina Tan, will maintain documentation of completed training activities in the study regulatory file and coordinate with site personnel to maintain their respective site's regulatory file.

12.1.2 Manual of procedures and forms

Site operations are detailed separately in the Manual of Procedures (MOP).

12.1.3 Institutional review board (IRB)

Once study materials are finalized, IRB applications will be submitted to the single IRB, BRANY. Materials to be reviewed include the protocol, informed consent form, HIPAA waiver authorization, case report forms, patient diaries, advertising materials and website content. No study enrollment or procedures may commence until IRB approval is secured.

When IRB approval is secured, the Lead Research Program Manager will be responsible for maintaining the study regulatory documentation (approval letters, amendments, continuing reviews, protocol deviations, etc.) and coordinating with site personnel on their respective site's regulatory file.

12.1.4 Registration of trial

Trial NCT06076304 was registered at the NIH trials registry ClinicalTrials.gov prior to enrollment. The Lead Research Program Manager is responsible for registration and regular updates.

12.1.5 Site initiation visit

Site visits by the lead PC and DCC representative were conducted during the Feasibility Phase. Additional site visits may occur during the full-scale study on an as-needed basis.

12.2 Data Coordinating Center (DCC) Functions

12.2.1 Data quality assurance

The DCC is equipped with several systems and tools that ensure efficient, secure and timely data capture. For this study, the DCC will generate the randomization lists, program and format the different study questionnaires, develop the CRFs, set up the data collection methods, develop the study's central REDCap database, design the study's tracking dashboard, and execute all statistical analysis outlined in Aims 1-3. The central database will house data collected from patients either directly or manually by study staff. REDCap is a secure, HIPAA compliant, IRB-approved, web-based application designed for managing online surveys and for communication between study sites. Data quality checks are embedded into REDCap which will only allow for certain data or ranges of data, which provides a reduction in the number of queries to be resolved. Case report forms will be designed to prevent

missing data by requiring necessary items to be answered, or notifying the user that items are missing, before moving on.

12.2.2 Data collection approaches

Patient-reported surveys will be collected via three modalities: 1) directly into REDCap web-based forms through a survey link received via email, 2) directly into REDCap web-based forms through a survey link received via text, or 3) paper forms. Based on the feasibility phase, we anticipate nearly 98% of forms will be collected via REDCap. We worked closely with the PBRNs/sites and PAB to help reduce patient burden and direct data entry seemed to achieve this quite well. If patients do not want to enter data directly into REDCap, research personnel will collect information by phone during a priori set days. At the enrollment visit, site personnel will provide patients with the ability to complete questionnaires however it best suits them, providing them with paper questionnaires, or login information so that they may access REDCap directly. The goal of utilizing these varied modalities is to improve data collection while still ensuring the completeness, accuracy, and timeliness of the data. These different methods will be developed with quality checks and measures to only allow certain ranges and values to be entered. These methods will accommodate patients' preferences, reduce study burden, and improve compliance.

12.2.3 Web-based data collection

Site research staff will assist patients with their first login to REDCap. The Data Manager will train site personnel in the use of REDCap so they may provide support directly to their patients as needed. The Data Manager will distribute training materials covering all aspects of data collection to site personnel.

12.2.4 Tracking dashboard

The DCC will create a tracking dashboard in REDCap that will allow research personnel to assess in real time if patients have completed their questionnaires. The DCC will assist the research team in making sure the patients are completing their questionnaires according to the schedule by setting up automatic reminders and on-demand alerts. The automated alert system will send an email or text to research personnel alerting them to non compliance. This system will track and identify when to follow up with patients at different points throughout the study, including adverse event follow up, missing Daily Diary entries, or missing Follow Up completion. Notifications and alerts will be sent to site personnel responsible for follow up and for DCC personnel responsible for monitoring. The scheduled reminders can identify those patients who have outstanding data by a certain time each day, and remind them to complete their questionnaire.

12.2.5 Data reporting

The Data Manager will also generate monthly reports, assisting the site coordinators with monitoring patient enrollment and completion, missing data, attrition, as well as completion of critical assessments. The Data Manager will train the site staff at each site on the proper methods to record and report all reasons for dropout and missing data, monitoring all patients via reports. Frequent meetings with each of the study sites will be held to address any issues related to data collection in order to minimize risk of

missing, incomplete or otherwise poor quality data. A primary goal is to prevent patient dropout by facilitating regular, attentive, and respectful contact from study staff.

12.2.6 Handling of metadata

At the beginning of the project, the Data Manager and Analyst will establish a data dictionary. The Data Manager will provision access to this metadata as necessary in order to facilitate secure data access. This data will only be available behind the DCC firewall and to project team members.

12.2.7 Network data security

Several systems are employed to protect the DCC's network from unauthorized access and malicious program attacks: 1) firewall; 2) limited and secure remote access; 3) intrusion detection; 4) virus prevention and protection; 5) spam filtering; 6) website filtering; 7) server and network monitoring; and 8) incident reporting.

12.3 Clinical site monitoring

The data manager, regulatory coordinator, Lead Project Coordinator, or other authorized representative acting as such, may access clinical trial documentation in order to ensure the protection of the rights, safety and well-being of individuals participating in clinical research. The purposes of these reviews are to ensure compliance with the clinical trial protocol, GCP and the integrity of the data collected.

12.3.1 Monitoring

Each study site will be continuously monitored by the study team as per the protocol and manuals. As part of the quality control program, a monitor from the DCC will be available for this study. At least one monitoring visit will be scheduled per year per site. This visit will be in person or via zoom.

As part of regular remote monitoring a built in query system will be utilized in the electronic data capture. The monitor will open queries on any data quality or missingness in the database and send an automated email or text to the site personnel responsible. A log of queries and resolution will be provided during the monitoring visit and stored in the database.

Monitor responsibilities will include: (1) Providing information and guidance to study personnel on the management of study patients according to protocol procedures; (2) Ensuring that the protocol is being followed properly; (3) Assessing general organization, security and maintenance of regulatory documentation and study correspondence; (4) Review of all enrolled patients including verification of patient eligibility and informed consent; (5) Review of hard copy forms to ensure completion as well as consistency with study database entries (for virtual visits, hard copy forms will be uploaded by site personnel to a secure shared document storage site to be viewed by DCC); (6) Review of responses to queries generated by the DCC.

At each site visit, any incomplete documentation, errors or discrepancies will be noted and reported by the monitor to the study coordinator and site PI for review and investigation. When possible, items will be resolved with site personnel at the time of the visit. Any changes will be tracked electronically. At the end of each site visit, the monitor will prepare a report for the site study personnel summarizing the above items and the corrective measures to be taken. This report is also filed by the DCC. The added level of quality control offered by the monitor supplements computer-based checks and other quality control activities (described in the next section), and will increase data quality over an approach that relies largely on computer-based checks and audits.

12.3.2 **Data audits**

The DCC conducts periodic audits at the clinical sites. The DCC will schedule a site visit with each clinical site at least once per year, in person or via zoom, with an initial visit to be scheduled for all sites within the first eight weeks of data collection. The DCC randomly selects 5 percent of active patients at the site to conduct a complete audit to compare data in the study database to data on source documents. If this is a virtual visit, site personnel will upload source documents to a secure shared storage site to be viewed by the DCC. During the visit, the DCC verifies that patients have signed consent documents, checks any lab procedures and records, verifies measurement tool specifications, ensures proper security practices, and that clinic staff have up to date study documentation available. The DCC conducts an exit interview with the site PI or other representing personnel at the end of the site visit to review the visit and obtain initial feedback. The DCC provides a written report documenting all findings from the audit to each site PI and the DCC study file within two weeks of the audit. The site PI must send a response letter to the DCC within three weeks of receipt of the audit report to document the site's response to audit findings.

13 ETHICS/PROTECTION OF HUMAN SUBJECTS

13.1 Ethical Standard

The investigators will ensure that this study is conducted in full conformity with the principles set forth in The Belmont Report: Ethical Principles and Guidelines for the Protection of Human Subjects of Research of the US National Commission for the Protection of Human Subjects of Biomedical and Behavioral Research and codified in 45 CFR Part 46 and the ICH E6.

13.2 Institutional Review Board

All participating university Institutional Review Boards (IRB) will rely on a single IRB to conduct the regulatory review and oversight of this study. The single IRB must review and approve the study protocol before commencing enrollment. All patient-facing materials, and internal and external advertisements will require prior approval from the IRB (see 12.1.3).

13.3 Informed Consent Process

Informed consent is a process that is initiated prior to the individual's agreeing to participate in the study and continuing throughout the individual's study participation. This process will be conducted in a setting where privacy is ensured. Extensive discussion of risks and possible benefits of the interventions will be provided by trained research personnel. Informed consent forms describing in detail the study interventions, procedures, and risks are given to the subject and written documentation of informed consent is required prior to starting any intervention or procedure. The research coordinators will discuss all information outlined in the informed consent document with prospective patients to ensure that they understand the nature of the research and can voluntarily decide whether or not to participate. Prospective patients will be encouraged to ask any questions and given as much time as needed before deciding to participate. A printed or digital copy of the informed consent form must be provided to the patient and a copy must also be retained by the research team in the study records. The patient will be informed that they may discontinue study participation at any time.

13.4 Remote Informed Consent

The remote consent process applies for instances where the research staff and patient are not in the same physical location during the consent process. The key elements and guiding principles of the remote informed consent process are the same as with an in person visit. A copy of the written informed consent form is provided to the patient in advance or at the start of the enrollment visit. The informed consent process may be conducted over the phone or via video conference (e.g. FaceTime, Zoom) with the agreement of the patient. Each site will include standard procedures on remote consenting that is consistent with their institutional policies, in the MOP.

13.4.1 Before the remote visit

The research staff and patient will schedule a mutually agreeable time to review the informed consent and answer any questions about the study. To maintain privacy, the time should account for when both parties are able to join from a private setting. The informed consent discussion may be conducted

remotely over the phone or video conference (using a platform that is allowed by the site/institution). Research staff will share the IRB-approved informed consent form by email (including through DocuSign).

13.4.2 Procedures

The researcher and patient will have a copy of the approved consent document in view -- either a hard copy or digital copy open on a device -- at their respective locations. At the time of the informed consent discussion, the research staff will document how they verified the identity of the individual completing the remote process and signing the informed consent. The patient will be asked to present a photo ID to the research staff (or by an alternate site-specific method per the MOP).

Research staff will explain the procedures and tests involved in this study, and the associated risks, discomfort and benefits. Patients will have as much time as is needed to review the document and ask questions. No research procedures should commence until the researcher has received documentation of consent.

13.4.3 Documentation of process

The remote and in person consent processes will be documented on the Informed Consent Process Documentation form. Research staff will record the document signed, version/date number, language of consent, date and time, if the process was completed over in person/phone/video, and if the signature was hard copy or an electronic signature. The research staff obtaining consent will confirm that the Informed Consent Form, HIPAA Authorization and related study documents were thoroughly reviewed, the patient was given adequate time to review the documents and ask questions, and a summary of the discussion and patient's understanding of the information was presented.

13.4.4 Documentation of signature

Remote consent will be documented with required signatures on paper or by electronic signature.

Paper: patient will print out, sign and date a copy of the approved informed consent form, then send a scan or image (fax or photo) of the signed signature sheets to the research team; OR

Electronic: the digital document may be signed using a signing tool (e.g. DocuSign, Adobe Sign), as specified by the site's institutional guidelines.

13.4.5 Remote HIPAA authorization

When accessing, using, or disclosing PHI, HIPAA authorization is required. The HIPAA authorization language is currently embedded in the informed consent document and process.

13.5 Exclusion of Women, Minorities, and Children (Special Populations)

We are not proposing exclusion of any sex/gender or racial/ethnic group. This study is open to all eligible patients who meet study inclusion criteria. We will actively promote recruitment of a diverse group of patients, as well as patients from rural areas.

There will be no involvement of special classes, such as fetuses, neonates, pregnant or breastfeeding women, children under 18 years, prisoners, institutionalized individuals or populations who may be considered vulnerable. We will exclude children under 18 years of age in this trial because ARS affects children more rarely than adults and sinuses develop at different ages.

13.6 Patient Confidentiality

During the informed consent process, patients will authorize the use and disclosure of their personal health information. The informed consent form describes which study records may be shared, which parties may access these records and for what purposes, how the data are coded and/or de-identified, and the precautions that will be taken to maintain the confidentiality of their data.

The data manager, regulatory coordinator, study monitor, or other authorized representative acting as such, may access clinical trial documentation in order to ensure the protection of the rights, safety and well-being of individuals participating in clinical research.

The data forms capture the minimal amount of identifiable information needed to conduct the study. Contact information is maintained separately from the research database and access is limited to the immediate research team. Patients have a choice to opt-out of allowing their geographic information to be used for research on social determinants of health

The DCC will utilize The Research Electronic Data Capture (REDCap) to support the data management tasks such as data entry, random assignments to treatment groups, adverse event reporting, and data audit and query activities. This application provides electronic case report form creation and implementation, individual logins for application users, with security settings that are specific to each study the user can access.

The DCC has implemented Server and MySQL database management systems to provide structured repositories. DCC staff has extensive structured query language development expertise and will be able to store, report, and export research data as needed by statisticians, investigators, funder, or others. Study data will be stored on secure servers with access to this data protected by two-factor authentication.

13.7 Study Discontinuation

See Section 5.2.5. In the event that the study is discontinued, the current study enrollees will be advised that the research period has ended, and if necessary, to contact their provider regarding further treatment.

14 DATA HANDLING AND RECORD KEEPING

14.1 Data Management Responsibilities

The Data Coordinating Center (DCC) from the Center for Biostatistics, Informatics, and Data Science (CBIDS) at MedStar Health Research Institute will be the lead for data management on this study. Data management refers to the development of data collection tools and activities related to receiving, editing, updating, storing, and retrieving data for the study. The goal of data management efforts is high quality data that can be used to follow the analysis plan. Data management activities require a great deal of cooperation and coordination of activities between personnel at each site and the DCC. Data management activities will follow the data management plan developed during feasibility and shared with the team.

14.2 Data Capture Methods

The DCC will utilize REDCap to support the data management tasks such as data entry, random assignments to treatment groups, adverse event reporting, and data audit and query activities. The REDCap application will be used to develop the study CRFs, specifically the daily diary, mSNOT-16, Work Productivity and Activity Impairment Questionnaire, Patient Global Rating of Change Scale. The DCC has developed a Data Management and Site Quality Plan to document the procedures used for the collection, storage and management of all data for the trial. The application will allow for remote data capture. The data management plan will describe the data being collected, the methods for data entry, tracking study patients and specimens, data quality and security measures implemented, and reports for reviewing data from any of the study sites. An electronic audit trail of changes to database contents will be incorporated into the design and will capture and record those changes automatically.

14.3 Types of Data

Sources of data include clinical research forms (baseline health, adverse event, Follow-up Form, mSNOT-16, Work Productivity and Activity Impairment Questionnaire, Patient Global Rating of Change Scale) and self-reported diaries from patients and by interview with research personnel. Material or data gathered will be used strictly for research purposes. Use will not be made of existing specimens, records or data.

14.4 Timing/Reports

14.4.1 Quality control (QC) reports

Quality control reports are site specific and are sent to the study coordinator by email and copied to the study PI on a monthly basis. Copies also go to the DCC study file. Queries will be reported in MS Excel format or in the built in query system within the database. The DCC requires a response to each query within two weeks. The QC reports will also include an inventory of visits completed and missed by patients. This will provide guidance to study personnel about the site's performance, but will also prompt clinic coordinators to keep up to date with data entry and remind them to attempt rescheduling of missed visits.

14.4.2 Performance reports

Performance reports are sent to sites on a monthly basis. These detail site-specific and study-wide performance trends (i.e. enrollment status compared to enrollment goals, and rates of missed visits, lost to follow-ups, and missing data). Clinic personnel, investigators and the PI can use these reports to gauge study performance. At study group meetings, the DCC will present performance report results to elicit discussion on site-specific and study-wide improvement needs. Performance reports are used to elucidate differences between clinical sites and to foster efforts to keep the study on projected targets and to ensure maintenance of quality and high performance.

14.5 Study Records Retention

Study documents will be retained for a minimum of seven (7) years following IRB approval of study closure. These documents may be retained for a longer period, however, if required by local regulations or determined necessary by PCORI or IRBs. Paper records will be shredded and disposed of.

14.6 Protocol Deviations

A protocol deviation is any noncompliance with the clinical trial protocol or GCP requirements. The noncompliance may be either on the part of the study patient, the investigator, or the study personnel. As a result of deviations, corrective actions are to be developed by the site and implemented promptly. It is the responsibility of the study personnel to use continuous vigilance to identify protocol deviations. All deviations from the protocol must be addressed in the Protocol Deviation Log (REDCap site regulatory binder) and study record, if applicable. Major protocol deviations must be sent to the sIRB (and local IRB, if required) per their guidelines. The PI and study personnel are responsible for knowing and adhering to their ethics/IRB requirements. See reporting requirements and timelines in section 9.5.1.

Noncompliance to the protocol may also be on the part of the patient upon their clinician's recommendation and should be captured on the log when possible. As an example, noncompliance could include a situation where a patient does not take their assigned study drug because they revisited their clinician due to lack of symptom improvement and started antibiotics.

14.6.1 Major and minor protocol deviations

Major Protocol Deviation: A deviation that affects patient safety, rights, welfare, or data integrity.

Examples of major protocol deviations include (but are not limited to):
• Failure to obtain informed consent (i.e., no documentation of informed consent, consent obtained after study procedures were initiated)
• Enrolling a patient who does not meet inclusion/exclusion criteria
• Use of study procedures not approved by the IRB
• Failure to report serious adverse events to the IRB (per applicable requirements)
• Error in dispensing or dosing of drug/study medication, whether committed by patient or study team

• Failure to follow safety monitoring plan
• Enrollment after IRB-approval of study expired

Minor Protocol Deviation: A deviation that does not affect patient **safety, rights, welfare, or data integrity.**

Examples of minor protocol deviations include (but are not limited to):
• Inappropriate documentation of informed consent, including:
  • Copy not given to the person signing the consent form
  • Someone other than the patient dated the consent form
  • Expired consent used, but the version letter is identical to the currently approved consent form.
• Deviations from the approved study procedure that do not affect safety or data integrity
• Study procedure conducted out of sequence
• Omitting an approved portion of the protocol

15 DATA SHARING

The DCC and CCC will develop, maintain, and implement a plan that addresses the management, retention, and sharing of research data in a manner that is consistent with PCORI's Policy for Data Management and Data Sharing. The IRB-approved informed consent form permits data collected as part of this research project to be de-identified and shared with researchers not affiliated with the original project for appropriate secondary research purposes.

15.1 Funding Acknowledgement and Disclaimer

Publications/manuscripts, conference abstracts, poster presentations, and lay media coverage by any member of the research team, including patient and stakeholder partners, must contain the following:

Funding Acknowledgement: "This work was supported through a Patient-Centered Outcomes Research Institute (PCORI) Project Program Award (1234-56789)."

Disclaimer: "All statements in this report, including its findings and conclusions, are solely those of the authors and do not necessarily represent the views of the Patient-Centered Outcomes Research Institute (PCORI), its Board of Governors or Methodology Committee."

15.2 Return of Research Results to Study Patients

The overarching goal of this project is to help stakeholders from across the healthcare community make better-informed decisions on the treatment and management of acute rhinosinusitis. One aspect of this effort will be to provide stakeholders with results from the study which may help inform these decisions. We endorse the sharing of research findings with study patients as an important part of this process, and also recognize how essential their contributions are to the success of the study.

The lay summary could include both the context of the research and the results of the research conducted. The PAB, along with the input from the SAC and DEC, will have significant involvement in the development of the lay summary for both community stakeholders and the study patients. We will solicit input and feedback on the type of content that would be most meaningful to patients, language, format and channel of dissemination, as well as help with crafting and reviewing the final messages and materials.

With input from the PAB and SAC, the DCC will work on the format and delivery method(s) for sharing results for the different audiences. Possible modalities include: email or other electronic delivery, HIPAA compliant file-sharing service (e.g. BOX), password-protected study website, webinar or other group event, and print materials. The formats will be simple and concise, which may include infographics, one-pagers, videos, presentations, and/or social media. A summary tailored for healthcare professionals, researchers, and participating clinics will include a summary of the overall results.

With any format or channel, measures will be implemented to ensure that patients are not individually identifiable. All research data collected in this study is entered with an alphanumeric code, which will be removed before analysis. Additionally, the DCC will implement a final de-identification procedure of the data prior to sharing.

16 REFERENCES

1. Lemiengre MB, van Driel ML, Merenstein D, Liira H, Mäkelä M, De Sutter AI. Antibiotics for acute rhinosinusitis in adults. *Cochrane Database Syst Rev*. 2018;9:CD006089. doi:10.1002/14651858.CD006089.pub5
2. Smith SS, Evans CT, Tan BK, Chandra RK, Smith SB, Kern RC. National burden of antibiotic use for adult rhinosinusitis. *J Allergy Clin Immunol*. 2013;132(5):1230-1232. doi:10.1016/j.jaci.2013.07.009
3. Group GSW. The "top 5" lists in primary care: meeting the responsibility of professionalism. *Arch Intern Med*. 2011;171(15):1385-1390.
4. Fairlie T, Shapiro DJ, Hersh AL, Hicks LA. National Trends in Visit Rates and Antibiotic Prescribing for Adults With Acute Sinusitis. *Arch Intern Med*. 2012;172(19):1513.
5. Smith SR, Montgomery LG, Williams JW. Treatment of mild to moderate sinusitis. *Arch Intern Med*. 2012;172(6):510-513. doi:10.1001/archinternmed.2012.253
6. U.S. Action to Combat Antibiotic Resistance. Published 2019. Accessed June 29, 2020. https://www.cdc.gov/drugresistance/us-activities.html
7. Anon JB, Jacobs MR, Poole MD, et al. Antimicrobial treatment guidelines for acute bacterial rhinosinusitis. *Otolaryngol--Head Neck Surg Off J Am Acad Otolaryngol-Head Neck Surg*. 2004;130(1 Suppl):1-45. doi:10.1016/j.otohns.2003.12.003
8. Battisti AS, Modi P, Pangia J. Sinusitis. In: *StatPearls*. StatPearls Publishing; 2020.
9. Madaras-Kelly K, Hostler C, Townsend M, et al. Impact of Implementation of the Core Elements of Outpatient Antibiotic Stewardship Within Veterans Health Administration Emergency Departments and Primary Care Clinics on Antibiotic Prescribing and Patient Outcomes. *Clin Infect Dis Off Publ Infect Dis Soc Am*. 2021;73(5):e1126-e1134. doi:10.1093/cid/ciaa1831
10. Rovelsky SA, Remington RE, Nevers M, et al. Comparative effectiveness of amoxicillin versus amoxicillin-clavulanate among adults with acute sinusitis in emergency department and urgent care settings. *J Am Coll Emerg Physicians Open*. 2021;2(3):e12465. doi:10.1002/emp2.12465
11. Tsay S. Pending publication of data using MarketScan Commercial Dataset, from the Outpatient Stewardship Lead in the Office of Antibiotic Stewardship, Division of Healthcare Quality Promotion, Centers for Disease Control and Prevention. Published online September 20, 2021.
12. Sng WJ, Wang DY. Efficacy and side effects of antibiotics in the treatment of acute rhinosinusitis: a systematic review. *Rhinology*. 2015;53(1):3-9. doi:10.4193/Rhino13.225
13. Young J, De Sutter A, Merenstein D, et al. Antibiotics for adults with clinically diagnosed acute rhinosinusitis: a meta-analysis of individual patient data. *Lancet*. 2008;371(9616):908-914.
14. Hickner JM, Bartlett JG, Besser RE, Gonzales R, Hoffman JR, Sande MA. Principles of appropriate antibiotic use for acute rhinosinusitis in adults: background. *Ann Intern Med*. 2001;134(6):498-505.
15. Lindbaek M, Hjortdahl P, Johnsen UL. Use of symptoms, signs, and blood tests to diagnose acute sinus infections in primary care: comparison with computed tomography. *Fam Med*. 1996;28(3):183-188.
16. Ebell MH, McKay B, Guilbault R, Ermias Y. Diagnosis of acute rhinosinusitis in primary care: a systematic review of test accuracy. *Br J Gen Pract J R Coll Gen Pract*. 2016;66(650):e612-632. doi:10.3399/bjgp16X686581
17. Ebell MH, McKay B, Dale A, Guilbault R, Ermias Y. Accuracy of Signs and Symptoms for the Diagnosis of Acute Rhinosinusitis and Acute Bacterial Rhinosinusitis. *Ann Fam Med*. 2019;17(2):164-172. doi:10.1370/afm.2354
18. Ebell MH, Hansen JG. Proposed Clinical Decision Rules to Diagnose Acute Rhinosinusitis Among Adults in Primary Care. *Ann Fam Med*. 2017;15(4):347-354. doi:10.1370/afm.2060
19. Dale AP, Marchello C, Ebell MH. Clinical gestalt to diagnose pneumonia, sinusitis, and pharyngitis: a meta-analysis. *Br J Gen Pract J R Coll Gen Pract*. 2019;69(684):e444-e453. doi:10.3399/bjgp19X704297

20. Albrich WC, Monnet DL, Harbarth S. Antibiotic selection pressure and resistance in Streptococcus pneumoniae and Streptococcus pyogenes. *Emerg Infect Dis*. 2004;10(3):514-517.
21. Centers for Disease Control and Prevention and The Pew Charitable Trusts. Planning for a national goal for reduction in outpatient antibiotic use. In: ; 2015.
22. Goossens H, Ferech M, Vander Stichele R, Elseviers M. Outpatient antibiotic use in Europe and association with resistance: a cross-national database study. *Lancet*. 2005;365(9459):579-587.
23. Merenstein D, Gonzalez J, Young AG, Roberts RF, Sanders ME, Petterson S. Study to investigate the potential of probiotics in children attending school. *Eur J Clin Nutr*. 2011;65(4):447-453. doi:ejcn2010290 [pii] 10.1038/ejcn.2010.290
24. JL Avorn, JF Barrett, PG Davey, SA McEwen, TF O'Brien, SB Levy. Antibiotic resistance: synthesis of recommendations by expert policy groups. *Alliance Prudent Use Antibiot World Health Organ*. Published online 2001.
25. Fleming-Dutra KE, Hersh AL, Shapiro DJ, et al. Prevalence of Inappropriate Antibiotic Prescriptions Among US Ambulatory Care Visits, 2010-2011. *JAMA*. 2016;315(17):1864.
26. Merenstein D, Fraser CM, Roberts RF, et al. Bifidobacterium animalis subsp. lactis BB-12 Protects against Antibiotic-Induced Functional and Compositional Changes in Human Fecal Microbiome. *Nutrients*. 2021;13(8):2814. doi:10.3390/nu13082814
27. Zhang X, Borbet TC, Fallegger A, Wipperman MF, Blaser MJ, Müller A. An Antibiotic-Impacted Microbiota Compromises the Development of Colonic Regulatory T Cells and Predisposes to Dysregulated Immune Responses. *mBio*. 2021;12(1):e03335-20. doi:10.1128/mBio.03335-20
28. Roubaud-Baudron C, Ruiz VE, Swan AM, et al. Long-Term Effects of Early-Life Antibiotic Exposure on Resistance to Subsequent Bacterial Infection. *mBio*. 2019;10(6):e02820-19. doi:10.1128/mBio.02820-19
29. Guo J, Han X, Huang W, You Y, Zhan J. Gut dysbiosis during early life: causes, health outcomes, and amelioration via dietary intervention. *Crit Rev Food Sci Nutr*. Published online April 28, 2021:1-23. doi:10.1080/10408398.2021.1912706
30. Guirro M, Costa A, Gual-Grau A, et al. Effects from diet-induced gut microbiota dysbiosis and obesity can be ameliorated by fecal microbiota transplantation: A multiomics approach. *PloS One*. 2019;14(9):e0218143. doi:10.1371/journal.pone.0218143
31. Centers for Disease Control and Prevention. Adverse Drug Events from Specific Medicines. Accessed December 21, 2020. https://www.cdc.gov/medicationsafety/adverse-drug-events-specific-medicines.html
32. Lemiengre MB, van Driel ML, Merenstein D, Young J, De Sutter AI. Antibiotics for clinically diagnosed acute rhinosinusitis in adults. *Cochrane Database Syst Rev*. 2012;10:CD006089. doi:10.1002/14651858.CD006089.pub4
33. Hauer AJ, Luiten EL, van Erp NF, et al. No Evidence for Distinguishing Bacterial from Viral Acute Rhinosinusitis Using Fever and Facial/Dental Pain: A Systematic Review of the Evidence Base. *Otolaryngol Neck Surg*. 2014;150(1):28-33. doi:10.1177/0194599813510891
34. van den Broek MFM, Gudden C, Kluijfhout WP, et al. No evidence for distinguishing bacterial from viral acute rhinosinusitis using symptom duration and purulent rhinorrhea: a systematic review of the evidence base. *Otolaryngol--Head Neck Surg Off J Am Acad Otolaryngol-Head Neck Surg*. 2014;150(4):533-537. doi:10.1177/0194599814522595
35. Briel M, Schuetz P, Mueller B, et al. Procalcitonin-guided antibiotic use vs a standard approach for acute respiratory tract infections in primary care. *Arch Intern Med*. 2008;168(18):2000-2007.
36. Stalman W, van Essen GA, van der Graaf Y, de Melker RA. The end of antibiotic treatment in adults with acute sinusitis-like complaints in general practice? A placebo-controlled double-blind randomized doxycycline trial. *Br J Gen Pract J R Coll Gen Pract*. 1997;47(425):794-799.
37. Kaiser L, Lew D, Hirschel B, et al. Effects of antibiotic treatment in the subset of common-cold patients who have bacteria in nasopharyngeal secretions. *Lancet Lond Engl*. 1996;347(9014):1507-

1510. doi:10.1016/s0140-6736(96)90670-4
38. Bucher HC, Tschudi P, Young J, et al. Effect of amoxicillin-clavulanate in clinically diagnosed acute rhinosinusitis: a placebo-controlled, double-blind, randomized trial in general practice. *Arch Intern Med*. 2003;163(15):1793-1798.
39. Merenstein D, Whittaker C, Chadwell T, Wegner B, D'Amico F. Are antibiotics beneficial for patients with sinusitis complaints? A randomized double-blind clinical trial. *J Fam Pr*. 2005;54(2):144-151. doi:jfp_0205_5402j [pii]
40. Williamson IG, Rumsby K, Benge S, et al. Antibiotics and topical nasal steroid for treatment of acute maxillary sinusitis: a randomized controlled trial. *JAMA*. 2007;298(21):2487-2496.
41. De Sutter AI, De Meyere MJ, Christiaens TC, Van Driel ML, Peersman W, De Maeseneer JM. Does amoxicillin improve outcomes in patients with purulent rhinorrhea? A pragmatic randomized double-blind controlled trial in family practice. *J Fam Pract*. 2002;51(4):317-323.
42. Meltzer EO, Bachert C, Staudinger H. Treating acute rhinosinusitis: comparing efficacy and safety of mometasone furoate nasal spray, amoxicillin, and placebo. *J Allergy Clin Immunol*. 2005;116(6):1289-1295. doi:10.1016/j.jaci.2005.08.044
43. Varonen H, Kunnamo I, Savolainen S, et al. Treatment of acute rhinosinusitis diagnosed by clinical criteria or ultrasound in primary care. A placebo-controlled randomised trial. *Scand J Prim Health Care*. 2003;21(2):121-126. doi:10.1080/02813430310001743
44. Fokkens WJ, Lund VJ, Mullol J, et al. EPOS 2012: European position paper on rhinosinusitis and nasal polyps 2012. A summary for otorhinolaryngologists. *Rhinology*. 2012;50(1):1-12. doi:10.4193/Rhin
45. Rosenfeld RM, Piccirillo JF, Chandrasekhar SS, et al. Clinical practice guideline (update): adult sinusitis. *Otolaryngol--Head Neck Surg Off J Am Acad Otolaryngol-Head Neck Surg*. 2015;152(2 Suppl):S1-S39. doi:10.1177/0194599815572097
46. de Ferranti SD, Ioannidis JP, Lau J, Anninger WV, Barza M. Are amoxycillin and folate inhibitors as effective as other antibiotics for acute sinusitis? A meta-analysis. *BMJ*. 1998;317(7159):632-637.
47. Arroll B. Non-antibiotic treatments for upper-respiratory tract infections (common cold). *Respir Med*. 2005;99(12):1477-1484.
48. Falagas ME, Giannopoulou KP, Vardakas KZ, Dimopoulos G, Karageorgopoulos DE. Comparison of antibiotics with placebo for treatment of acute sinusitis: a meta-analysis of randomised controlled trials. *Lancet Infect Dis*. 2008;8(9):543-552.
49. Lindbaek M, Hjortdahl P, Johnsen UL. Randomised, double blind, placebo controlled trial of penicillin V and amoxycillin in treatment of acute sinus infections in adults. *BMJ*. 1996;313(7053):325-329.
50. van Buchem FL, Knottnerus JA, Schrijnemaekers VJJ, Peeters MF. Primary-Care-Based Randomized Placebo-Controlled Trial of Antibiotic Treatment in Acute Maxillary Sinusitis. *Lancet*. 1997;349:683-687.
51. Wald ER, Chiponis D, Ledesma-Medina J. Comparative effectiveness of amoxicillin and amoxicillin-clavulanate potassium in acute paranasal sinus infections in children: a double-blind, placebo-controlled trial. *Pediatrics*. 1986;77(6):795-800.
52. Axelsson A, Chidekel N, Grebelius N, Jensen C. Treatment of acute maxillary sinusitis. A comparison of four different methods. *Acta Otolaryngol*. 1970;70(1):71-76.
53. Gananca M, Trabulsi LR. The therapeutic effects of cyclacillin in acute sinusitis: in vitro and in vivo correlations in a placebo-controlled study. *Curr Med Res Opin*. 1973;1(6):362-368.
54. Garbutt JM, Banister C, Spitznagel E, Piccirillo JF. Amoxicillin for acute rhinosinusitis: a randomized controlled trial. *JAMA*. 2012;307(7):685-692. doi:10.1001/jama.2012.138
55. Psaltis AJ, Vyskocil E. What are the challenges in choosing pharmacotherapy for rhinosinusitis? *Expert Opin Pharmacother*. 2020;21(4):427-433. doi:10.1080/14656566.2020.1717467
56. Venekamp RP, Thompson MJ, Hayward G, et al. Systemic corticosteroids for acute sinusitis. *Cochrane Database Syst Rev*. 2014;(3):CD008115. doi:10.1002/14651858.CD008115.pub3

57. Zalmanovici Trestioreanu A, Yaphe J. Intranasal steroids for acute sinusitis. *Cochrane Database Syst Rev*. 2013;(12):CD005149. doi:10.1002/14651858.CD005149.pub4
58. Fokkens WJ, Lund VJ, Hopkins C, et al. European Position Paper on Rhinosinusitis and Nasal Polyps 2020. *Rhinology*. 2020;58(Suppl S29):1-464. doi:10.4193/Rhin20.600
59. Saline Nasal Irrigation for Sinus Problems. *Am Fam Physician*. 2009;80(10):1121-1122.
60. Rabago D, Zgierska A. Saline nasal irrigation for upper respiratory conditions. *Am Fam Physician*. 2009;80(10):1117-1119.
61. Chow AW, Benninger MS, Brook I, et al. IDSA Clinical Practice Guideline for Acute Bacterial Rhinosinusitis in Children and Adults. *Clin Infect Dis*. 2012;54(8):e72-e112.
62. Rabago D, Zgierska A, Mundt M, Barrett B, Bobula J, Maberry R. Efficacy of daily hypertonic saline nasal irrigation among patients with sinusitis: a randomized controlled trial. *J Fam Pract*. 2002;51(12):1049-1055.
63. Pynnonen MA, Mukerji SS, Kim HM, Adams ME, Terrell JE. Nasal saline for chronic sinonasal symptoms: a randomized controlled trial. *Arch Otolaryngol Head Neck Surg*. 2007;133(11):1115-1120. doi:10.1001/archotol.133.11.1115
64. Little P, Stuart B, Mullee M, et al. Effectiveness of steam inhalation and nasal irrigation for chronic or recurrent sinus symptoms in primary care: a pragmatic randomized controlled trial. *CMAJ*. 2016;188(13):940-949. doi:10.1503/cmaj.160362
65. Rabago D, Kille T, Mundt M, Obasi C. Results of a RCT assessing saline and xylitol nasal irrigation for CRS and fatigue in Gulf War illness. *Laryngoscope Investig Otolaryngol*. 2020;5(4):613-620. doi:10.1002/lio2.425
66. Rabago D, Zgierska A, Peppard P, Bamber A. The prescribing patterns of Wisconsin family physicians surrounding saline nasal irrigation for upper respiratory conditions. *WMJ*. 2009;108(3):145-150.
67. Keesara S, Jonas A, Schulman K. Covid-19 and Health Care's Digital Revolution. *N Engl J Med*. 2020;382(23):e82. doi:10.1056/NEJMp2005835
68. Shachar C, Engel J, Elwyn G. Implications for Telehealth in a Postpandemic Future: Regulatory and Privacy Issues. *JAMA*. 2020;323(23):2375-2376. doi:10.1001/jama.2020.7943
69. Shi Z, Mehrotra A, Gidengil CA, Poon SJ, Uscher-Pines L, Ray KN. Quality Of Care For Acute Respiratory Infections During Direct-To-Consumer Telemedicine Visits For Adults. *Health Aff Proj Hope*. 2018;37(12):2014-2023. doi:10.1377/hlthaff.2018.05091
70. Ray KN, Shi Z, Gidengil CA, Poon SJ, Uscher-Pines L, Mehrotra A. Antibiotic Prescribing During Pediatric Direct-to-Consumer Telemedicine Visits. *Pediatrics*. 2019;143(5):e20182491. doi:10.1542/peds.2018-2491
71. Garbutt J, Spitznagel E, Piccirillo J. Use of the modified SNOT-16 in primary care patients with clinically diagnosed acute rhinosinusitis. *Arch Otolaryngol Head Neck Surg*. 2011;137(8):792-797.
72. Piccirillo JF, Edwards D, Haiduk A, Yonan C, Thawley SE. Psychometric and Clinimetric Validity of the 31-Item Rhinosinusitis Outcome Measure (RSOM-31). *Am J Rhinol*. 1995;9(6):297-306.
73. Piccirillo JF, Merritt MG, Richards ML. Psychometric and Clinimetric Validity of the 20-Item Sino-Nasal Outcome Test (Snot-20). *Otolaryngol Neck Surg*. 2002;126(1):41-47.
74. National Institute of Mental Health (NIMH). Patient Global Impression scale (PGI). https://eprovide.mapi-trust.org/instruments/patient-global-impressions-scale-change-improvement-severity#basic_description
75. Rabago D, Barrett B, Marchand L, Maberry R, Mundt M. Qualitative aspects of nasal irrigation use by patients with chronic sinus disease in a multimethod study. *Ann Fam Med*. 2006;4(4):295-301. doi:10.1370/afm.552
76. Rabago D, Pasic T, Zgierska A, Barrett B, Mundt M, Maberry R. The efficacy of hypertonic saline nasal irrigation for chronic sinonasal symptoms. *Otolaryngol--Head Neck Surg Off J Am Acad Otolaryngol-Head Neck Surg*. 2005;133:3-8.
77. Hayer SD, Rabago DP, Amaza IP, et al. Effectiveness of nasal irrigation for chronic rhinosinusitis and

fatigue in patients with Gulf War illness: Protocol for a randomized controlled trial. *Contemp Clin Trials*. 2015;41:219-226. doi:10.1016/j.cct.2015.01.008

78. Is Rinsing Your Sinuses Safe? http://www.fda.gov/ForConsumers/ConsumerUpdates/ucm316375.htm
79. Stuart B, Hounkpatin H, Becque T, et al. Delayed antibiotic prescribing for respiratory tract infections: individual patient data meta-analysis. *BMJ*. Published online April 28, 2021:n808. doi:10.1136/bmj.n808
80. Westfall JM, Mold J, Fagnan L. Practice-based research--"Blue Highways" on the NIH roadmap. *JAMA*. 2007;297(4):403-406. doi:297/4/403 [pii] 10.1001/jama.297.4.403
81. Bowman MA, Neale AV. Journal of the American Board of Family Medicine Sixth Annual Practice-based Research Network Theme Issue -They Just Keep Getting Better and Better. *J Am Board Fam Med*. 2011;24(5):481-482.
82. Williams RL, Rhyne RL. No longer simply a Practice-based Research Network (PBRN) health improvement networks. *J Am Board Fam Med*. 2011;24(5):485-488. doi:24/5/485 [pii] 10.3122/jabfm.2011.05.110102
83. Grimshaw JM, Shirran L, Thomas R, et al. Changing provider behavior: an overview of systematic reviews of interventions. *Med Care*. 2001;39(8 Suppl 2):II2-45.
84. Tamariz L, Palacio A, Robert M, Marcus EN. Improving the informed consent process for research subjects with low literacy: a systematic review. *J Gen Intern Med*. 2013;28(1):121-126. doi:10.1007/s11606-012-2133-2
85. Cargill SS. How Do We Really Communicate? Challenging the Assumptions behind Informed Consent Interventions. *Ethics Hum Res*. 2019;41(4):23-30. doi:10.1002/eahr.500024
86. Peiris R, Cornell S, Greaves K, Bonner C. Do hospital consent forms for cardiology procedures meet health literacy standards? Evaluation of understandability and readability. *Patient Educ Couns*. Published online September 14, 2021:S0738-3991(21)00573-5. doi:10.1016/j.pec.2021.08.027
87. Bukini D, Mbekenga C, Nkya S, et al. A qualitative study on aspects of consent for genomic research in communities with low literacy. *BMC Med Ethics*. 2020;21(1):48. doi:10.1186/s12910-020-00488-0
88. Crossley JR, Tan TP, Smith KH, Ross JS, Merenstein DJ. Review of clinical trial settings of drugs commonly used in primary care and approved between 2005 and 2012. *J Gen Intern Med*. 2018;33(9):1431-1432. doi:10.1007/s11606-018-4486-7
89. Murphy M, Merenstein D. Grassroots campaign trail methods to recruit for clinical trials: recruitment lessons learned from trail to trial. *Clin Med Insights Pediatr*. 2011;5:1-7. doi:10.4137/CMPed.S6488
90. Cohen J. *Statistical Power Analysis for the Behavioral Sciences*. 0 ed. Routledge; 2013. doi:10.4324/9780203771587
91. Austin PC. Using the Standardized Difference to Compare the Prevalence of a Binary Variable Between Two Groups in Observational Research. *Commun Stat - Simul Comput*. 2009;38(6):1228-1234. doi:10.1080/03610910902859574
92. Yang D, Dalton JE. A Unified Approach to Measuring the Effect Size Between Two Groups Using SAS®, Orlando, FL: SAS Global Forum; 2012.

17 APPENDICES

Appendix A: Project Timeline
Appendix B: Saline Nasal Irrigation Instructions
Appendix C: Modified Sino-Nasal Outcome Test - 16 and Instructions for Scoring
Appendix D: Committee Charters
Appendix E: Data Safety Monitoring Board Charter
Appendix F: Informed Consent Form (Masterfile Template)
Appendix G: Data Collection Forms (Attachment)

APPENDIX A: Project Timeline

Full-Scale Study Phase

	2024		2025				2026				2027				2028				2029	
	Q3	4	1	2	3	4	1	2	3	4	1	2	3	4	1	2	3	4	1	2
Protocol	x																			
Engagement plan	x																			
Data collection forms	x																			
Recruitment materials	x																			
MOP	x																			
IRB submission		x																		
Contract amendments		x																		
Recruitment			x	x	x	x	x	x	x	x	x	x	x	x						
Data analysis														x	x	x	x			
Progress report		x		x		x		x		x		x		x		x		x		
Peer-review																		x	x	x

APPENDIX B: Saline Nasal Irrigation Instructions

Nasal Irrigation for Sinusitis Symptoms: Instructions for Tap Water Use

Nasal irrigation (rinsing the nose) is a commonly used way to treat sinus symptoms and infections. These instructions show how to make the solution and use your nasal wash system. Try to use it twice daily during the study. Regardless of how often you use it, please track your use within your daily diary.

Instructional video: Using SinuCleanse Micro-Filtered Nasal Wash System *(less than 2 minutes)*

1. What's in the Box?

- Soft Tip Nasal Wash System
- Product ready to use out of the box; no need for pre-wash.
- Premixed Saline Packets

2. Nasal Wash System Preparation

- Unwrap the blue cap.
- Pour the contents of the one (1) SinuCleanse natural saline solution packet into the bottle.

3. Nasal Wash System Preparation

- Fill the Nasal Wash bottle with warm tap water to the 8-oz mark indicated on the bottle.
- Screw the blue cap onto the bottle; ensure a firm connection.
- Gently shake the bottle until the dry ingredients have completely dissolved.

4. Nasal Wash System Use

- Turn the bottle upside-down so the comfort tip is facing up.
- Place the comfort tip at either nostril and gently insert the tip so that it forms a comfortable seal.
- Lean over the sink with your head facing down so you are looking into the basin.
- **As you breathe through your mouth**, squeeze the bottle gently so that the solution enters the nostril. In a few moments, the solution will begin to drain from the lower nostril.
- Continue to squeeze the bottle gently **until you have used approximately half of the solution.**

Please turn over for:

- Further instruction
- FAQs

5. Nasal Wash System Use<br><br>● Remove the comfort tip from your nostril, then exhale through both nostrils to clear them of excess mucus and solution.<br>● Gently blow your nose into a tissue.<br>● Repeat the procedure on the other nostril with the remaining solution.
6. Cleaning and Storage of Nasal Wash System<br><br>● Unscrew the bottle from the blue cap. Wash the bottle with mild soap, and set to dry.<br>● Unscrew the white filter from the blue cap, and set to dry. **Do not touch or rinse the filter**.<br>● Gently wash the blue cap and nozzle with mild soap, rinse, and set to dry.
7. Subsequent Nasal Wash System Use<br><br>● Continue to use as outlined above once daily; increase to 2 times daily if able.<br>● Increase to two (2) packets in the 8 oz bottle. If the more concentrated solution is uncomfortable, pour out the liquid and go back to one (1) packet per bottle.

Frequently Asked Questions:

When should I do nasal irrigation? *Any time of the day convenient for you. Try to use it twice daily.*

How frequently should I clean the Nasal Wash System? *Cleaning is recommended after each use.*

May I adjust the temperature of the tap water? *Yes, to room temperature or slightly warmer.*

I used the study nasal irrigation device but prefer my own device, can I use it? *Yes, but please note it on the log, and otherwise complete the log.*

I can't use high-volume nasal irrigation devices like this, can I use an OTC saline nasal spray? *Yes, but if you use such product, please specify in comments and try to use it at least 4 times daily, noting this on the log.*

The water does not pour out the other nostril. What should I do?

- *Make sure to breathe through your mouth while irrigating.*
- *Make sure you are not pressing the spout against the middle of your nose (septum).*
- *Be patient; sometimes it takes time for water to find its way through a congested nose. Hold the tip to your nostril for at least ten (10) seconds before removing it and trying it on the other nostril. Keep at it, even if water does not move through to the other nostril.*

Water in my nose is uncomfortable. What should I do? *If you are using one packet per bottle, be patient and try it a few times. Most people with sinus symptoms say they feel better after irrigating, even if the water does not go through. If you have increased to two (2) packets per bottle, reduce to one (1) packet per bottle.*

There is a vent knob near the screw top of the bottle. If it pops out, simply press it firmly back in place.

Nasal Irrigation for Sinusitis Symptoms: Instructions for Distilled Water Use

Nasal irrigation (rinsing the nose) is a commonly used way to treat sinus symptoms and infections. These instructions show how to make the solution and use your nasal wash system. Try to use it twice daily during the study. Regardless of how often you use it, please track your use within your daily diary.

Instructional video: Using SinuCleanse Micro-Filtered Nasal Wash System *(less than 2 minutes)*

1. What's in the Box?<br><br>● Soft Tip Nasal Wash System.<br>● Product ready to use out of the box; no need for pre-wash.<br>● Premixed Saline Packets
2. Nasal Wash System Preparation<br><br>● Unwrap the blue cap.<br>● Pour the contents of the one (1) SinuCleanse natural saline solution packet into the bottle.
3. Nasal Wash System Preparation<br><br>● Fill the Nasal Wash bottle with either study-provided, distilled water or distilled water of your choice to the 8-oz mark indicated on the bottle.<br>● Screw the blue cap onto the bottle; ensure a firm connection.<br>● Gently shake the bottle until the dry ingredients have completely dissolved.
4. Nasal Wash System Use<br><br>● Turn the bottle upside-down so the comfort tip is facing up.<br>● Place the comfort tip at either nostril and gently insert the tip so that it forms a comfortable seal.<br>● Lean over the sink with your head facing down so you are looking into the basin.<br>● **As you breathe through your mouth**, squeeze the bottle gently so that the solution enters the nostril. In a few moments, the solution will begin to drain from the lower nostril.<br>● Continue to squeeze the bottle gently **until you have used approximately half of the solution.**<br><br>**Please turn over for**:<br>● Further instruction<br>● FAQs
5. Nasal Wash System Use<br><br>● Remove the comfort tip from your nostril, then exhale through both nostrils to clear them of excess mucus and solution.<br>● Gently blow your nose into a tissue.<br>● Repeat the procedure on the other nostril with the remaining solution.

6. Cleaning and Storage of Nasal Wash System
• Unscrew the bottle from the blue cap. Wash the bottle with mild soap, and set to dry.<br>• Unscrew the white filter from the blue cap, and set to dry. **Do not touch or rinse the filter**.<br>• Gently wash the blue cap and nozzle with mild soap, rinse, and set to dry.
7. Subsequent Nasal Wash System Use
• Continue to use as outlined above once daily; increase to 2 times daily if able.<br>• Increase to two (2) packets in the 8 oz bottle. If the more concentrated solution is uncomfortable, pour out the liquid and go back to one (1) packet per bottle.

Frequently Asked Questions:

When should I do nasal irrigation? *Any time of the day convenient for you. Try to use it twice daily.*

How frequently should I clean the Nasal Wash System? *Cleaning is recommended after each use.*

May I adjust the temperature of the study-provided water? *Yes, to room temperature or slightly warmer.*

Distilled water should be kept in the refrigerator. Can I microwave it? *Yes, to room temperature or slightly warmer.*

I used the study nasal irrigation device but prefer my own device, can I use it? *Yes, but please note it on the log, and otherwise complete the log.*

I can't use high-volume nasal irrigation devices like this, can I use an OTC saline nasal spray? *Yes, but if you use such product, please specify in comments and try to use it at least 4 times daily, noting this on the log.*

The water does not pour out the other nostril. What should I do?

- *Make sure to breathe through your mouth while irrigating.*
- *Make sure you are not pressing the spout against the middle of your nose (septum).*
- *Be patient; sometimes it takes time for water to find its way through a congested nose. Hold the tip to your nostril for at least ten (10) seconds before removing it and trying it on the other nostril. Keep at it, even if water does not move through to the other nostril.*

Water in my nose is uncomfortable. What should I do? *If you are using one packet per bottle, be patient and try it a few times. Most people with sinus symptoms say they feel better after irrigating. If you have increased to two (2) packets per bottle, reduce to one (1) packet per bottle.*

There is a vent knob near the screw top of the bottle. If it pops out, simply press it firmly back in place.

APPENDIX C. Modified Sino-Nasal Outcome Test - 16 and Instructions for Scoring

Below you will find a list of symptoms and social/emotional consequences of your sinus problem. We would like to know more about these problems and would appreciate your answering the following questions to the best of your ability. There are no right or wrong answers, and only you can provide us with this information. Please rate your problems as they have been over the past 24 hours. Thank you for your participation. Do not hesitate to ask for assistance if necessary.

1. Considering how severe the problem is when you experience it and how frequently it happens, please rate each item below on how "bad" it is by circling the number that corresponds with how you feel using this scale: →	**No Problem**	**Mild or Slight Problem**	**Moderate Problem**	**Severe Problem**	**5 Most Important Items**
1. Need to blow nose	**0**	**1**	**2**	**3**	◯
2. Sneezing	**0**	**1**	**2**	**3**	◯
3. Runny nose	**0**	**1**	**2**	**3**	◯
4. Cough	**0**	**1**	**2**	**3**	◯
5. Post-nasal discharge	**0**	**1**	**2**	**3**	◯
6. Thick nasal discharge	**0**	**1**	**2**	**3**	◯
7. Ear fullness	**0**	**1**	**2**	**3**	◯
8. Headache	**0**	**1**	**2**	**3**	◯
9. Facial pain/pressure	**0**	**1**	**2**	**3**	◯
10. Wake up at night	**0**	**1**	**2**	**3**	◯
11. Lack of a good night's sleep	**0**	**1**	**2**	**3**	◯
12. Wake up tired	**0**	**1**	**2**	**3**	◯
13. Fatigue	**0**	**1**	**2**	**3**	◯
14. Reduced productivity	**0**	**1**	**2**	**3**	◯
15. Reduced concentration	**0**	**1**	**2**	**3**	◯
16. Frustrated/restless/irritable	**0**	**1**	**2**	**3**	◯

2. Please mark the most important items affecting your health (maximum of 5 items) ____________↑

Instructions for Scoring Modified SNOT-16[71]

Patient rates the severity of their condition on each of the 16 items using a 0-4 category rating system:

0 = Not present/no problem
1 = Mild or slight problem
2 = Moderate problem
3 = Severe problem

Next, the patient identifies the most important items to them and the items they hope will improve the most with treatment (up to a maximum of 5 items)

SNOT-16 Scoring:

1. The Total mSNOT-16 score is calculated as the mean item score for all 16 items
2. The possible range of mSNOT-16 score is 0-3, with higher scores indicating greater rhinosinusitis-related health burden
3. The mSNOT-16 Change Score is the difference between Pre-treatment and Post-treatment Total mSNOT-16 score.
4. Impact of treatment is assessed with the mSNOT-16 Change Score
5. A separate mSNOT-16 score and Change score is also calculated based on the items rated as Important

APPENDIX D: Committee Charters

－**DRAFT SAC CHARTER**－

The SAC is a diverse group of individuals and organizations with interest in acute rhinosinusitis and antibiotic stewardship.

Mission: The SAC will provide oversight, resolve conflicts, and provide direction as needed by the CRC, or other study committees. The SAC co-chairs will present and facilitate the scheduled meetings, and may convene ad hoc meetings as needed. Also at each SAC meeting, the PI (or a designee) from the CRC will bring forth questions or issues raised by the study team regarding protocol design, implementation, recruitment, and technical challenges. The SAC will provide support in the decisions that have been made by the study or committee members, or give additional suggestions that they believe will strengthen the study. All feedback will be relayed back to the CRC and/or relevant committee.

The PIs of this project have had a long and successful history of collaborating with each other and other investigators, and have resolved conflicts without needing external mediation. In the event that consensus cannot be reached between the involved parties, a formal request may be submitted to the SAC co-chairs (Drs. Ebell and Piccirillo), who will mediate any scientific disagreements and/or decide to raise the issue to the full SAC. The PIs agree to abide by the final recommendations and decisions made by either Drs. Ebell and Piccirillo, or the SAC.

Additionally, PCORI staff may also reach out to the SAC for input on study questions. If another study committee wishes to introduce an item to the SAC, they may notify the PI or designee of their request and be added to the agenda for the next upcoming SAC meeting. The SAC plans to meet 3-4 times per year.

－**DRAFT DEC CHARTER**－

Mission: In recognition of the critical role of dissemination and engagement, the dedicated DEC will function as an independent group of individuals and organizations with expertise in translational research, health communication, and media. The DEC is tasked with: (i) creating an ongoing dissemination and translation strategy, (ii) engaging multiple stakeholders in dissemination activities, and (iii) assessing the readiness of the findings to be disseminated.

Readily Adapted: This study, with its large sample size and powered subgroups, will address limitations that have previously prevented changes in care. We will work with government agencies, insurance companies, clinical practice networks, academies, industry groups and other stakeholders to disseminate findings. The DEC will develop a plan based on best practices and published evidence of effective knowledge transfer, translation methodologies and tool kits for clinician behavior change interventions, as well as social media strategies.

End Users: The DEC will identify the full complement of end users who have an interest in implementing study findings. However, we anticipate the following will be key constituents: patients, clinicians, insurance companies and national organizations (such as the CDC) and those related to frontline clinicians who routinely provide care to individuals with ARS (such as American Academy of Family Physicians).

Potential Barriers: We view the issue of clinician uptake and patient acceptance of new recommendations as the main barrier to implementing our findings and translating them into practice. We will address these known barriers through use of multiple dissemination modalities, including via published research, conference presentations, enlisting trusted opinion leaders from their specialty, as well as through communications from professional guidelines. Having clinician and patient input at all stages of the project will help greatly in implementation of findings, as well as understanding potential facilitators and barriers to implementation from multiple perspectives. Additionally, we will work with toolkits developed through the Agency for Healthcare Research and Quality and the Canadian Institute for Health Research to guide our translation efforts. Patient expectation for antibiotics for ARS is an important issue, yet our qualitative research conducted on this area from our PBRNs/sites indicates that these are not fixed and patients are willing to avoid antibiotics when they are given more information and actively involved in these decisions. Previous work with otitis media has demonstrated that involving parents in decision-making is a major factor in decreasing antibiotic use. We believe that having an active PAB will help us properly address the issue of antibiotic expectations.

Resources: One of the DEC's missions is to help the CRC identify resources to support dissemination activities and provide feedback on communication plans. We anticipate traditional and online communication approaches to maximize diffusion of our findings and encourage stakeholders to act on and implement the resulting practice recommendations in their respective circles and communities.

APPENDIX E: Data Safety Monitoring Board Charter

1.0 Purpose

The purpose of this charter is to define the responsibilities of this Data Safety Monitoring Board (DSMB), its membership, timing of meetings, and procedures used to carry out these responsibilities.

2.0 DSMB Organization

The DSMB is an independent panel of experts which will periodically review and advise on the safety and rights of human research patients enrolled in this multi-center clinical trial. The DSMB will assess and provide unbiased recommendations of the ongoing scientific validity of the study, exposure to undue risk, and adverse event reports.

This DSMB is composed of a group of seven (7) experts or patient representatives with experience in the conduct of clinical research, biostatistics, and/or treatment of acute rhinosinusitis. The DSMB is wholly independent of the research team and has no direct involvement in the conduct of the study. The board will be chaired by Dr. Dan Tancredi, a statistician with expertise in clinical trials and infectious disease.

3.0 DSMB Responsibilities

A. Responsibilities

The DSMB will provide recommendations about stopping or continuing the clinical trial. The DSMB may provide recommendations relating to the selection, recruitment and retention of patients, improving adherence to the protocol and interventions, and procedures for data management and quality control.

A DSMB plan for the full-scale study was finalized during the feasibility period. The current planned reviews of safety data will occur after 10%, 20%, 40% and 70% data completion. Meetings will include a review of AE and SAE, including their occurrence, grading, expectedness, and relationship to study participation. In the event of an unanticipated problem or serious adverse event that is related or possibly related to participation in the research, a meeting may be convened ad hoc.

B. Members

DSMB members will:

- Review and provide feedback on the study-related documents in preparation of the full-scale study.
- Attend the pilot meeting.
- Review new information that could possibly increase the risk of harm to patients, or jeopardize the feasibility of the protocol.
- Reviewing safety data, in the case of an unanticipated problem or serious adverse event or unanticipated problem that is related or possibly related to participation in the research, as determined by the Chair.

C. Chair

In addition to the responsibilities listed in Section B, the DSMB Chair will:

- Review agendas for upcoming meetings, prepared by the research team.
- Lead and moderate meeting discussions.

- Assess the need for (and convene) an ad hoc meeting with the DSMB, in the event of a serious and unanticipated issue.
- Ensure a decision is reached at the conclusion of each meeting regarding the direction/continuation of the study.
- Review meeting minutes prepared by the research team.

D. Administrator

The DSMB Administrator will:

- Prepare and distribute agenda, reports prepared by the DCC, or other information to be reviewed by the DSMB.
- Arrange meeting logistics.
- Prepare minutes and submit to the Chair for review.
- Maintain cloud storage, sharing and user access of reports, forms, and other documentation.
- Coordinate stipend payments to members with the relevant University departments.

E. Data Coordinating Center

The DCC biostatistician and data manager will:

- Prepare performance and safety reports to facilitate the oversight role of the DSMB.

4.0 Meeting Structure

Prior to the initial meeting, the DSMB will be provided with the charter, protocol, informed consent form, data collection instruments, and other materials. During the initial meeting the PI will provide an overview of the study and the group will discuss the role and function of the DSMB, the format and content of future reports, and review the protocol, analysis plan, and data collection forms for the full-scale study. All meetings will be held remotely via Zoom.

In the event of an unanticipated problem or serious adverse event that is related or possibly related to participation in the research, a meeting may be convened ad hoc, as determined by the DSMB Chair.

DSMB meetings may be open or closed session:

A. Open Session

Members of the DSMB, DCC and study team members may be present at the open session meetings.

B. Closed Session

The DSMB may request a closed session, where only DSMB members and an unmasked DCC member may be present. In this session, the DSMB may request to review unblinded information by the intervention group. At the end of a closed session, the DSMB will provide the study team with recommendations including the continuation/discontinuation of the study.

5.0 Reporting of Adverse Events and Stopping Rules

The DSMB will not be responsible for stopping the study. After looking at blinded or unblinded data, they can make suggestions to the PI that will be shared with PCORI staff.

A. <u>Adverse Event</u>

An AE refers to an untoward or unfavorable medical occurrence, including abnormal signs, symptom, or

disease, temporally associated with the patient's involvement in the research, whether or not considered related to participation in the research. This includes the onset of a new event not present at baseline, or worsening during the course of the study even if present at the study start.

i. Grading

Adverse events will be graded using NCI Common Terminology Criteria for Adverse Events (CTCAE) Version 5.0. The CTCAE displays Grades 1 through 5 with unique clinical descriptions of severity for each event based on this general guideline:

- Grade 1 Mild; asymptomatic or mild symptoms; clinical or diagnostic observations only; intervention not indicated.
- Grade 2 Moderate; minimal, local or noninvasive intervention indicated; limiting age-appropriate instrumental activities of daily living (ADL).
- Grade 3 Severe or medically significant but not immediately life-threatening; hospitalization or prolongation of hospitalization indicated; disabling; limiting self-care ADL.
- Grade 4 Life-threatening consequences; urgent intervention indicated.
- Grade 5 Death related to AE.

ii. Relationship to Study Participation

The relationship of the adverse events to study participation will be classified as:

- Not related
- Possibly related
- Definitely related

iii. Known Potential Risks

The most common, expected adverse events or risks include:

- Amoxicillin-clavulanate: nausea and/or vomiting, mild to severe diarrhea, upset stomach, skin rash
- Budesonide nasal spray: sneezing, dry/sore nose, or nosebleeds, dry/sore throat, unpleasant taste or smell
- Saline nasal irrigation: nasal irritation, stinging and nosebleeds
- Capillary blood draw (fingerstick): excessive bleeding, fainting or feeling light-headed, hematoma (blood accumulating under the skin), infection (a slight risk any time the skin is broken)
- Non-active control/placebo: symptoms may not improve or worsen (patients will be advised to follow-up with their health care clinician)

B. Serious Adverse Event

An event is considered serious if the AE:

- Results in death
- Is life-threatening, or places the patient at immediate risk of death at the time of the AE
- Causes or is likely to result in persistent or significant disability or incapacity
- Results in congenital anomalies or birth defects
- May, based on medical judgment, jeopardize the patient and may require medical intervention to prevent one of the outcomes listed.

C. Study Stopping Criteria

The DSMB may recommend study procedures be suspended or discontinued if:

- There is a patient death that the PI or IRB believes may be related to the study. The DSMB should give their opinion about such matters.
- A statistically or clinically significant number of of hospitalizations occur that may be related to the study.
- New information from outside studies reveal that it would be unethical to continue enrolling patients in the study.
- The IRB or PCORI decide that the study should be stopped.

D. Withdrawal of Individuals

The DSMB will review the individual stopping criteria as detailed in the study protocol and may recommend modifications if additional safety concerns arise from their continuing reviews.

Individuals may be withdrawn from the study or the intervention(s) may be discontinued if:

- A temporally associated SAE that is related to study participation, or for which the relationship with study participation cannot be ruled out.
- The PI and/or patient (in consultation with the patient's health care clinician, if necessary) determines the patient should not continue with study participation.

6.0 Confidentiality

All materials, discussions and proceedings of the DSMB are confidential. Members and other attendees in DSMB meetings are expected to maintain confidentiality.

APPENDIX F: Informed Consent Form (MasterFile Template)

BIOMEDICAL RESEARCH ALLIANCE OF NEW YORK LLC
[INSTITUTION]
STUDY INFORMATION AND INFORMED CONSENT FORM

Protocol Title: Nasal steroids, irrigation, oral antibiotics, and subgroup targeting for effective management of sinusitis
Protocol #: PLACER-2021C3-24476
Funder: Patient-Centered Outcomes Research Institute (PCORI)

Site Principal Investigator: __________
Institution: __________
Address: __________
Telephone: __________

KEY INFORMATION ABOUT THIS RESEARCH STUDY

You are being asked to take part in a research study because you have symptoms that could be caused by an acute sinus infection. Sometimes, sinus infections happen when you are getting over a cold and can cause pressure and pain in your face, stuffy nose and other symptoms. Other common symptoms are runny nose, changes to your sense of smell, fever, headache, bad smelling breath, extreme tiredness, ear pain or pressure, and/or dental pain.

The following table is a concise and focused presentation of key information to assist you in understanding why you might or might not want to participate in the research.

**Purpose**	Sinus infections are sometimes treated with oral antibiotics or steroid nose sprays, while some patients get better on their own. Patients will often start supportive care to ease symptoms and lower their discomfort. Sometimes this care is enough to help patients wait a few days to see if their infection clears up without needing antibiotics or steroid nose sprays. The purpose of this study is to see which specific groups of patients benefit more from which treatment or combination of treatments, and which improve with supportive care alone.
**Interventions**	The interventions you receive in this study depend on how long you have had symptoms and which group you are randomly assigned to These four groups include: 1) antibiotic (amoxicillin/clavulanate) alone; 2) antibiotic plus steroid nose spray (amoxicillin/clavulanate and budesonide); 3) placebo antibiotic plus steroid nose spray (budesonide); 4) placebo antibiotic alone.<br><br>Both are commonly used medications that are approved by the United States Food and Drug Administration (FDA). You will not receive any experimental or untested drugs as part of this study.<br><br>Instead of the amoxicillin/clavulanate, you may receive placebo ('dummy drug'), which is an inactive pill designed to look the same as the active amoxicillin/clavulanate. You will have an equal (50/50) chance of receiving the amoxicillin/clavulanate or placebo. You will not know which one you receive.
**Voluntary Participation**	Your decision to participate in this study is voluntary.

**Withdrawal**	If you decide to be in this study and then change your mind, you can leave the study at any time without penalty. If at any time, you feel like you are not improving and should not be in a placebo trial, please talk with the local research team or your clinician.
**Length of Participation**	The length of time you are in the study will vary depending on how long you have been experiencing symptoms, for up to 35 days. We anticipate most people will be in the study for about 20 days.
**Procedures**	When you first enroll in the study, if you have been experiencing symptoms for less than 10 days, you will be provided with saline nasal irrigation supplies and instructions regarding supportive care to help manage your symptoms. When you reach nine days of symptoms, we will contact you to see if you are feeling better. If your symptoms have not improved, you will be randomly assigned to one of four groups.<br><br>Similarly, if you have been experiencing symptoms for 10 or more days at enrollment, you will be randomly assigned to one of four groups on the same day, and be provided with saline nasal irrigation supplies and instructions regarding supportive care.<br><br>The main procedures in the study include:<br>● Taking oral amoxicillin/clavulanate 875 mg/125 mg OR oral placebo pill (non-active), twice daily for 7 days<br>● With or without a steroid nose spray, budesonide 32 mcg per spray, two (2) sprays per nostril, once daily<br>● Using saline nasal irrigation one to three times daily (optional)<br>● Using symptomatic care as recommended (optional)<br>● C-reactive protein test – a measure of inflammation in the body<br>● Completing surveys and diaries about your symptoms<br><br>The research team will explain which procedures are being done for research, and which would be done as part of your standard care even if you do not participate.
**Risks or discomforts**	This study will use approved medicines that have risks. The most common risks and discomforts include:<br>● For antibiotics: nausea and/or vomiting, diarrhea, upset stomach and mild skin rash.<br>● For steroid nose spray: nasal irritation, nosebleeds, headaches.<br>● For saline nasal irrigation (wash): nasal irritation, nasal stinging and nosebleeds.<br><br>The research team will explain the risks of this research to you before you decide about participating. For more information about risks, please ask the research team.

**Benefit**	There is no guarantee that you will receive direct benefit as a result of your participation in this study; however, some potential benefits include:<br>● Resolution of symptoms after nasal irrigation, steroid nose sprays, and/or antibiotic treatments<br>● Close monitoring of symptoms through the follow-up questionnaires and interviews<br>● An opportunity to benefit others by contributing to knowledge of sinusitis treatments
**Alternative to Study Participation**	There may be other options for treatment including creating a treatment plan with your clinician.
**Costs**	Qualified study participants will not have to pay for antibiotics, steroid nasal spray or saline nasal irrigation materials. You or your insurance company will have to pay for any visits to your treating clinician and/or symptomatic care purchased over-the-counter. You or your insurance company will be charged for continuing medical care and/or hospitalization that are not a part of the study.
**Confidentiality**	There are provisions in place by the study protocol and study site to help protect the privacy and confidentiality of your personal health information and study information.

This overview does not include all of the information you need to know before deciding whether or not to take part. Much additional detail is given in the full consent document, which can be found on the pages that follow. Be sure to review the rest of this consent form before deciding about participation.

INFORMED CONSENT FORM

This consent form explains the research study. Before you decide to be a part of this study, you need to know why the research is being done, what it will involve and the risks and benefits. You can ask the research team--made up of the study clinician(s) and other study staff-- to explain anything on this form. Please take time to read this form carefully. Feel free to discuss it with your relatives, friends and your primary care clinician. If you agree to take part in this research study, you must sign this consent form.

Who is funding this study?

This study is being funded by the Patient-Centered Outcomes Research Institute (PCORI). PCORI is providing funds to the [Institution] to conduct this study with [Principal Investigator] as the site principal investigator.

What is the purpose of this study?

The purpose of this study is to evaluate the effectiveness of an oral antibiotic (amoxicillin/clavulanate) and steroid nose spray (budesonide) for acute sinusitis patients who do not improve with supportive care alone. The study also aims to identify which patients benefit most from oral antibiotics versus steroid nasal spray. Finally, the study aims to identify patient subgroups that benefit from supportive care alone, without antibiotics or nasal spray.

NUMBER OF PARTICIPANTS AND LENGTH OF STUDY PARTICIPATION

3,720 people are expected to enroll in this study at 6 research sites in the United States.

Some people feel better by the time they reach their 9th day of symptoms and complete the study at this time. About 2,232 people are expected to still have symptoms and continue with the randomization

stage.

Your participation in this study is expected to last approximately 2-4 weeks. The enrollment meeting will be in person or over a computer/phone and subsequent meetings will be conducted remotely.

STUDY PROCEDURES

• **Enrollment & Baseline**: If you take part in this study, we will complete enrollment forms about yourself and your health. You will also be asked to provide a capillary sample, also known as a fingerstick or finger prick, which is a blood sample collected by pricking the skin with a small needle or lancet. The research staff member will collect approximately 2-4 drops of blood which will be immediately tested for C-reactive protein, a test that checks for inflammation.

If you choose to use saline nasal irrigation, you will be given a nasal wash bottle and pre-packaged over-the-counter salt packets. In case you have never used one, a nasal wash bottle is a device that is used to pour approximately 1 cup of 0.9 to 2% salt solution into your nose and helps to clear your sinuses. You will receive instructions on how to use the saline nasal irrigation supplies.

• **Randomization**: If you take part in this study, you may be "randomized" into one of the four study groups: 1) antibiotic alone; 2) antibiotic plus steroid nose spray; 3) placebo antibiotic plus steroid nose spray; 4) placebo antibiotic alone. Randomization means that you are put into a group by chance. It is like flipping a coin or rolling a die. Which group you are put in is done by a computer. Neither you nor the researchers will choose what group you will be in. You will have an **equal 25%** chance of being placed in any group.

- If you had symptoms for 10 or more days, you will be randomized right away.
- If you had symptoms for less than 10 days, we will contact you again when you reach 9 days of symptoms. If you are not feeling better, you will be randomized. Before randomization there will be a period where you just take supportive care (such as over-the-counter) medicines recommended by your clinician.
- If you are feeling better by day 9, complete the study, then become sick again (called "double sickening") within 7 days, you may return to the study and will be randomized right away
- If you have "double sickening," meaning you had symptoms for 5 days, felt better, and then have new or worsening symptoms, you will be randomized right away.

• **Study Medicines**: Since there are four study groups, about 558 patients will receive the active antibiotic alone, 558 patients will receive the active antibiotic *plus* a steroid nose spray, 558 patients will receive a placebo pill with no active medicine *plus* a steroid nose spray and 558 patients will receive a placebo pill alone. A placebo is an inactive substance made to look and taste like the active antibiotic. A placebo will be used to see if the antibiotic works better or is safer than not taking anything. Neither you nor the research team will know whether or not you receive an active or placebo antibiotic.

In this study, you will need to take the antibiotic or the placebo pill 2 times per day for 7 days. If you are assigned to a group using the nose spray, you will use two (2) sprays per nostril, once per day.

• **Daily Diary**: Throughout the study, you will be asked to log your symptoms and use of saline nasal irrigation and/or supportive care in a daily diary. You will receive instructions on filling out the daily diary. Filling out this diary should take no more than 2 minutes each day. After you are assigned to a group, you will be asked to take your pills and/or steroid nose spray as directed, and continue to log

your use and symptoms in your daily diary. After you complete the medicines on day 7, a research team member will ask you to take a photo of any leftover, unused study medicine or empty medicine bottles.

• **Follow-up Questionnaires and Interviews**: As a study participant, you will complete 6 follow-ups: 4 will occur before or during the 7 days you are taking study medicine, and 2 will occur during the week after you finish. You may complete the follow-up questionnaires online using the computer/phone, or with a member of the research team. These interviews should last about 3-5 minutes. You will not be required to spend any additional time on the study beyond the time it takes to fill in your diary, take your medicine(s), and participate in the follow-ups.

PARTICIPANT RESPONSIBILITIES
As a participant in this study, you will have certain responsibilities, including the following:
● Participate in all study visits or calls and, if needed, reschedule appointments as soon as possible
● Follow the instructions of the research team
● Take the study interventions as directed
● Tell the research team all medicines that you are taking and check with the study clinician or team before taking any new medicines. This includes any prescription and over-the-counter medicine, vitamins, and herbal supplements, such as those bought from the drug or grocery store, etc.)
● Tell the study staff any time you do not feel well or if you have any side effects

RISKS AND DISCOMFORTS
Initially, when you are enrolled in the study, you may use supportive care and/or saline washes. This is similar to what your clinician would normally recommend (standard of care). There is a risk that symptoms get worse and you feel sicker. You can call your clinician and the research team at any time and remove yourself from the study.
This study will use approved medicines that have known potential risks. These are:
● For the antibiotic (amoxicillin/clavulanate): nausea and/or vomiting, diarrhea, upset stomach and mild rash.
● For steroid nose spray (budesonide): nasal irritation, nosebleeds, headaches.
● For saline nasal irrigation (wash): nasal irritation, nasal stinging and nosebleeds.

Fingerstick blood draw is when the tip of the finger is pricked with a sharp needle or a lancet, and a few drops of blood are collected for testing. Some people find it easier to get blood from a fingerstick than others. Risks are very rare but may include:
● Excessive bleeding
● Fainting or feeling light-headed
● Infection (a slight risk any time the skin is broken)

There are risks to taking part in any research study, including ones that we cannot predict. The chances of this happening are very small. You are encouraged to discuss any questions about potential risks with the researchers and your primary care clinician.

NEW INFORMATION
You will be notified in a timely way if important new findings become known that may affect your willingness to continue in the study.

A description of this clinical trial is available on http://www.ClinicalTrials.gov, as required by U.S. Law. This Web site will not include information that can identify you. At most, the Web site will include a summary of the results. You can search this Web site at any time.

Results That May Affect Your Health

As the results obtained during the research study are for research purposes only and are not for medical diagnosis, you will not receive individual results. In some circumstances, if the study clinician learns information related to your health from the study procedures, the study clinician will discuss this information and your options with you.

BENEFITS

We cannot promise any benefit to you or others from your participation in this study. However, some patients who receive antibiotics, steroid nose spray, and/or nasal irrigation, may find their symptoms get better or go away. The study results may help people in the future.

ALTERNATIVES TO STUDY PARTICIPATION

You do not have to participate in this study to receive treatment for your condition. You may choose to have treatment for your symptoms only or no treatment at all. There may be other options for treatment, including creating a treatment plan with your clinician.

COSTS OF PARTICIPATION

You will be provided with study materials (antibiotic or a placebo, steroid nose spray, and nasal irrigation materials) at no charge to you. There are no costs for tests and procedures required only for this study.

You and/or your insurance company will be responsible for the costs of all items and services during the research study, which you would have received for your condition if you were not enrolled in this research study and/or that your clinician believes are medically necessary to treat you. These may include your visit co-pays, over-the-counter medicines, and tests ordered by your clinician. You should discuss possible costs of study participation with the study staff and/or your insurance company.

COMPENSATION FOR PARTICIPATION

Payments for your time and effort will be made according to the following schedule:

- $20 upon enrollment
- If you had 10 or more days of symptoms: $20 upon randomization; *or* If you had less than 10 days of symptoms: $20 at day E9 follow-up, when you reach 9 days of symptoms
- $40 at day R3 follow-up, 3 days of taking the study medicine
- $40 at day R7 follow-up, 7th and last day of taking the study medicine
- $40 at day R14 follow-up, 7 days after finishing the study medicine.

If you leave the study early, you will be paid only for visits you complete. [Your payment will be [payment method] at the completion of the study visit.

You will not receive payment of any kind for your information and specimens (even if identifiers are removed) or for any tests, treatments, products or other things of value that may result from this research study.

Please note that administrative personnel involved in processing your payments may be aware of your identity or Social Security Number, but will not have access to your health information.

COMPENSATION FOR INJURY

For medical emergencies, call 911. If you become ill or are hurt while you are in this study, contact your study clinician immediately. Your study clinician will assist you in obtaining appropriate medical care.

The costs of care caused by the properly performed study procedures and/or administration of study medicines will be billed to you, your insurance, or other third party. The funder, the [Institution], and the Biomedical Research Alliance of New York, have no program to pay for medical care for research-related injury.

No other compensation will be offered by the funder, the [Institution], or the Biomedical Research Alliance of New York, including for things such as lost wages or discomfort. You are not waiving any legal right to seek additional compensation through the courts by signing this form.

The Policy and Procedure for the [Institution] are as follows:
We will make every effort to prevent study-related injuries and illnesses. If you are injured or become ill while you are in the study and the illness or injury is due to your participation in this study, you will receive necessary medical care. The costs of this care will be charged to you or your third-party payor (e.g. your Health Insurer) in the usual manner and consistent with applicable laws. No funds have been set aside by the [Institution], or their affiliates, to repay you or compensate you for a study related injury or illness.

CONFIDENTIALITY
We are committed to respecting your privacy and to the extent allowed by law, every effort will be made to keep your personal information confidential. Study records, which identify you and the consent form signed by you, will be looked at by the research team including the data coordinating center (MedStar Health Research Institute). Organizations that may request to inspect and/or copy your research and medical records for quality assurance data analysis and other research related and operational or administrative purposes, include groups such as: the funder or the funder's representatives, FDA and other regulatory agencies, the Institutional Review Board (IRB), and the Biomedical Research Alliance of New York. While these parties are aware of the need to keep your information confidential, total confidentiality cannot be guaranteed.

The results of this study may be presented at meetings or in publications; however, you will not be named nor will any identifying information will be included in these presentations and/ or publications.

If you take part in this study, you will be assigned a Study ID, a unique code to help protect your privacy. Your study records will be labeled with this code that does not directly identify you. The study staff at each site keep the linking code between your name and study information separately in a secure location.

AUTHORIZATION TO USE AND DISCLOSE PERSONAL HEALTH INFORMATION
Federal regulations give you certain rights related to your health information. These include the right to know who will be able to get the information and why they may be able to get it. When choosing to take part in this study, you are giving us the permission to use or give out any health information including information that might identify you. The health information we may collect from you and use for this research includes:

- Research records
- Records about phone calls made as part of this research
- Records about your study visits
- Information obtained during this research about laboratory test results
- Past and present medical records
- Results from diagnostic and medical procedures including but not limited to X-rays, physical examinations and medical history

- Billing records
- Your personal information will be used to contact you in regards to this study. Addresses can help us learn more about your geographic area. Later, we will ask to use your full address for research purposes; however, you have a choice to opt-out.

Information about your health may be used and given to others by the study clinician and staff. They might see the research information during and after the study. Any research information shared with others will not contain your name or any other personal identifier unless it is necessary for review by such parties or is required by law or [Institution]'s policy. Information about you and your health which might identify you may be shared with:

- The U.S. Food and Drug Administration
- Department of Health and Human Services agencies, such as the National Institutes of Health and the Centers for Disease Control and Prevention
- Governmental agencies in other countries
- Biomedical Research Alliance of New York (BRANY)
- The Institutional Review Board
- Accrediting agencies
- Data safety monitoring boards
- Health insurers and payers
- The funder (including persons or companies that are working for or with the funder or are owned by the funder)
- Other individuals and organizations that analyze or use your information in connection with these research activities, including laboratories, administrative staff and study sites

Your personal health information may be further shared by the groups above. If shared by them, the information will no longer be covered by the U.S. federal privacy laws. However, these groups are committed to keeping your personal health information confidential. If you give permission to give your identifiable health information to a person or business, the information may no longer be protected. There is a risk that your information will be released to others without your permission.

Information about you and your health that might identify you may be given to others to carry out the research study. The data coordinating center will analyze and evaluate the results of the study. In addition, people from the data coordinating center and its consultants will be visiting the research sites. They will follow how the study is done, and they will be reviewing your information for this purpose. The information may also be used to meet the reporting requirements of governmental agencies.

This authorization does not have an expiration date. If you do not withdraw this authorization in writing, it will remain in effect indefinitely.

By signing this consent form, you are giving permission to use and give out the health information listed above for the purposes described above. You do not have to sign this consent form. If you choose not to sign this consent form, you will not be able to be in this research study. Your decision not to sign this consent form will not have any effect on your medical care and you will not lose any benefits or legal rights to which you are entitled. You have the right to review and copy your health information. However, if you decide to be in this study and sign this permission form, you may not be allowed to look at or copy your information until after the research is completed.

You may withdraw or take away your permission to use and disclose your health information at any time. You do this by sending written notice to the study clinician at the address on the front of this

informed consent form. If you withdraw your permission, you will not be able to continue being in this study, but you will not have any penalty or loss of access to treatment or other benefits to which you are entitled. When you withdraw your permission, no new health information which might identify you will be gathered after that date. Information that has already been gathered may still be used and given to others.

Collection of Identifiable Private Information or Identifiable Biospecimens:
The sample taken by fingerstick will be used only for the CRP test and then discarded. Fingerstick samples are not stored for later use.

Your data may be useful for other or future studies being done by researchers at the [Institution], and/or at other partner institutions. After removing all identifiers from your study records, your de-identified data may be used or shared for future research studies and with other researchers without your additional informed consent.

VOLUNTARY PARTICIPATION AND WITHDRAWAL
Your participation in this study is voluntary. You may decide not to participate or you may stop your participation at any time, without penalty or loss of benefits or medical care to which you are otherwise entitled. If you decide to leave the study, please tell the study clinician.

Your participation in this study may be stopped without your consent at any time and for any reason by the study clinician, the funder, and other regulatory authorities. Reasons you may be withdrawn from the study include: it is determined to be in your best interest, you need treatment not allowed in this study, you do not follow the study instructions, the study is stopped, or for other administrative reasons.

CONTACTS FOR QUESTIONS, COMPLAINTS, CONCERNS
If you have any questions or requests for information relating to this research study or your participation in it, or if you want to voice a complaint or concern about this research, or if you have a study related injury, you may contact [Principal Investigator] at [Phone Number] or a member of our study team, [Study Coordinator] at [Phone Number].

If you seek emergency care or hospitalization, tell the treating health care clinicians that you are in this research study.

If you have any questions about your rights as a research participant or complaints regarding this research study, or you are unable to reach the research staff, you may contact a person independent of the research team at the Biomedical Research Alliance of New York Institutional Review Board at 516-318-6877. Questions, concerns or complaints about research can also be registered with the Biomedical Research Alliance of New York Institutional Review Board at www.branyirb.com/concerns-about-research. The IRB is a committee that reviews research studies to help protect the rights and welfare of study participants.

STATEMENT OF CONSENT - SIGNATURES

By signing this form, I confirm the following:

- I have read all of this consent form.
- All of my questions have been answered to my satisfaction.

- I can leave the study at any time without giving a reason and without penalty.
- I agree to the collection, use, sharing and analysis of my personal health information and study information collected as part of this study by the research team and other authorized persons and regulatory agencies as described in this form.
- I will be given a copy of this signed and dated consent form to keep.
- I do not give up any legal rights that I would otherwise have if I were not in this study.

I voluntarily agree to participate in this study.

____

Study Participant: Name (Print) Signature Date

I have explained this research study to the study participant named above, answered all their questions to the best of my ability, believe they understand what has been explained and have consented voluntarily.

____

Person Obtaining Consent: Name (Print) Signature Date

Version A, B, C, D

Protocol #: PLACER-2021C3-24476
